# Natural speciation of nickel at the micrometer scale in serpentine (ultramafic) topsoils using microfocused X-ray fluorescence, diffraction, and absorption

**DOI:** 10.1186/s12932-018-0059-2

**Published:** 2018-08-14

**Authors:** Matthew G. Siebecker, Rufus L. Chaney, Donald L. Sparks

**Affiliations:** 1 Delaware Environmental Institute (DENIN), University of Delaware, Newark, DE 19716 USA; 2 Department of Plant and Soil Sciences, University of Delaware, Newark, DE 19716 USA; 3 Chaney Environmental, Beltsville, MD 20705 USA

**Keywords:** Nickel, Serpentine, Ultramafic, Laterite, Trace metal, Soil chemistry, EXAFS, XRD

## Abstract

Serpentine soils and ultramafic laterites develop over ultramafic bedrock and are important geological materials from environmental, geochemical, and industrial standpoints. They have naturally elevated concentrations of trace metals, such as Ni, Cr, and Co, and also high levels of Fe and Mg. Minerals host these trace metals and influence metal mobility. Ni in particular is an important trace metal in these soils, and the objective of this research was to use microscale (µ) techniques to identify naturally occurring minerals that contain Ni and Ni correlations with other trace metals, such as Fe, Mn, and Cr. Synchrotron based µ-XRF, µ-XRD, and µ-XAS were used. Ni was often located in the octahedral layer of serpentine minerals, such as lizardite, and in other layered phyllosilicate minerals with similar octahedral structure, such as chlorite group minerals including clinochlore and chamosite. Ni was also present in goethite, hematite, magnetite, and ferrihydrite. Goethite was present with lizardite and antigorite on the micrometer scale. Lizardite integrated both Ni and Mn simultaneously in its octahedral layer. Enstatite, pargasite, chamosite, phlogopite, and forsterite incorporated various amounts of Ni and Fe over the micrometer spatial scale. Ni content increased six to seven times within the same 500 µm µ-XRD transect on chamosite and phlogopite. Data are shown down to an 8 µm spatial scale. Ni was not associated with chromite or zincochromite particles. Ni often correlated with Fe and Mn, and generally did not correlate with Cr, Zn, Ca, or K in µ-XRF maps. A split shoulder feature in the µ-XAS data at 8400 eV (3.7 Å^−1^ in k-space) is highly correlated (94% of averaged LCF results) to Ni located in the octahedral sheet of layered phyllosilicate minerals, such as serpentine and chlorite-group minerals. A comparison of bulk-XAS LCF to averaged µ-XAS LCF results showed good representation of the bulk soil via the µ-XAS technique for two of the three soils. In the locations analyzed by µ-XAS, average Ni speciation was dominated by layered phyllosilicate and serpentine minerals (76%), iron oxides (18%), and manganese oxides (9%). In the locations analyzed by µ-XRD, average Ni speciation was dominated by layered phyllosilicate, serpentine, and ultramafic-related minerals (71%) and iron oxides (17%), illustrating the complementary nature of these two methods.

**Electronic supplementary material:**

The online version of this article (10.1186/s12932-018-0059-2) contains supplementary material, which is available to authorized users.

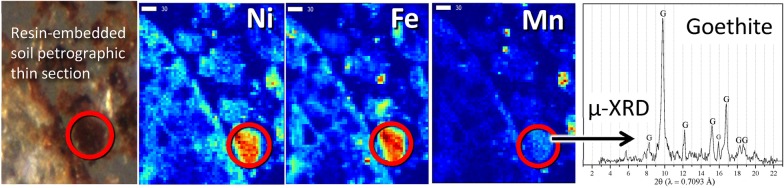

## Introduction

Serpentine soils and ultramafic laterites develop over ultramafic bedrock and are important geological materials from environmental, geochemical, and industrial standpoints. They have unique geological formation processes as compared to geographically adjacent non-serpentine soils; they possess distinct biodiversity, which is due to their particular soil chemistry [1]; their potential risks as environmental hazards have been evaluated due to naturally elevated concentrations of trace metals, such as Ni and Cr [2–4]; additionally, they may serve as potential sources of elemental Ni through harvesting hyperaccumulator plants which are endemic to them [5]. Ni is an important element for industrial purposes; it is used heavily in the production of stainless steel for construction, and the majority of land-based Ni resources come from Ni laterites [6, 7]. The implications of lateritic mining materials can indeed have significant environmental impacts [8], given that mining operations can be suspended for failing to meet environmental standards [6]. Thus, it is important to study Ni species naturally present in ultramafic soils and lateritic materials because they influence Ni mobility and transport.

In this work, microfocused spectroscopic and X-ray diffraction from synchrotron light sources was used to identify Ni mineral hosts and Ni associations with other trace metals. The natural speciation of geogenic Ni is described for three serpentine topsoils from the Klamath Mountains region in Southwest Oregon, USA. In the Klamath Mountains, serpentine soils can form from peridotite or serpentinite parent materials, and harzburgite is the dominant variety of peridotite. Geological history and maps of this region have been published [1, 9–13]. In serpentine soils, the naturally occurring minerals, elemental associations of Ni, and particle size fractions rich in trace metals are important factors that influence metal release from the soil. For example, Ni and Cr have been shown to accumulate in different particle size fractions of serpentine soils and soils enriched with serpentine minerals [14–16]. The clay particle size fraction was identified as important for serpentine minerals in several serpentine soils in the Klamath Mountains [12]. Ni mobility was higher than Cr mobility in other serpentine soils, and the type and origin of parent material, for example igneous peridotites or metamorphic serpentinites, affect Ni mobility [17]. The geochemistry of Ni in ultramafic soils is affected in particular by soil age, degree of bedrock serpentinization and mineralogy, weathering, altitude, and slope [18].

Identifying the Ni bearing minerals naturally present in the soils will improve predictions for the potential mobility of Ni because the minerals strongly affect Ni solubility [19, 20]. Knowing the mineralogical and chemical species of trace metals is important for rehabilitation of lateritic Ni mining spoils, which can potentially contaminate the environment; for example, Ni in garnierite material was associated with smectite and talc, and Ni in this phase was more exchangeable and thus more mobile than in limonitic ores where Ni was contained in the goethite lattice [8]. Additionally, Ni extraction from soils via plants depends on the mineral species present because Ni uptake is partially related to mineral solubility [21]. The possibility to extract Ni from low productivity ultramafic land via harvesting hyperaccumulator plants has also been proposed [5].

Ni soil chemistry is also affected by changes in redox conditions, where reducing conditions can cause the mobilization of Ni, whilst oxidizing conditions can immobilize Ni. This could be due to the formation of Ni-dissolved organic matter complexes at low Eh and the formation of metal hydroxides at high Eh; Ni may be immobilized in Fe and Mn (hydr)oxides via coprecipitation reactions [16]. Thus, Ni mobility can be indirectly affected by redox and pH changes. Other results have found that Ni can be mobilized in soils with low redox potential or even in oxic conditions, depending on the formation, precipitation, and/or reductive dissolution of metal hydroxides and presence of soil organic matter [22]. Although serpentine soils are high in concentrations of Cr, Ni and Co, low concentrations of these elements have been found in the surface waters of several serpentine soils; most of the Ni (> 95%) was bound in the lattice of serpentine minerals in the residual fraction of a sequential extraction procedure [3]. While surface waters may not contain elevated levels of Cr and Ni, subsurface water can become enriched with these elements and exceed international water quality standards [23].

Additionally, Ni can be transported downstream from lateritized ultramafic deposits and accumulate in mangrove sediments, where it undergoes biogeochemical redox changes dependent on depth and tide cycles; in deeper suboxic and anoxic sediments, Ni-rich goethite and Ni-talc were replaced by Ni-pyrite species; this geochemical transformation was caused by reductive dissolution of Fe(III)-minerals and subsequent sulfate reduction and pyrite formation [24]. Preservation of the anoxic zone was critical to mitigate Ni release from the sediments [25]. Variable redox conditions and weathering affect the oxidation states of Co and Mn in lateritic profiles [26], where reduced Co and Mn can commonly occur in olivine and serpentine in the bedrock. In the upper horizons of the profile, Co and Mn substituted for Fe(III) in goethite. Thus Ni, Co, and Mn, can all be scavenged by Fe-oxides in weathered laterites [26, 27].

A variety of minerals can affect Ni speciation in ultramafic soils, and Ni can correlate with various elements; using multiple tools and methods can identify the host mineral phases and elemental associations of Ni. Both bulk and microfocused X-ray techniques are examples of useful tools to identify mineral phases that contain Ni in serpentine and ultramafic lateritic soils and soil profiles [15, 27, 28]. Results from microfocused X-ray techniques which identify the elemental and mineralogical associations of Ni on the micrometer spatial scale can be coupled to results from bulk-X-ray absorption spectroscopy (XAS). Synchrotron based microfocused-XRD (µ-XRD), microfocused-X-ray fluorescence mapping (µ-XRF), and microfocused-XAS [including extended X-ray absorption fine structure (µ-EXAFS) spectroscopy and X-ray absorption near edge structure (µ-XANES) spectroscopy] are robust tools for this task [29, 30]. The objective of this research was to use these microfocused techniques to identify Ni mineral hosts and Ni associations with other trace metals such as Fe, Mn, Zn, and Cr. Microfocused-EXAFS and µ-XANES spectra were analyzed by linear combination fitting (LCF) to determine the dominant Ni species. Additionally, µ-XRD and µ-XRF data illustrate the variability of naturally occurring Ni species and distribution on the micrometer spatial scale.

## Materials and methods

Spectroscopic and diffraction data for three serpentine topsoil samples are described in this work. The samples are labeled as “s10t2”, “s11unt”, and “s20unt” and are from the Cave Junction area of Josephine County in Southwest Oregon (Klamath Mountains). These soils were chosen based on characterization results from our work employing bulk digestion, bulk-XRD, and bulk-EXAFS spectroscopy [15]. The bulk soil work indicated that soils “s20unt” and “s10t2” had the highest concentrations of Ni in our samples (Additional file 1: Table S1). Bulk-EXAFS on each particle size was also carried out on those two soils. Although “s20unt” and “s10t2” have the highest Ni concentrations, they have different textures: “s10t2” is a sandy clay loam and “s20unt” is a clay loam. The percent sand in “s10t2” is 57%, and in “s20unt” it is 34% (Additional file 1: Table S1). Lastly, soil “s11unt” contained the lowest Ni concentration of our samples from Oregon. Thus, these three samples represent several different levels of sample heterogeneity that can exist naturally in the field, including metal concentration and particle size. Soils were from field sites used to carry out experiments for Ni hyperaccumulator plants. The three soils are from the Ap horizon (0–15 cm). They were sieved to 2 mm and characterized via acid digestion and elemental analysis (Additional file 1: Table S1). Elemental composition of the soils was determined via acid digestions including microwave digestion with nitric acid (EPA method 3051), hot nitric acid (EPA method 3050B), and an Aqua Regia method; all digestion solutions were analyzed by ICP-OES. Further characterization details via bulk-XRD and bulk Ni K-edge EXAFS spectroscopy is available in the references [15]. Particle size fractionation was carried out, and petrographic thin sections were made.

For particle size fractionation, a sonication procedure was developed to separate the sand, silt, and clay particles of the soils. The procedure was the same as described in Ref. [15] with additional details given here. The initial 60 J/mL applied to the 80 mL slurry with the Branson Digital Sonifier^®^ Units Model S-450D corresponded to a time of 1 min and 14 s. The second round of sonication applied to the 150 mL of sub-250 μm fraction (440 J/mL) corresponded to 16 min 14 s; thus, an ice bath was used to maintain the temperature less than 37 °C because sonication can heat the slurry. Centrifugation times were calculated using the spreadsheet in Additional file 2, which was developed using separate equations in the soil chemical analysis advanced course [31], p 113 and p 127 and methods of soil analysis part 4, physical methods [32] and two other resources [33, 34].

For sonicated samples, µ-XRF mapping, µ-XRD, and µ-XAS were carried out on the clay, coarse silt, and medium sand fractions (that is, the sub-2 µm fraction, the 25–45 µm silt fraction, and the 250–500 µm medium sand fractions, respectively), hereafter referred to as clay, silt, and medium sand fractions. Sonicated fractions were mounted on Kapton^®^ tape via adhesion and removal of excess particles. The sonicated fractions are different from each other by about one order of magnitude.

For petrographic thin sections, whole soil fractions (air dried, < 2 mm sieved) were embedded in Scotchcast^®^ electrical resin, adhered to a trace element free quartz glass slide with a cyanoacrylate-based adhesive and ground to 30 µm thickness. For μ-XRF mapping, sufficient incident X-ray energy (10–17 keV) to simultaneously excite fluorescence from Ni and other trace elements was used to determine elemental distributions. Blank portions of the thin section were measured via both μ-XRF and μ-XRD. High-resolution photographs of the thin sections were acquired using a microscope at the National Synchrotron Light Source (NSLS) beamline X27A (Leica Microsystems). The high-resolution photographs serve as visual guides to the µ-XRF maps and provide qualitative information such as mineral morphology to accompany the quantitative spectroscopic and diffraction data.

Further materials and methods information is provided in Additional file 1. This information includes methods for µ-XAS and µ-XRF data collection and analysis in Additional file 1: Text S2.1 [35–37], µ-XRD data collection and processing in Additional file 1: Text S2.2 [38–45], a description of standards used in EXAFS and XANES fitting in Additional file 1: Text S2.3 [15, 29, 46–55], and detailed description of PCA, TT, LCF, and F-tests in Additional file 1: Text S2.4 [15, 30, 36, 37, 51, 56–63].

## Results and discussion

### Complementary X-ray diffraction and spectroscopy

**Fig. 1:**
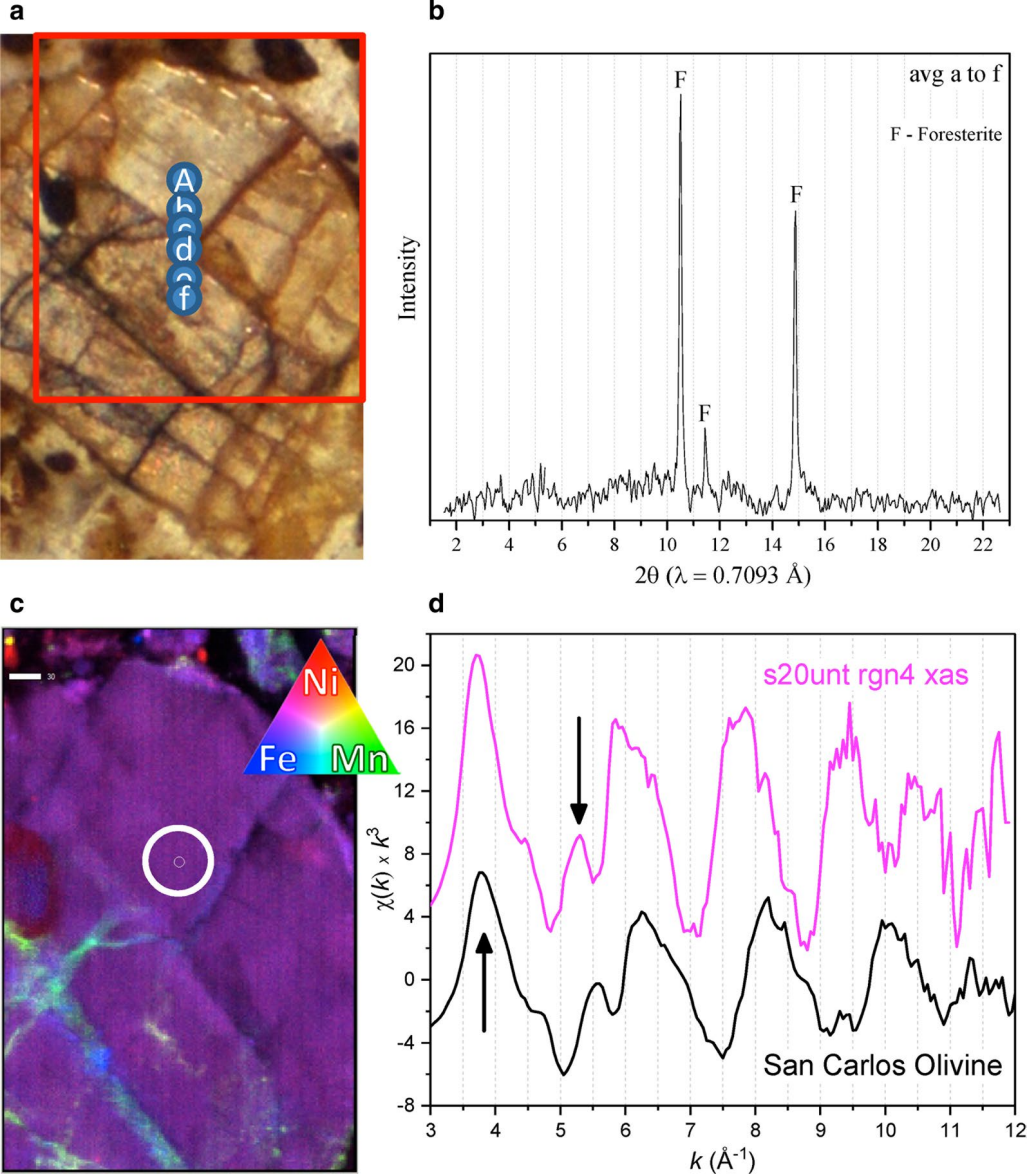
Ni distribution in forsterite. High-resolution photograph delineating the area of analysis (**a**); averaged µ-XRD spectra from points A–F (**b**); µ-XRF map (**c**); and the µ-EXAFS data obtained at the smaller white circle in the map along with Ni K-edge EXAFS of San Carlos Olivine [64] for comparison. This figure **a**–**d** was selected from Additional file 1: Figure S22 (“s20unt” region 4); the combination of microfocused techniques highlights the benefits of using multiple tools to analyze the same sample location. Here, the µ-EXAFS and µ-XRD spectra both indicate that Ni is located in forsterite, which is an olivine-series mineral

Figure 1 highlights the complementary use of µ-XRD and µ-XAS to identify solid phase minerals which contain Ni. A high-resolution photograph (Fig. 1a) shows a mineral in the petrographic thin section of sample “s20unt” region 4 upon which µ-XRF, µ-XRD, and µ-XAS were carried out. The red box on the photograph indicates the approximate boundaries of the µ-XRF map. Spots A through F indicate the locations where µ-XRD patterns were obtained. The µ-XRD patterns were averaged together to improve the signal-to-noise ratio (Fig. 1b). The tricolored µ-XRF map is shown in Fig. 1c with Ni in red, Fe in blue, and Mn in green. The µ-EXAFS spectrum was collected at the location of the smaller white circle and is shown along with a bulk-EXAFS spectrum of San Carlos Olivine for comparison in Fig. 1d. Ni K-edge bulk-EXAFS data of San Carlos Olivine [64] were digitized [65] and rebinned at 0.05 Å^−1^ in k-space.

Figure 1 serves as an example of Ni distributed in a constant and homogeneous manner throughout the solid phase of a large mineral particle (purple color in the tricolor map), which is hundreds of micrometers in the x, y directions (the scale bar is 30 μm). This mineral is off-white in color with several veins perpendicular to each other (see photograph). The veins accumulate Mn in some areas. Only three diffraction peaks were produced from the averaged μ-XRD spectra of this mineral, even though this is an average of six diffraction spectra “A–F”. The lack of multiple diffraction peaks commonly occurs in μ-XRD data (see Additional file 1: Text S2.2 for further discussion). The lack of peaks is because the sample and beam are stationary, so the X-ray beam does not reflect of all the mineral lattices. For this particular spot, both μ-XRD and μ-XAS data were collected. The diffraction peaks correspond to forsterite, which is a nesosilicate mineral in the olivine group. This was the only identification of forsterite in this work; however, forsterite was identified in the bulk and silt fractions of the “s20unt” soil [15].

Nesosilicate minerals are different from phyllosilicate minerals and inosilicate minerals because the silica tetrahedra are held together only by electrostatic forces, thus they weather readily in soils [66, 67]. Inosilicate (or chain silicate) minerals have chains of silica tetrahedra that share two corner oxygen atoms. An increasing number of chains give greater resistance to weathering. The phyllosilicate minerals contain layers of silica tetrahedra with three oxygen atoms sharing between two tetrahedra. This provides even further resistance to weathering [66]. Forsterite is a Mg-rich mineral common to ultramafic rocks. It associates with enstatite, magnetite, antigorite, and chromite [68]. Thus, its occurrence here is understandable, and Ni substitution into the olivine/forsterite structure is common.

**Fig. 2:**
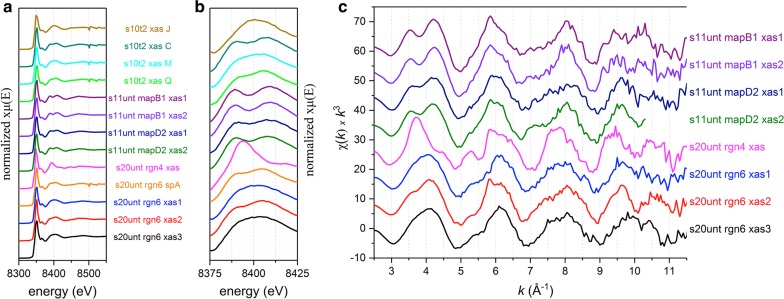
A compilation of all µ-XAS spectra. Normalized Ni K-edge µ-XANES spectra, 13 total (**a**); a close-up view of the first large oscillation close to the end of the XANES region (**b**); Ni K-edge µ-EXAFS spectra, 8 total (**c**). The spectra are shown in order of appearance in Additional file 1 and progress numerically and alphabetically with respect to sample name and regions of interest. All spectra are color coded to remain the same throughout the manuscript and Additional file 1

The physical location of the μ-EXAFS spectrum “s20unt rgn4 xas” is indicated by the small white inner circle on μ-XRF the map. Both the μ-EXAFS and μ-XANES (Fig. 2a, b) spectra from this spot display features unique to forsterite. In the μ-EXAFS spectrum, there is a steep (elongated) first peak with a maximum at ca 3.7 Å^−1^ (Fig. 1d, see arrow). The elongated peak is unique to forsterite and not seen in the other samples (Fig. 2). The elongated peak at ca 3.7 Å^−1^ is similar to other work which studied Ni distribution San Carlos Olivine [64].

Another peak of interest in the sample is at ca 5.3 Å^−1^ (ca 5.5 Å^−1^ in the San Carlos Olivine spectrum) and is indicated with another arrow. There is a distinct upward peak at this energy. The similarity of the structural features (such as peaks and shoulders) between the μ-EXAFS from this study and the bulk-EXAFS of San Carlos Olivine provides evidence of Ni incorporation into this olivine-group mineral. The phase of the major oscillations in the San Carlos Olivine spectrum is slightly longer than those seen in the μ-EXAFS data. The elongated peaks at ca 3.7 Å^−1^ line up well between the two spectra, but the next peak at arrow ca 5.3 Å^−1^ is slightly shifted to ca 5.5 Å^−1^ in the San Carlos Olivine. The slight contraction of the major oscillations in the μ-EXAFS spectrum versus the San Carlos Olivine spectrum is perhaps due to differences in the ratios of trace metals (Fe, Mn, and Ni, versus Mg) incorporated into the two different samples. The spectroscopic and diffraction data in Fig. 1 corroborate each other to show homogeneous incorporation of Ni into forsterite. The major distinguishing oscillations in μ-EXAFS spectrum at ca 3.7 and ca 5.3 Å^−1^ also match up well with those the of another forsterite mineral standard [27].

The major distinguishing oscillations of each µ-XAS spectra from all samples can be compared in Fig. 2, including both µ-XANES and µ-EXAFS spectra. In total, there are 13 µ-XANES spectra (Figs. 2a, b) and 8 µ-EXAFS spectra (Fig. 2c). The close up of the XANES region (Fig. 2b) illustrates differences in the split shoulder at 8400 eV. This split is also part of the EXAFS region, and this energy (8400 eV) translates to 3.7 Å^−1^ in the EXAFS region. At this wavenumber, a large indentation is present in the first oscillation of the spectra. Forsterite contains the elongated peak not seen in the samples. This elongated peak is at a similar location to the first peak of the split shoulder feature in other samples.

Lighter elements, such as Al atoms, allow for the appearance of the split in the first EXAFS oscillation [47], similarly to the effect of Mg atoms common in ultramafic serpentine minerals. The split can be readily seen for transition metals bound in the octahedral layer of clays and in Al-modified phyllosilicates [29, 48, 49]. Ultramafic parent materials are high in Mg; thus Mg would likely be the dominant light-weight cation in the octahedral layer. Mg concentrations for soils “s10t2”, “s11unt”, and “s20unt” were 15,700, 23,600, and 13,900 mg kg^−1^, respectively (Additional file 1: Table S1). Thus, a split shoulder at this particular energy indicates Ni incorporation into the octahedral sheet of a layered silicate mineral, such as a phyllosilicate including clinochlore or lizardite [15]. In EXAFS spectra of “Ni-rich” and “Ni-poor” serpentine minerals [27], the former lack an indentation in the first oscillation, and the latter display an indentation similar to the serpentine mineral standards used in this study.

Figures 1 and 2 illustrate the manner in which data in Additional file 1 were analyzed and facilitate simultaneous comparison of µ-XAS data from all samples, respectively. The results of each sample (including µ-XRF µ-XRD µ-XAS) are given in Additional file 1: Figures S1 through S24 along with detailed accompanying text. Figures in Additional file 1 have been summarized in Tables 1, 2, and 3, and summary discussions and conclusions are in “Summary of μ-XRD”, “Summary of μ-XRF”, and “Summary of μ-XAS”. Table 1 is a summary of all the minerals identified by µ-XRD in each sample and spectrum. Table 2 is a summary of Ni and elemental distributions in µ-XRF maps. Table 3 is a summary of all the µ-XAS data collected, including both µ-XANES and µ-EXAFS. Results from LCF of both µ-XANES and µ-EXAFS spectra are given in Table 3, while the spectral fits themselves are given in their corresponding figures in Additional file 1. In total, five spots possess both microfocused spectroscopic (µ-XAS) and diffraction data (µ-XRD).

**Table 1:**
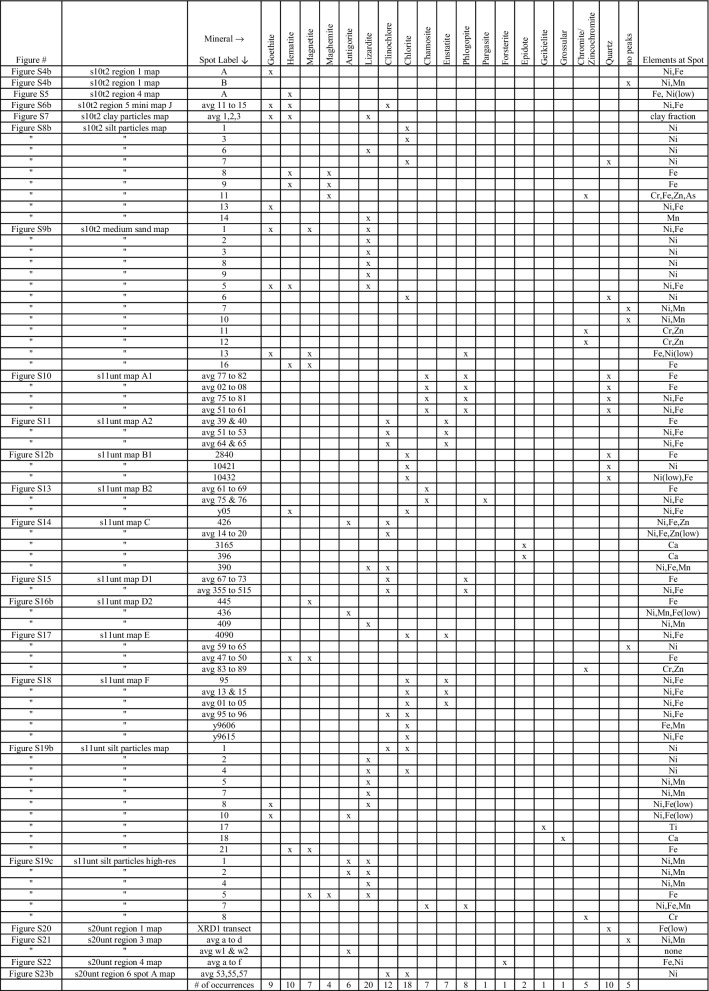
A summary of all minerals identified by µ-XRD in each sample and spectrum

In total, 74 µ-XRD figures are in Additional file 1 including 88 different spectra. Elements present at each spot are listed, and of the 88 µ-XRD spectra, 55 are from minerals that contained Ni to some degree (CPS)

**Table 2:** Summary of Ni and elemental distributions in each map

Notes on elemental distribution → sample↓	A	B	C	D	E	F	G	H
	Ni diffuse with Fe	Ni diffuse with Mn	Ni hotspot with Fe	Ni hotspot with Mn	Ni unassociated hotspots	Fe unassociated hotspots	Mn unassociated hotspots	Other unassociated hotspots
Figure S4a—s10t2 region 1 map	x		x	x	x	x		Cr
Figure S4b—s10t2 region 1 map	x		x	x				
Figure S5—s10t2 region 4 map			x	x		x	x	Cr/Zn
Figure S6a—s10t2 region 5 map	x	x		x	x	x	x	Cr
Figure S6b—s10t2 region 5 mini map J	x			x				Cr, Ti
Figure S6c—s10t2 region 5 mini map M&C	x			x	x	x	x	Cr/Zn, Ti
Figure S6d—s10t2 region 5 mini map Q		x		x		x		Cr/Zn, Ti
Figure S7—s10t2 clay particles map	x	x						
Figure S8a—s10t2 silt particles map	–	–	–	–	–	–	–	–
Figure S8b—s10t2 silt particles map			x	x	x	x	x	Cr
Figure S9a—s10t2 medium sand map	–	–	–	–	–	–	–	–
Figure S9b—s10t2 medium sand map			x	x	x	x		Cr/Zn
Figure S10—s11unt map A1	x		x					Ti
Figure S11—s11unt map A2	x		x			x	x	Cr/Zn, Ti
Figure S12a—s11unt map B1	x		x			x	x	
Figure S12b—s11unt map B1	–	–	–	–	–	–	–	–
Figure S13—s11unt map B2	x					x	x	Cr, Ti
Figure S14—s11unt map C	x		x			x	x	Ti, Ca
Figure S15—s11unt map D1	x		x			x		Zn
Figure S16a—s11unt map D2	x	x		x	x	x	x	
Figure S16b—s11unt map D2	–	–	–	–	–	–	–	–
Figure S17—s11unt map E	x			x	x	x		Cr/Zn, Ti
Figure S18—s11unt map F	x		x			x		Cr, Ti, Ca
Figure S19a—s11unt silt map	–	–	–	–	–	–	–	–
Figure S19b—s11unt silt map			x	x	x	x	x	
Figure S19c—s11unt silt high‐res				x		x		Cr/Zn, Ti, Ca
Figure S20—s20unt region 1 map	x			x	x	x		
Figure S21—s20unt region 3 map	x	x	x	x	x	x		Cr/Zn/Fe
Figure S22—s20unt region 4 map	x	x		x	x	x	x	Cr/Zn
Figure S23a—s20unt region 6 map	x	x	x	x	x	x	x	Cr/Zn, Ti
Figure S23b—s20unt region 6 spot A map	–	–	–	–	–	–	–	–
Figure S23c—s20unt region 6 spot B map	–	–	–	–	–	–	–	–
# of occurrences	19	7	14	17	12	21	12	
% of occurring	76	28	56	68	48	84	48	

Several of the maps are smaller, higher resolution maps and thus not included in the last row tallies

**Table 3:** Summary the LCF results from µ-EXAFS and µ-XANES spectra

Figure	Sample	Spot label on figure	μ-XANES	μ-EXAFS	μ-XRD	Split present	Elements at spot (via µ-XRF map)	LCF results (standards in Table S2)	R-factor from LCF	F-test value for n + 1 standards	Delta E_0_ (eV)
Figure S6b	s10t2 region 5 mini map J	xas J	x		x	no	Ni, Fe	74% iron oxide (Ni-hematite) 25% iron oxide (Ni-ferri pH7)	0.0004	5.4%	N/A
Figure S6c	s10t2 region 5 mini map M&C	xas M	x			no	Ni, Mn	75% layered serpentine mineral (Ni–Al LDH) 27% manganese oxide (NiTC birn)	0.0003	N/A	0.421 (0.013) 0.429 (0.034)
Figure S6c	s10t2 region 5 mini map M&C	xas C	x			yes	Ni, Fe (low)	83% layered serpentine mineral (Ni-serp 5811) 17% iron oxide (Ni-ferrihydrite)	0.006	N/A	N/A
Figure S6d	s10t2 region 5 mini map Q	xas Q	x			yes	Ni, Mn, Fe	50% Layered serpentine mineral (Ni-serp 5811) 49% iron oxide (Ni-hematite)	0.0005	N/A	0.468 (0.024) 0.435 (0.055)
Figure S12a	s11unt map B1	xas1	x	x	x	yes	Ni, Fe (low), Mn (low)	39% layered serpentine mineral (Ni-gibbsite) 72% layered serpentine mineral (Ni-serp 5811)	0.084	80%	N/A
Figure S12a	s11unt map B1	xas2	x	x		yes	Ni, Fe, Mn (low)	37% layered serpentine mineral (Ni-gibbsite) 74% layered serpentine mineral (Ni-serp 5811)	0.107	64%	N/A
Figure S16a	s11unt map D2	xas1	x	x	x	yes	Ni, Mn	79% layered serpentine mineral (Ni-gibbsite) 10% manganese oxide (Ni-RS birn)	0.044	28%	N/A
Figure S16a	s11unt map D2	xas2	x	x		yes	Ni	70% layered serpentine mineral (Ni-gibbsite) 30% layered serpentine mineral (Ni-serp 5811)	0.061	41%	N/A
Figure S22	s20unt region 4 map	rgn4 xas	x	x	x	no	Fe, Ni	See Fig. 1	N/A	N/A	N/A
Figure S23b	s20unt region 6 mini map A	spA	x		x	yes	Ni	67% layered serpentine mineral (Ni–Al LDH) 33% layered serpentine mineral (Ni-serp 5811)	0.0006	≪1%	0.400 (0.078) 0.400 (0.033)
Figure S23c	s20unt region 6 mini map B	xas1	x	x		no	Ni, Fe	46% layered serpentine mineral (Ni-gibbsite) 55% iron oxide (Ni-hem pH7)	0.068	9%	N/A
Figure S23c	s20unt region 6 mini map B	xas2	x	x		yes	Ni, Fe (low)	42% layered serpentine mineral (Ni-silicate) 75% layered serpentine mineral (Ni-serp 5811)	0.062	10%	N/A
Figure S23c	s20unt region 6 mini map B	xas3	x	x		no	Ni, Fe low), Mn	73% manganese oxide (Ni-RS birn) 34% layered serpentine mineral (Ni-serp 96)	0.037	8%	N/A

Samples with µ-XANES and µ-EXAFS data are identified along with those samples where complementary µ-XRD spectra were obtained. The presence of a split shoulder at 8400 eV and 3.7 Å^−1^ for µ-XANES and µ-EXAFS data, respectively, is also indicated along with the elements present at that location according the µ-XRF maps. Error values for E_0_ are adjacent in parentheses (see Additional file 1: Text S2.4.)

### Summary of µ-XRD

Data in Table 1 summarize the results from each diffractogram. Because Ni is naturally occurring in serpentine soils and lateritic profiles, it is not deposited from aerosols emitted by smelters or other anthropogenic sources. Thus, in addition to being sorbed to clay mineral surfaces, Ni is commonly incorporated into the crystal lattices of silt and sand-sized particles of the parent and secondary minerals [1, 15]. The µ-XRD data indicate that Ni was often located in the octahedral layer of serpentine minerals (for example, lizardite) and other minerals such as chlorite, which is another layered phyllosilicate mineral with octahedral structure similar to lizardite. Microfocused-XRD spots close in physical proximity but with elemental heterogeneity were commonly seen to produce similar µ-XRD patterns (Additional file 1: Figures S10–S12a, b). Enstatite, chlorite, pargasite, antigorite, lizardite, and phlogopite integrated various amounts of Ni and Fe over the micrometer scale (Additional file 1: Figures S11––S15, S16b–S18, and S23b). Enstatite is a chain inosilicate mineral also found in the bulk-XRD patterns of “s11unt” [15]. It is a ferromagnesian pyroxene mineral common to mafic rocks [1, 68]. Chlorite minerals, such as clinochlore and chamosite, were important Ni species in multiple samples. Over a 500 µm µ-XRD transect, chamosite and phlogopite illustrated large difference in elemental composition; Ni content increased six to seven times within the same transect (Additional file 1: Figure S10). Lizardite was identified multiple times as in important host for Ni. This is reasonable because Ni can substitute for Mg^2+^ in olivine, pyroxenes, and serpentine minerals [1]. Chlorite and enstatite also incorporated varying amounts of Ni and Fe in their structures, often within the same mineral (Table 1).

Microfocused-XRD was particularly useful for the sonicated silt and medium sand fractions for identification of Ni-rich minerals such as lizardite. Chlorite minerals were also commonly identified as a Ni-rich; both clinochlore and chamosite are part of the chlorite group and thus share multiple diffraction peaks. Clinochlore is a Mg–Al rich phyllosilicate and forms a solid solution series with chamosite, which is rich in Fe^2+^. It can occur in serpentinite and ultramafic rocks and associates with olivine [68]. Chlorite integrated both Fe and Mn simultaneously (Additional file 1: Figure S18) into its structure. Lizardite also simultaneously hosted Ni and Mn in its octahedral layer. Though, at discrete Ni/Mn hotspots, it was common that no diffraction peaks could be observed (Additional file 1: Figures S4b and S9b). Some improvement in diffraction patterns can be obtained by “rocking” the sample several microns under the X-ray beam in the x, y direction while collecting data. In lizardite, Ni was also independent of other trace metals (Additional file 1: Figures S16a, b, S19b). These findings agree with literature where serpentine minerals contained a relatively consistent amount of Ni. For example, in an Albanian ultramafic toposequence serpentine minerals contained about 0.3% Ni while Ni content in smectites ranged up to 4.9% [69]. The serpentine soils of this toposequence developed on serpentinized harzburgite, and harzburgite is also a common type of peridotite parent material in the serpentine soils of the Klamath Mountains [13].

Ni was associated with Fe in a variety of morphological fashions, ranging from agglomerated minerals, where a combination of hematite, clinochlore, and goethite were present (Additional file 1: Figure S6b), to larger discrete particles where Ni was in forsterite, goethite, and hematite. Goethite and hematite are common secondary Fe oxides that form during weathering processes of serpentine soils [1]. Other µ-XRD results also indicated Ni accumulation in goethite (Additional file 1: Figure S8b). Lower amounts of Ni were in hematite than in goethite on the µ-XRF maps. Goethite was identified in the silt particle size fraction (25–45 µm) together with lizardite and antigorite in the same diffractograms (Additional file 1: Figure S19b), illustrating that on the tens of micrometers scale these minerals can be closely associated and both host Ni and Fe.

Thus mixtures of Fe oxides and serpentine minerals were detected by µ-XRD; another example is in Additional file 1: Figure S9b, “spot 1” and “spot 5”. This close physical association of minerals indicates that perhaps during lizardite weathering, as Fe^2+^ leaches out it can oxidize and precipitate to form goethite. Ni accumulation in iron oxides has been found in other ultramafic profiles, for example, a lateritic regolith [27]. Ni in primary silicate minerals, such as olivine in the bedrock, was incorporated into the structures of secondary phyllosilicate minerals and iron oxides, such as serpentine and goethite, respectively. This occurred in the lower portion of the regolith (saprolite). In the upper portion of the regolith profile (the lateritic portion) Ni was principally located into the goethite structure. Manganese oxides also hosted a significant portion of Ni in the transition laterite zone [27].

It was uncommon for Ni and Zn to associate, but evidence is given for the inclusion of Zn into the layered structures of clinochlore and antigorite (Additional file 1: Figures S14); although, trace metal substitution (such as Ni, Fe, or Mn) into the antigorite structure was not always observed, such as in Additional file 1: Figure S21 where antigorite likely rich in only Mg was identified. Cr hotspots could often be identified as chromite mineral via µ-XRD (for example, Additional file 1: Figure S9b). The presence of Ti and Ca rich minerals were also identified by µ-XRD (Additional file 1: Figure S19b), illustrating the versatility of the µ-XRD technique.

### Summary of µ-XRF

The maps cover a combined 25 different regions in the samples. Several of the maps are smaller, higher resolution maps and thus not included in the summary tallies at the bottom of Table 2. In Table 2, Ni distribution was separated into five different trends which commonly occurred in the samples. In column A, “Ni diffuse with Fe” indicates Ni distribution at low but homogeneous levels over broad areas of a map. This distribution can be in Fe oxide clays or in larger mineral surfaces such as lizardite, antigorite, clinochlore, or forsterite. In column B, “Ni diffuse with Mn” indicates areas where Ni and Mn associate in amorphous regions, not bound by the edges of mineral surfaces seen in the accompanying photographs. In column C, “Ni in hotspots with Fe” indicates small, discrete areas where Ni and Fe associate. In column D, “Ni in hotspots with Mn” indicates areas where Ni and Mn associate in discrete regions typically bound by the edges of mineral surfaces. In column E, “Ni unassociated hotspots” indicates areas where Ni is not associated with other elements in the µ-XRF maps. Generally these regions are discrete, well bounded, and not amorphous. In the remaining columns (F, G, and H), other elements and elemental associations are indicated.

The tallies at the bottom of Table 2 indicate the percent of occurrences for a particular distribution trend. In 76% of the maps, Ni was associated with Fe in a diffuse manner, either with Fe oxides or in the lattice structure of larger minerals such as lizardite, antigorite, clinochlore, or forsterite. In only 28% of the observations, Ni was associated with Mn in a diffuse manner. Thus, in the µ-XRF maps, Ni was more often associated in a diffuse fashion with Fe than with Mn. This is likely due to the high content of iron and iron oxides in these soils; each soil contained goethite and/or hematite in its bulk-XRD pattern [15]. Additionally, the amount of Fe in each soil is much higher than Mn; Fe concentrations are about one order of magnitude or more than Ni for all three soils, and Ni concentrations were sometimes twice as high as Mn (Additional file 1: Table S1).

In terms of Ni hotspots with Fe or Mn, where the hotspots are discrete particles, this occurred in 56% and 68% of the 25 regions that were mapped, respectively. Reddish color in high-resolution photographs was correlated to µ-XRF data; for example, Ni correlated with red goethite particles identified by µ-XRD (Additional file 1: Figure S4a). Mn hotspots were often correlated with Ni, and often Mn was densely associated with Ni in the µ-XRF maps in both diffuse and discrete areas (Additional file 1: Figure S6d). Interestingly though, each time Ni and Mn associated densely in discrete black minerals, no or few diffraction peaks were produced (Additional file 1: Figures S4b “spot B”, Additional file 1: Figures S9b “spot 7 and 10”, and Additional file 1: Figures S21 “avg a–d”). Mn was seen to accumulate not only in veins of larger minerals (Figs. 1 and Additional file 1: Figure S22) but also discretely inside the bulk of minerals and within agglomerated Fe oxides. However, it is not necessary that Ni associate with any trace metals; 48% of the mapped regions contained unassociated Ni hotspots. The abundance of Fe in these samples, in terms of Fe oxide clays and minerals such as goethite and magnetite, yielded a high occurrence of unassociated Fe hotspots (84%). Lastly, 48% of the regions contained unassociated Mn hotspots. Thus in different locations, Ni, Fe, and Mn were associated together and also distributed independently of each other; their trends were categorized into eight groups (A–H) in Table 2.

Ni generally did not associate with Cr, Zn, Ca, or K. Though, Zn correlated with several Cr hotspots. Ni and Cr essentially never correlated with each other in the µ-XRF maps. The exception to Ni and Cr correlation was in the clay fraction of “s10t2” (Additional file 1: Figure S7) where no resolution of discrete particles was possible from the µ-XRF maps. The clay size fraction contains particles (≤ 2 µm) that are smaller than the X-ray beam (2 µm at SSRL). Information on elemental distributions cannot be gleaned when particle sizes are smaller than the beam, which can also be caused by grinding samples in a mortar/pestle. Thus for samples used in this study it is not recommended to grind samples because this can homogenize the sample and prevent correlations of different elements. A useful aspect of µ-XRF mapping is that elements in the maps can be used to eliminate mineral hosts with similar matching diffraction peaks but which are not compatible given the fluorescing elements. Additionally, the µ-XRF maps can be used to limit the number of standards used in LCF. For example, if a µ-XRD or µ-EXAFS spectrum was obtained from a spot high in Ni and Mn fluorescence but very low in Fe, all the Fe oxide mineral standards (goethite, ferrihydrite, magnetite, et cetera) could be excluded from matching peaks or LCF routine, respectively.

### Summary of µ-XAS

Table 3 is a summary of the µ-XAS data and LCF results. Ni speciation was dominated by serpentine mineral standards, such as lizardite, and Ni bound (either via surface adsorption or precipitation/incorporation into mineral structure) with iron oxides, such as goethite, hematite, and ferrihydrite. In seven of the eight spectra that displayed a split shoulder feature at 8400 eV, there is a decrease the counts per second (CPS) of Fe or Mn or low overall CPS of Fe, Mn, or Ni. When other trace metals such as Fe and Mn are low and Ni is the predominant fluorescing metal in the µ-XRF maps, the split shoulder generally occurs. Spectral features in the µ-XANES and µ-EXAFS data, such as the split at 8400 eV and 3.7 Å^−1^, respectively, indicate that Ni is located in the octahedral layers of phyllosilicate minerals such as lizardite or a chlorite-group mineral; this is confirmed by µ-XRD in Additional file 1: Figures S12 spot “B1xas1”, Additional file 1: Figures S16 spot “D2xas1”, and Additional file 1: Figures S23b “spA”.

The presence of the split can be used to identify this specific type of local atomic environment. Ni is octahedrally coordinated with oxygen in a sheet and has lighter elements such as Mg as the dominant second nearest neighbors (for example, Ni–O–Mg). Mg dominates as the light element in lizardite [Mg_3_Si_2_O_5_(OH)_4_]. This split shoulder is clearly visible in lizardite mineral standards [15], and it is common for trace metals in phyllosilicates [70–74]. The split shoulder can often occur where trace metals such as Ni or Zn are present in phyllosilicates [15, 29]. See references [47–49] for more discussion on the formation of this split shoulder feature.

When LCF results are averaged together for the eight spectra with the split shoulder (Fig. 2), 94% of the averaged species can be attributed to standards in the “Layered Serpentine Mineral” category. Thus, this split shoulder is highly correlated to Ni located in the octahedral sheet of a layered mineral. In Additional file 1: Text S2.3, this category is described and includes ultramafic serpentine mineral standards, layered silicates, adsorbed and precipitated Ni-rich phases that form octahedral sheets over time, and layered single and double metal hydroxides. When Fe or Mn is present at higher CPS with Ni, this split shoulder disappears because either the second nearest neighbor to Ni is mostly Mn or Fe in an octahedral layer, or Ni is associated with Fe or Mn oxides, where the split shoulder does not occur. Using XANES data alone, it can be difficult to identify Ni species when Ni occurs with Mn in the same hotspot. This is because Mn rich minerals, such as manganese oxides, and Mn rich serpentine minerals both lack the split shoulder at 8400 eV. When the amount of heaver elements such as Mn, Fe, Zn, or Ni increases in the second nearest neighboring shell, the split disappears [29, 75]. This disappearance is also evident in examples of “Ni-rich” and “Ni-poor” serpentine minerals [27].

For example in Additional file 1: Figure S6c at spot “M”, because Mn (Z = 25) is heavier than Mg (Z = 12) no splitting would occur if Ni were present in chlorite. Ni could be associated with a layered Mn oxide, such as birnessite, or a layered phyllosilicate mineral such as chlorite, which can be heavily substituted with Mn in the octahedral layer. The LCF results agree with this hypothesis because the manganese oxide standards were consistently ranked as important components in the best fits for this spot. The final fit however included NiAl-LDH (75%) and Ni sorbed to triclinic birnessite (NiTC Birn 27%). This result does not mean that NiAl-LDH is the actual species in the sample; rather, the NiAl-LDH standard is being used as an analogue for another Ni-rich layered mineral where Ni is in the octahedral sheet, such as lizardite or a chlorite-group mineral. The NiAl-LDH standard is representative of Ni in the 2 + oxidation state, octahedrally coordinated by ~ 6 oxygen atoms, and located in the octahedral sheet of a layered mineral, which are three characteristics that make it a good analogue for Ni substituted into a serpentine mineral. Thus at spot “M”, Ni is likely associated with a Mn-rich serpentine mineral. Another example where there is a decrease in the split shoulder is in Additional file 1: Figure S23b, where Ni is the only dominant fluorescing trace metal; the split is not as pronounced as in other spectra likely because of the relatively high Ni CPS which would be found in a Ni-rich phyllosilicate mineral.

By averaging the µ-XAS LCF results from both µ-EXAFS and µ-XANES, a comparison was made to bulk-XAS LCF results previously published [15] for these three soils. This comparison helps to determine if the microfocused data are representative of the bulk soil. Bulk-XAS LCF results showed higher Fe-oxide contents in “s10t2” than in other samples [15]. The averaged µ-XAS LCF data yielded a similar result; of the three soils, “s10t2” also has the highest percentage of Fe oxides; the “Iron Oxides” category composed 41% of all “s10t2” fits, while the “Layered Serpentine Minerals” category was 52%, and the “Manganese Oxides” category was 7%. Additional file 1: Text S2.3 discusses the categories for each standard. In the bulk-LCF XAS results for “s10t2”, Fe oxides were 42%, serpentine and ultramafic minerals were 23%, and Ni adsorbed to phyllosilicates composed 34% [15]. Ni adsorbed to phyllosilicates was not identified by LCF of the µ-XAS data.

Differences in averaged µ-XAS LCF versus bulk-XAS LCF can be influenced by sampling bias. Inadvertently producing sampling bias in microfocused work can be caused by only obtaining data from “hotspots” of the element of interest. For this work, different morphological and elemental associations of Ni including diffuse and dense associations and various metal amounts (that is, CPS) were analyzed to decrease sampling bias and obtain a more representative view of Ni speciation. These morphologies and elements are identified in Tables 1 and 2. Microfocused-XRF maps from petrographic thin sections helped to discern between Ni sorbed to clay minerals such as Fe oxides and larger mineral phases based on the morphology of the fluorescence pattern in relation to the high-resolution photographs.

For “s11unt”, averaging the µ-XAS LCF results determined that “layered serpentine minerals” composed 100% of the fits while “Manganese Oxides” just 3%. The total value is over 100%, which is possible as explained in Additional file 1: Text S2.4. These averages for “s11unt” are similar to those for averaged bulk-XAS LCF, where serpentine minerals composed 83% to 96% of the bulk XAS spectra [15]. Thus for “s11unt”, there is good representation of the bulk soil and sample heterogeneity via the µ-XAS technique. Lastly, for “s20unt”, because of spectral similarities between Mn oxide standards and other standards, the bulk-XAS LCF value of the Mn oxide component was artificially increased [15], which made it quite different than the averaged µ-XAS LCF results of “s20unt”. For averaged µ-XAS LCF of “s20unt”, 74% of the fits could be attributed to “layered serpentine minerals”, 14% to “Iron Oxides”, and 18% to “Manganese Oxides”. Thus there was good representation of the bulk soil via the µ-XAS technique for two of the three soils.

In terms of combined LCF results from all three soils, averaged µ-XAS LCF values from all the fits indicated that standards in the “layered serpentine minerals” category consistently dominated, and on average they contributed to 76% of all LCF. Thus, for all locations analyzed by µ-XAS LCF, Ni speciation was dominated by layered phyllosilicate and serpentine minerals (76%), with smaller contributions on average from iron oxides (18%) and manganese oxides (9%).

## Conclusion

On an 8 µm spatial scale, Ni and Mn were simultaneously present in lizardite and antigorite from µ-XRD patterns. Elemental fluorescence delineated and matched mineral morphology from high-resolution photographs. Elemental distributions (for example, the fluorescence of Fe, Mn, and Ni) aligned between maps obtained from two different beamlines (SSRL and NSLS). Data also indicate on the micrometer scale that serpentine minerals (for example, lizardite) can become embedded within larger iron oxide particles (for example, hematite). Additionally, diffraction peaks with goethite, magnetite, and lizardite were identified in the same µ-XRD spectrum, indicating that these minerals also can mix (associate) together on the micrometer scale.

Microfocused-XRD is a rapid method to accurately identify minerals that contain trace metals, and this work particularly highlights how µ-XRD can be a key investigative tool for identification of these minerals. The benefits of µ-XRD are that clear and discrete diffraction peaks can be matched with mineral phases in a prudent fashion and correlated to elements, such as Fe, Mn, Ni, Zn, and Cr in the µ-XRF maps. A more comprehensive and accurate dataset for Ni speciation was possible by combining µ-XRD with µ-XAS. The broader geochemistry communities which focus on trace metal speciation in geological materials including soils and sediments using these microfocused techniques can find useful examples here of how to couple µ-XAS and µ-XRD together.

Previous work on these and other related serpentine soil samples focused on bulk physicochemical characterization and bulk-EXAFS spectroscopy to characterize Ni in the whole soil and various particle size fractions [15]. The current work takes a different approach and had the objective to identify minerals which integrate Ni and Ni associations with other metals such as Fe, Mn, Zn, and Cr on the micrometer spatial scale. Of all the diffractograms analyzed for this work (over 500) and the resulting µ-XRD spectra (88 total), a general summary can be made for Ni association with different mineral phases. Of the 88 µ-XRD spectra, 55 of those are from minerals that contained Ni to some degree, either low or high CPS (Table 1). From those 55 spectra, 93 minerals were identified; often the same mineral was identified multiple times. For example, goethite was identified 9 times, and those 9 times it was present with Ni (Table 1). Taking the 93 minerals in which Ni was found and grouping those minerals into the categories used for LCF (Additional file 1: Text S2.3), we find good agreement between averaged µ-XAS data and µ-XRD data. For example, goethite, hematite, and magnetite are all iron oxides, and in total, iron oxides composed 17% of all minerals which hosted Ni as identified via µ-XRD. This is very similar to the 18% determined by the average of all µ-XAS LCF results “Summary of μ-XAS”. Similarly, the rest of the minerals (from antigorite to forsterite in Table 1) are all serpentine and ultramafic related minerals; those minerals grouped together accounted for 71% of all Ni-rich minerals identified via µ-XRD. This value is very similar to the 76% of Ni associated with the “Layered Serpentine Minerals” category calculated by averaged µ-XAS LCF results.

These minerals, whether iron oxides or layered phyllosilicates such as lizardite or chlorite-group minerals, affect Ni release into solution and Ni mobility in the environment. These results are useful to researchers in the Ni hyperaccumulation community, researchers studying ultramafic laterites and regoliths, serpentine parent materials and their geochemical weathering products, or trace metal release from serpentine soils. These are all important current and future research areas; characterizing the naturally occurring minerals which host Ni is essential to understanding the relationship between serpentine soils, metal hyperaccumulating plants, trace metal mobility, and environmental risk. Further research on these soils using selective dissolution techniques and desorption kinetics studies while varying redox conditions would assist in linking Ni release and mobility to the dominant Ni species in the solid phase.

## Additional files

**Additional file 1: Text S1.** Organization of this Additional file 1. **Text S2.** Materials and Methods. **Text S2.1.** µ-XAS and µ-XRF data collection. **Text S2.2.** µ-XRD data collection and processing. **Text S2.3.** Description of Standards. **Text S2.4.** PCA, TT, LCF, and F-Test. **Figure S1.** sample “s10t2” thin section photograph overview of maps. **Figure S2.** sample “s11unt” thin section photograph overview of maps. **Figure S3.** sample “s20unt” thin section photograph overview of maps. **Figure S4a.** s10t2 region 1 map. **Figure S4b.** s10t2 region 1 map (cont.) with μ-XRD. **Figure S5.** s10t2 region 4 map with μ-XRD. **Figure S6a.** s10t2 region 5 map with μ-XANES. **Figure S6b.** s10t2 region 5 mini map J with μ-XRD & μ-XANES. **Figure S6c.** s10t2 region 5 mini map M&C with μ-XANES. **Figure S6d.** s10t2 region 5 mini map Q with μ-XANES. **Figure S7.** s10t2 clay particles map with μ-XRD. **Figure S8a.** s10t2 silt particles map. **Figure S8b.** s10t2 silt particles map (cont.) with μ-XRD. **Figure S9a.** s10t2 medium sand particles map. **Figure S9b.** s10t2 medium sand particles map (cont.) with μ-XRD. **Figure S10.** s11unt map A1 with μ-XRD. **Figure S11.** s11unt map A2 with μ-XRD. **Figure S12a.** s11unt map B1 with μ-XANES and μ-EXAFS. **Figure S12b.** s11unt map B1 (cont.) with μ-XRD. **Figure S13.** s11unt map B2 with μ-XRD. **Figure S14.** s11unt map C with μ-XRD. **Figure S15.** s11unt map D1 with μ-XRD. **Figure S16a.** s11unt map D2 with μ-XANES and μ-EXAFS. **Figure S16b.** s11unt map D2 (cont.) with μ-XRD. **Figure S17.** s11unt map E with μ-XRD. **Figure S18.** s11unt map F with μ-XRD. **Figure S19a.** s11unt silt particles map. **Figure S19b.** s11unt silt particles map (cont.) with μ-XRD. **Figure S19c.** s11unt silt particles high-resolution map with μ-XRD. **Figure S20.** s20unt region 1 map with μ-XRD. **Figure S21.** s20unt region 3 map with μ-XRD. **Figure S22.** s20unt region 4 map with μ-XRD, μ-XANES, and μ-EXAFS. **Figure S23a.** s20unt region 6 map. **Figure S23b.** s20unt region 6 mini map A with μ-XRD and μ-XANES. **Figure S23c.** s20unt region 6 mini map B with μ-XANES and μ-EXAFS. **Figure S24.** EXAFS and XANES standards spectra, see references in Table S2. **Table S1.** Physicochemical Characteristics of Soil Samples. **Table S2.** Standards used in LCF.

**Additional file 2.** Clay centrifugation calculations.

## Abbreviations

µ-XRF: synchrotron based microfocused-X-ray fluorescence mapping
µ-XRD: synchrotron based microfocused-X-ray diffraction
µ-XAS: synchrotron based microfocused -X-ray absorption spectroscopy
µ-EXAFS: microfocused extended X-ray absorption fine structure spectroscopy
µ-XANES,: microfocused X-ray absorption near edge structure spectroscopy
LCF: linear combination fitting
PCA: principal component analysis
TT: target transformation
CPS: counts per second
CCD: charge-coupled device
SSRL: Stanford Synchrotron Radiation Lightsource
NSLS: National Synchrotron Light Source
USA: United States of America
