# Supplementary material for: Natural speciation of nickel at the micrometer scale in serpentine (ultramafic) topsoils using microfocused X-ray fluorescence, diffraction, and absorption
Source: Geochem Trans. 2018 Aug 14;19:14. doi: 10.1186/s12932-018-0059-2 (PMC6091439; doi:10.1186/s12932-018-0059-2)
Supplement: Supplementary file 1 — Additional file 1: Text S1. Organization of this Additional file 1. Text S2. Materials and Methods. Text S2.1. µ-XAS and µ-XRF data collection. Text S2.2. µ-XRD data collection and processing. Text S2.3. Description of Standards. Text S2.4. PCA, TT, LCF, and F-Test. Figure S1. sample “s10t2” thin section photograph overview of maps. Figure S2. sample “s11unt” thin section photograph overview of maps. Figure S3. sample “s20unt” thin section photograph overview of maps. Figure S4a. s10t2 region 1 map. Figure S4b. s10t2 region 1 map (cont.) with μ-XRD. Figure S5. s10t2 region 4 map with μ-XRD. Figure S6a. s10t2 region 5 map with μ-XANES. Figure S6b. s10t2 region 5 mini map J with μ-XRD & μ-XANES. Figure S6c. s10t2 region 5 mini map M&C with μ-XANES. Figure S6d. s10t2 region 5 mini map Q with μ-XANES. Figure S7. s10t2 clay particles map with μ-XRD. Figure S8a. s10t2 silt particles map. Figure S8b. s10t2 silt particles map (cont.) with μ-XRD. Figure S9a. s10t2 medium sand particles map. Figure S9b. s10t2 medium sand particles map (cont.) with μ-XRD. Figure S10. s11unt map A1 with μ-XRD. Figure S11. s11unt map A2 with μ-XRD. Figure S12a. s11unt map B1 with μ-XANES and μ-EXAFS. Figure S12b. s11unt map B1 (cont.) with μ-XRD. Figure S13. s11unt map B2 with μ-XRD. Figure S14. s11unt map C with μ-XRD. Figure S15. s11unt map D1 with μ-XRD. Figure S16a. s11unt map D2 with μ-XANES and μ-EXAFS. Figure S16b. s11unt map D2 (cont.) with μ-XRD. Figure S17. s11unt map E with μ-XRD. Figure S18. s11unt map F with μ-XRD. Figure S19a. s11unt silt particles map. Figure S19b. s11unt silt particles map (cont.) with μ-XRD. Figure S19c. s11unt silt particles high-resolution map with μ-XRD. Figure S20. s20unt region 1 map with μ-XRD. Figure S21. s20unt region 3 map with μ-XRD. Figure S22. s20unt region 4 map with μ-XRD, μ-XANES, and μ-EXAFS. Figure S23a. s20unt region 6 map. Figure S23b. s20unt region 6 mini map A with μ-XRD and μ-XANES. Figure S23c. s20unt region 6 mini map B with μ-XANES and μ [file 12932_2018_59_MOESM1_ESM.pdf]

Additional file #1 for
Geochemical Transactions

Natural Speciation of Nickel at the Micrometer Scale in Serpentine (Ultramafic) Topsoils using Microfocused X-ray Fluorescence, Diffraction, and Absorption

Matthew G. Siebecker[*,a,b], Rufus L. Chaney[c], Donald L. Sparks[a,b]

[a] University of Delaware, Delaware Environmental Institute (DENIN), Newark, DE, 19716, USA.

[b] University of Delaware, Department of Plant and Soil Sciences, Newark, DE, 19716, USA.

[c] Chaney Environmental, Beltsville, MD 20705, USA.

[*]Correspondence to: mgs@udel.edu

78 Pages, 36 Figures, 2 Tables

Contents

Text S1 ....Organization of Additional file 1 ... 3
Text S2 ....Materials and Methods ... 4
Text S2.1.............μ-XAS and μ-XRF data collection ... 4
Text S2.2.............μ-XRD data collection and processing ... 4
Text S2.3.............Description of Standards ... 5
Text S2.4.............PCA, TT, LCF, and F-Test ... 6

Figure S1 ....sample “s10t2” thin section photograph overview of maps ... 9
Figure S2 ....sample “s11unt” thin section photograph overview of maps ... 10
Figure S3 ....sample “s20unt” thin section photograph overview of maps ... 11
Figure S4a...........s10t2 region 1 map ... 12
Figure S4b ....s10t2 region 1 map (cont.) with μ-XRD ... 13
Figure S5 ....s10t2 region 4 map with μ-XRD ... 15
Figure S6a...........s10t2 region 5 map with μ-XANES ... 17
Figure S6b ....s10t2 region 5 mini map J with μ-XRD & μ-XANES ... 19
Figure S6c...........s10t2 region 5 mini map M&C with μ-XANES ... 21
Figure S6d ....s10t2 region 5 mini map Q with μ-XANES ... 23
Figure S7 ....s10t2 clay particles map with μ-XRD ... 25
Figure S8a...........s10t2 silt particles map ... 27
Figure S8b ....s10t2 silt particles map (cont.) with μ-XRD ... 28
Figure S9a...........s10t2 medium sand particles map ... 30
Figure S9b ....s10t2 medium sand particles map (cont.) with μ-XRD ... 31
Figure S10 ....s11unt map A1 with μ-XRD ... 33
Figure S11 ....s11unt map A2 with μ-XRD ... 35
Figure S12a.........s11unt map B1 with μ-XANES and μ-EXAFS ... 37
Figure S12b ....s11unt map B1 (cont.) with μ-XRD ... 38
Figure S13 ....s11unt map B2 with μ-XRD ... 41
Figure S14 ....s11unt map C with μ-XRD ... 43
Figure S15 ....s11unt map D1 with μ-XRD ... 45
Figure S16a.........s11unt map D2 with μ-XANES and μ-EXAFS ... 47
Figure S16b ....s11unt map D2 (cont.) with μ-XRD ... 48
Figure S17 ....s11unt map E with μ-XRD ... 51
Figure S18 ....s11unt map F with μ-XRD ... 53
Figure S19a.........s11unt silt particles map ... 55
Figure S19b ....s11unt silt particles map (cont.) with μ-XRD ... 56
Figure S19c.........s11unt silt particles high-resolution map with μ-XRD ... 57
Figure S20 ....s20unt region 1 map with μ-XRD ... 60
Figure S21 ....s20unt region 3 map with μ-XRD ... 62
Figure S22 ....s20unt region 4 map with μ-XRD, μ-XANES, and μ-EXAFS ... 64
Figure S23a.........s20unt region 6 map ... 66
Figure S23b ....s20unt region 6 mini map A with μ-XRD and μ-XANES ... 68
Figure S23c.........s20unt region 6 mini map B with μ-XANES and μ-EXAFS ... 70
Figure S24 ....EXAFS and XANES standards spectra, see references in Table S2 ... 73

Table S1.............Physicochemical Characteristics of Soil Samples ... 74
Table S2.............Standards used in LCF ... 75

References in Additional file 1 ... 76

Text S1: Organization of Additional file 1

Microscale heterogeneity of Ni species, that is, Ni distribution amongst different minerals and its association with various trace elements, is illustrated via data from petrographic thin sections and particle size fractions of three serpentine soil samples. The samples are labeled as “s10t2”, “s11unt”, and “s20unt” and are presented in that order via a series of μ-XRF maps with accompanying μ-XRD and μ-XAS spectra. Because of the large physical dimensions and quantity of μ-XRF, μ-XRD, and μ-XAS figures, the majority of them are placed here in Additional file 1 along with a discussion for each figure. Summary results and discussion (specifically, Figures 1 and 2, and Tables 1, 2 and 3) are located in the main text.

In Additional file 1, there are 36 total figures, starting from Figure S1 and ending with Figure S24. The figures progress numerically and alphabetically with respect to sample name, regions of interest, and spots of interest where μ-XRD and/or μ-XAS were carried out. For example, the data progress from sample “s10t2” to “s11unt” and finally “s20unt”; initially, Figures S1, S2, and S3 are the photographic guides of the petrographic thin sections for each soil sample, respectively. They are overview and high-resolution photographs. The high-resolution photographs in Figures S1, S2, and S3 illustrate the regions of interest selected for examination via microfocused methods (that is, μ-XRD, μ-XRF, and μ-XAS). The Additional file 1 figures for sample “s10t2” include Figures S4a through S9b. The Additional file 1 figures for sample “s11unt” are Figures S10 through S19c. The Additional file 1 figures for sample “s20unt” are Figures S20 through S23c. The final Additional file 1 Figure S24 contains the EXAFS and XANES spectra of all the standards used in linear combination fitting (LCF), whose references and descriptions are given in Table S2 and ref. (1).

Text S2: Materials and Methods

Text S2.1.) μ-XAS and μ-XRF data collection

Microfocused X-ray techniques, including μ-XANES, μ-EXAFS, μ-XRF mapping, and μ-XRD, were conducted at NSLS microprobe beamline X27A and the Stanford Synchrotron Radiation Lightsource (SSRL) microprobe beamline (BL) 2-3. The electron beam storage ring energy was 2.5–2.8 GeV with a maximum beam current of 300 mA at the NSLS and 3 GeV with a maximum beam current of 300 mA at SSRL. Harmonic X-ray elimination was achieved by detuning the monochromators about 30-35%. The monochromators were Si(111) at NSLS and Si(111) or Si(220) at SSRL. The fluorescence detectors at X27A and BL2-3 were a 13-element germanium array detector and Vortex Si-drift detector, respectively. X-ray beam sizes were 15 μm by 10 μm (X27A) and 2 μm by 2 μm (BL2-3).

All XAS data are from the Ni K-edge energy with the monochromator calibrated to 8333 eV. Microfocused-XAS spectra were collected from 200 eV below the absorption edge energy to k values of 10-12.5 $Å^{-1}$ in the EXAFS region. Multiple scans were collected until satisfactory signal to noise ratios were achieved (typically 15-30 scans). Fluorescence detectors were positioned 90° to the incident beam (45° to the sample). Special attention was paid to selecting the fluorescence of the Ni Kα peak on the multi-channel analyzer display so that the resulting spectra excluded as much fluorescence from the Fe Kβ peak as possible. Larger μ-XRF maps (1 mm^2 to 2 mm^2) were created to identify regions of interest for μ-XAS and μ-XRD analysis. Fine μ-XRF maps were generated where necessary within larger maps on regions of interest to increase resolution. Scanning rates and dwell times varied depending on the specific beamline detector, sample stage motors, and beam size.

On several occasions in the μ-XRF maps from NSLS, Cr Kβ fluorescence was included in the Mn Kα fluorescence multi-channel analyzer display, and thus Cr appears as Mn in several μ-XRF maps. However, each occurrence of this is indicated specifically on its respective μ-XRF map. This does not occur with the SSRL maps because Cr Kβ florescence was subtracted out of Mn Kα florescence using the MicroAnalysis Toolkit (2). This subtraction procedure was not available with the data from NSLS maps. Tricolored maps for Ni, Fe, and Mn are shown with their respective maximums for counts per second (CPS) for each element, which can be different from the CPS shown in μ-XRF maps for each individual elements.

For comparison, the μ-XRD patterns took a fraction of the time to collect versus the μ-XAS data. A series of μ-XRD patterns could be obtained in as little as five minutes, and a single diffraction pattern in as little as one minute. However, to obtain high signal-to-noise ratios for μ-EXAFS spectra, multiple hours (for example, up to 5 hours) are necessary. This amount of time for an X-ray beam to be focused on a single, micrometer-sized spot on a sample would damage many kinds of biological samples or redox sensitive samples. For example, oxidation of organic carbon could occur or changes in oxidation state for redox sensitive elements, such as Cr, Mn, or As, could also occur. Beam damage did not occur in samples presented here; each scan was inspected for shifts in the edge jump positions and none were found. Additionally, spectral features in the XANES and EXAFS regions were consistent throughout all scans. The stability of these samples is likely due to Ni remaining in the 2+ oxidation state and its presence in solid phase minerals lacking organic carbon.

Text S2.2.) μ-XRD data collection and processing

Microfocused-XRD patterns were collected with CCD detectors at microprobe beamlines (NSLS X27A and SSRL BL2-3). The data were calibrated using reference diffraction patterns obtained at each beamline and integrated using Fit2D (3, 4). The X-ray wavelength (energy) used to collect μ-XRD measurements is located on the *x*-axis of each diffractogram. At the NSLS this wavelength was 0.7093 Å (17479 eV), and at SSRL it was 0.72932 Å (17000 eV). Diffraction peaks were identified using Match! (5), which uses the Crystallography Open Database (6, 7) and The American Mineralogist Crystal Structure Database (8). To improve background subtraction of μ-XRD data, the ECDSOFT program (9, 10) was used. This background subtraction program fits a spline function to user-defined background points in the spectra, which allowed for enhanced peak identification. ECDSOFT was designed for treatment and background subtraction of electrochemical data, but the software can accept data that is put into an acceptable columnar file format. Averages of two or more individual μ-XRD spectra are noted as such in their sample names. Averaging was carried out in Origin graphing and analysis software. Locations of μ-XRD transects are identified by white circles and/or dashed transect lines.

Insets in μ-XRD figures identify disproportionately large diffraction peaks, which can commonly occur with μ-XRD data. This occurs because the mineral/sample is stationary, and the angle to the incident X-rays does not change. Sometimes only several of the mineral lattices diffract the X-rays causing disproportionately large peaks and the absence of other peaks. The peaks can be much more intense because of focused diffraction into the CCD camera from mineral lattices with preferential scattering orientation. This is different from a bulk, powder mounted XRD sample, where homogeneity of the sample and random distribution of the lattices can be ensured during sample preparation and mounting.

It is emphasized that many of the μ-XRD spectra collected for this study were done at NSLS X27A from resin-embedded petrographic thin sections of soil samples mounted on trace metal free quartz glass slides (see Section 2.1). However, often the glass slide is thick enough to attenuate many diffracting X-rays, which prevents analysis of diffraction data. This was not a significant issue for this study. μ-XRD data were also collected from different particle size fractions at SSRL BL 2-3 (clay, silt, medium sand, see Section 2.1), which produced high-quality diffraction data. Often, peak-matching is challenging because minerals can have overlapping peaks, and the number and intensity of the peaks can be disproportionate to the reference database. However, the elemental data from μ-XRF maps can be useful to eliminate irrelevant matching mineral. Because μ-XRD requires only minutes to acquire a diffractogram, versus the several hours for μ-XAS scanning, literally hundreds of μ-XRD spectra from different locations (spots) can be collected in the same amount of time required to collect μ-XAS spectra for one spot. In total, there are 88 μ-XRD spectra presented in 74 graphs in Additional file 1. The μ-XRD spectra and figures presented are products of over 500 total μ-XRD spectra that were collected and analyzed.

Text S2.3.) Description of Standards

To organize LCF results, EXAFS standards were grouped into three different categories: 1) “Iron Oxide”, 2) “Manganese Oxide”, and 3) “Layered Serpentine Mineral” similarly to ref. (1). The category “Layered Serpentine Mineral” includes ultramafic serpentine mineral standards and other standards with structurally similar layered octahedral sheets that can host both trace metals such as Ni, Fe, and Mn, and lighter elements such as Al or Mg. This category includes serpentine minerals, layered silicates, adsorbed and precipitated Ni-rich phases that form

octahedral sheets over time, and layered single and double metal hydroxides. See Table S2 for a description and the category assigned to each standard. Ni adsorbed to gibbsite (11) is included in the "Layered Serpentine Mineral" category (Ni-gibbsite) because it is structurally similar to (and thus a good analogue for) Ni incorporated into the octahedral layer of serpentine minerals such as lizardite; in the Ni-gibbsite standard, Ni is bound adjacently to lighter (that is, a lower Z element) Al atoms of the gibbsite sheet. Al atoms allow for the appearance of the peak splitting of the first EXAFS oscillation (12), which can also be caused by Mg atoms commonly found in serpentine minerals. This splitting can be readily seen for transition metals bound in the octahedral layer of clays and in Al-modified phyllosilicates as well (13-15). The categories "Iron Oxide" and "Manganese Oxide" include standards where Ni is either surface adsorbed or incorporated into the structure of the iron or manganese oxides, respectively. All EXAFS standards, both synthetic and naturally occurring, which were considered for fitting have been previously characterized and published in the literature. The sources of EXAFS standards used for LCF are acknowledged here and in Table S2 (11, 16-22). Further descriptions for each standard is also available in ref. (1).

Text S2.4.) PCA, TT, LCF, and F-Test

Analysis of µ-XAS data was carried out using both Sixpack and Demeter software packages (23, 24). All raw data of samples and standards were loaded into Demeter or Sixpack for background subtraction, normalization, calibration, and alignment. Germanium detector dead-time files were applied to fluorescence data where necessary. Twenty six standards (Table S2) were used to carryout LCF on a k-range of 3 to 10 (except for the last spectrum, as indicated, where a k-range 3 to 9 was used). Principal Component Analysis (PCA) (25) was performed in Sixpack on k^3-weighted µ-EXAFS data to assist in determining an appropriate number of components for LCF. Target transformation (TT) of standards to yield *SPOIL* values (26) were also obtained using Sixpack with three components determined from PCA. *SPOIL* values were ranked similarly as others have (22, 27). Eigenvalues (28) were determined in Sixpack and plotted in a scree plot. The break in the slope of the scree plot assisted to determine an appropriate number of standards for LCF. Visual appearance of principal components were also examined to assist in determining the number of components sufficient to account for the features present in the µ-EXAFS spectra (29).

F-tests (or Hamilton tests) (30) were carried out on LCF results with different numbers of standards to determine if the addition of a new standard significantly improved the fit (29, 31). F-test values were calculated using a regularized lower incomplete beta function calculator (32). For the F-test, a 95% confidence level was implemented (1). By using this cutoff value, there is <5% probability that improvement in the fit is caused by random variation or noise in the data (29). The number of measurements (rounded down to the nearest whole integer) were calculated by Demeter software and used as parameters in the F-test. This was generally 14 measurements for EXAFS LCF for a k-range of 3 to 10, and 12 measurements for a k-range of 3 to 9. For the XANES region, the F-test was generally not necessary to perform (see discussion for each XANES fit adjacent to each Additional file figure), but when the F-test was necessary, six measurements were estimated via a method similar to that described in ref. (29).

The F-test value of $n+1$ (where n is the number of EXAFS standards used in LCF) is the probability that improvement in the fit with one additional standard is related to fitting noise in the data. For example, for a fit with two standards versus a fit with three standards, an F-test value of 30% means there is a 30% probability that fit improvement is due to noise. In this case,

the fit with three standards was discarded and only two standards were used in the final fit. Any F-test value above 5% indicates an insignificant improvement in the fit. If the value is below 5% then the additional standard was considered necessary to improve the fit.

Adding more standards to a linear combination fit generally reduces the R-factor value of the fit, so the F-test provides quantitative data indicating if fit improvement is statistically significant or not (1, 29). The R-factor in LCF in Demeter is a measure of mean square sum of the misfit at each data point (24). For LCF, all weights for standards were restricted to be between 0 and 1; otherwise, negative values for standards would often result. The total of all components was not restricted to equal one because of the potential of self (over) absorption effects in highly concentrated Ni hotspots. When full µ-EXAFS spectra were unattainable due to glitches in the monochromator, the XANES portion of the spectra were analyzed with LCF with the same set of standards. Importantly, where both XANES and EXAFS data were obtained from the same spot, LCF was carried out only on the EXAFS data and not on XANES data.

Energy calibration was carried out for each standard and sample by calibrating the first inflection point of the derivative of accompanying reference Ni foil spectra to 8333 eV. In general, XANES LCF should be carried out on data sets (that is, samples and standards) that are acquired at the same beamline during the same experimental run at the synchrotron. However, in practice with multi-component and heterogeneous systems such as soils and contaminated sediments, this is often not possible. This is particularly true with µ-XAS data collection. During experimental runs for µ-XAS work, most time is consumed collecting maps, diffraction, and µ-XAS data, leaving little or no time available to collect data for sometimes up to tens of reference standards. Given these limitations, a restricted shift in the E_0 value (maximum 0.5 eV shift) as an additional fitting parameter was included into some of the µ-XANES LCF routines.

Allowing E_0 to shift during LCF increases the degrees of freedom of the fit but can also improve the fit. However, this method adds more uncertainty to the fit and must be applied carefully (27). Absolute calibration, which means calibration at the beamline, of all samples and standards is the preferred method. Absolute calibration was carried out for all samples and standards used here; however, standards were collected at different beamlines. XANES data are sensitive not only to absolute energy calibration but also monochromator resolution. Monochromator resolution is dependent on the crystal used at each beamline; thus, it is best to reacquire all standards to be used in LCF at the same beamline and during the same experimental run at the synchrotron (27). Given these needs and limitations, if all samples and standards are acquired carefully and have absolute calibration (for example, to a metal foil), any necessary E_0 shift should be small. Small is a relative term and can be taken to mean on the order of the energy step per point (27). For all samples and standards used here, all data in the XANES region had energy step sizes of 0.5 eV or less. Thus, restricting any E_0 shifts in LCF to 0.5 eV is reasonable because that is the maximum step size for any spectra used. Because all sample data were acquired at the same beamline, no energy shifts were allowed for the samples. Restricted energy shifts were only allowed for standards. Lastly, because the oxidation state of Ni in all samples and standards is expected to be the same (+2), any energy shifts are also expected to be small.

Any shift in E_0 during µ-XANES LCF was restricted to be less than 0.5 eV (including error bars). This was strictly adhered to so that LCF to can provide meaningful results. LCF results with standards that had E_0 shifts >0.5 eV from their calibrated position were discarded (considered invalid). Additionally, E_0 shifts were only allowed for the µ-XANES LCF when

necessary and not allowed during EXAFS LCF. It is important to report any additional fitting parameter included into LCF; thus, delta E_0 values for XANES LCF are reported.

To carry out LCF, all samples and standards must all share a common E_0. The E_0 value chosen was 8342 eV, which falls on the lower portion of the edge jump. The upper end of the XANES region is close to 8390 eV. There is no sharp boundary between the XANES and EXAFS regions, but values such as 30 eV and 50 eV above E_0 are both commonly used to define the XANES region (29). However, based on XANES fitting, it was determined that the range from -20 eV below to +90 eV and above E_0 (8342 eV) was appropriate. This range was chosen to account for the important indentation feature found at 8400 eV in many of the layered serpentine minerals and phyllosilicates. µ-XANES and µ-EXAFS spectra are the same scans, with the XANES portion being at lower energy and the EXAFS portion at higher energy. This facilitates the translation of peak positions in the first oscillation of the XANES region to their corresponding k-space value in the EXAFS region.

Figure S1 - sample “s10t2” thin section overview of maps

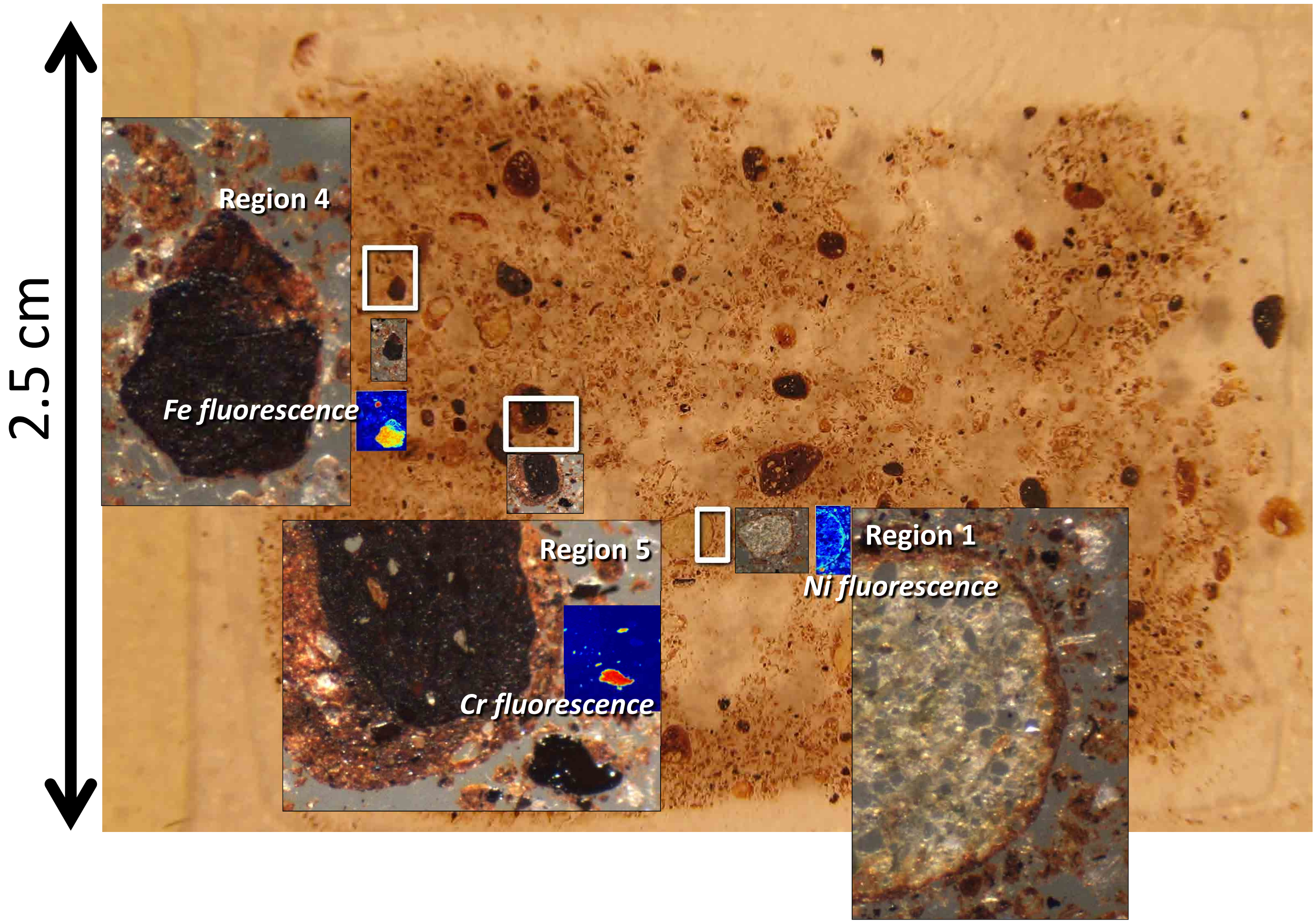

Figure S2 - sample “s11unt” thin section overview of maps

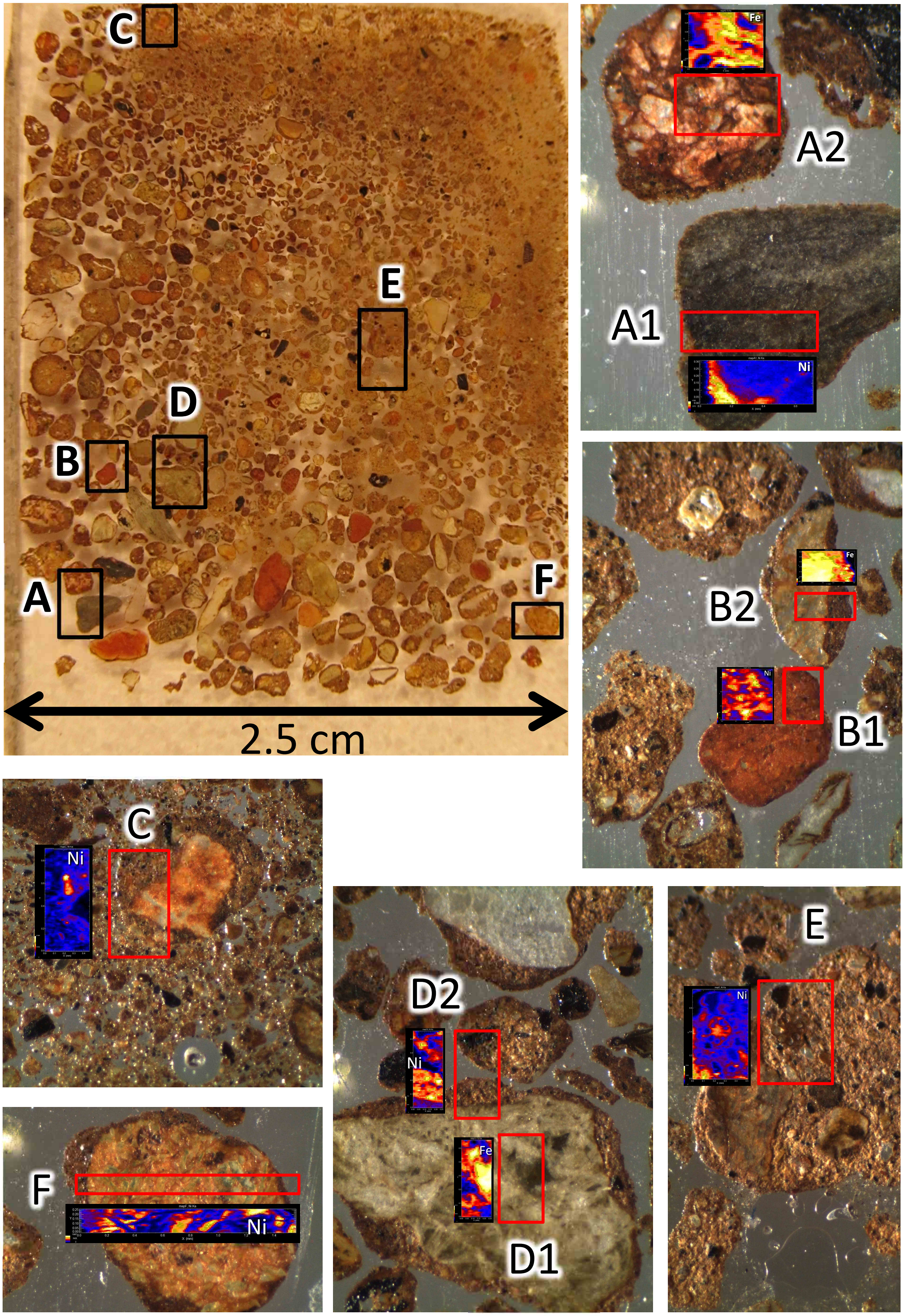

Figure S3 - sample “s20unt” thin section overview of maps

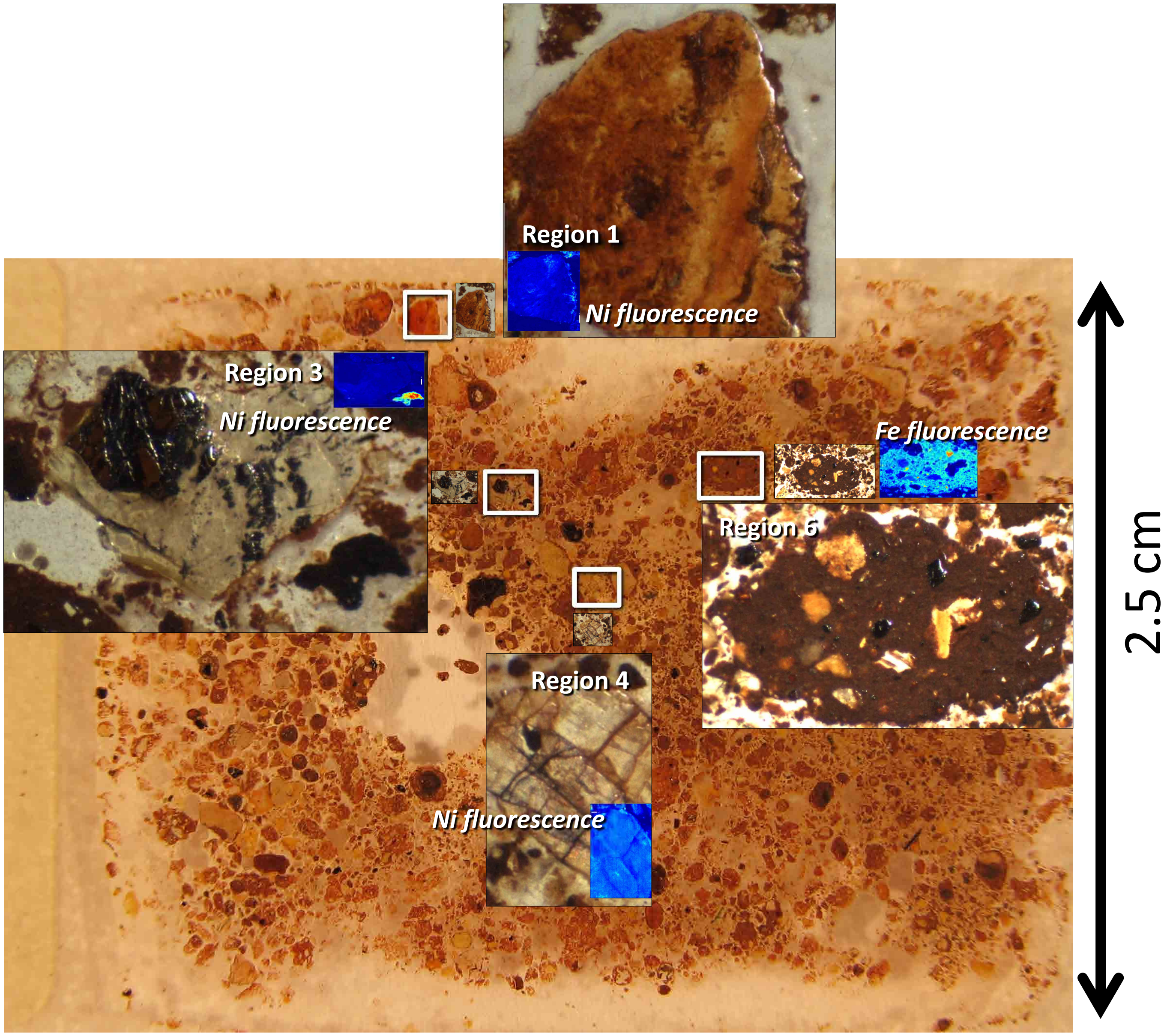

Figure S4a - s10t2 region 1 map

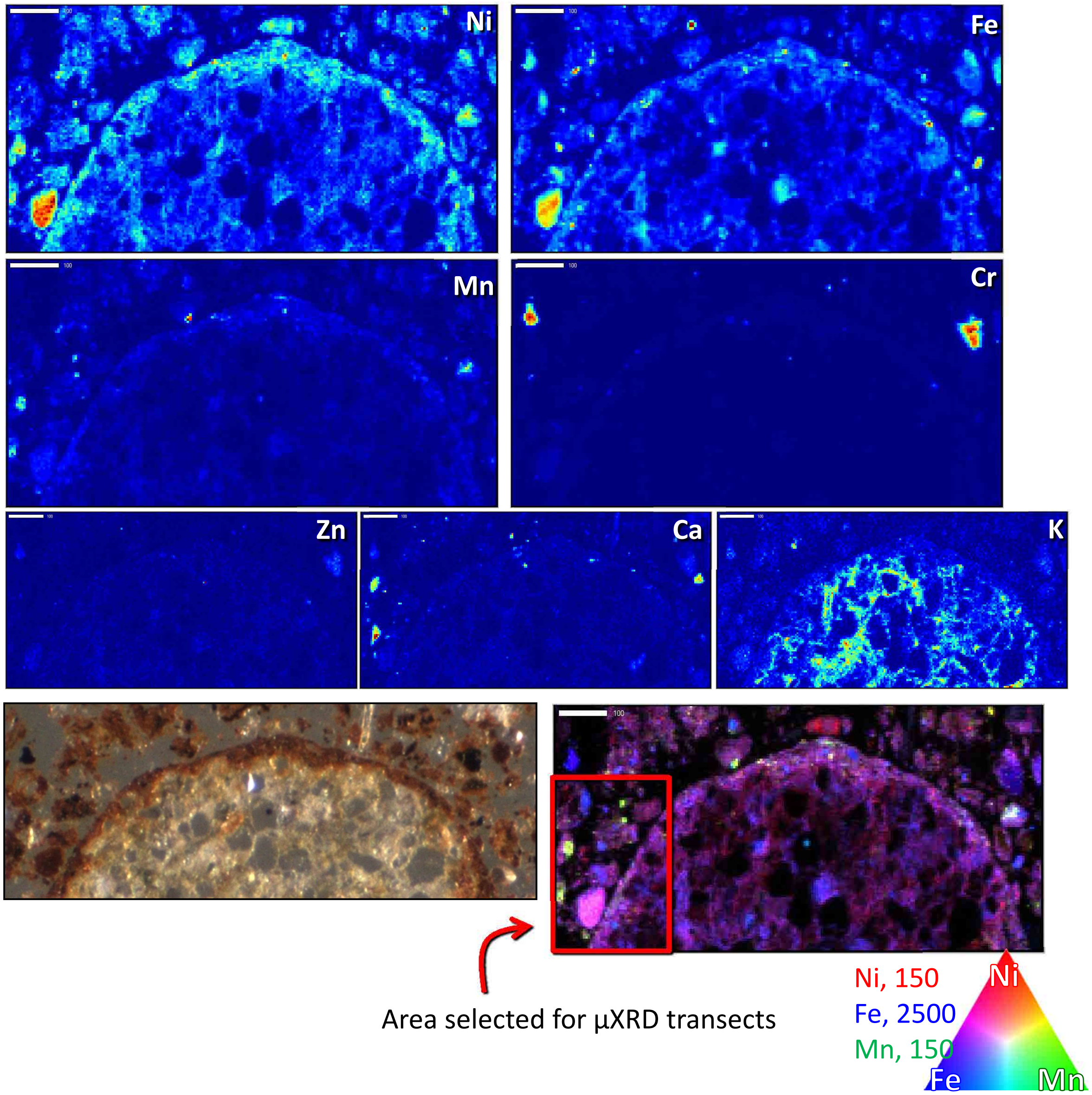

The color scale on the right indicates relative elemental concentration measured in counts per second (CPS). Red is higher concentration (hotter). The highest red values for each elemental map are listed to the right. The lowest blue values (cooler) are always zero. Scale bar is in µm.

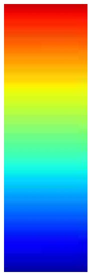

Figure S4b - s10t2 region 1 map (cont.) with μ-XRD

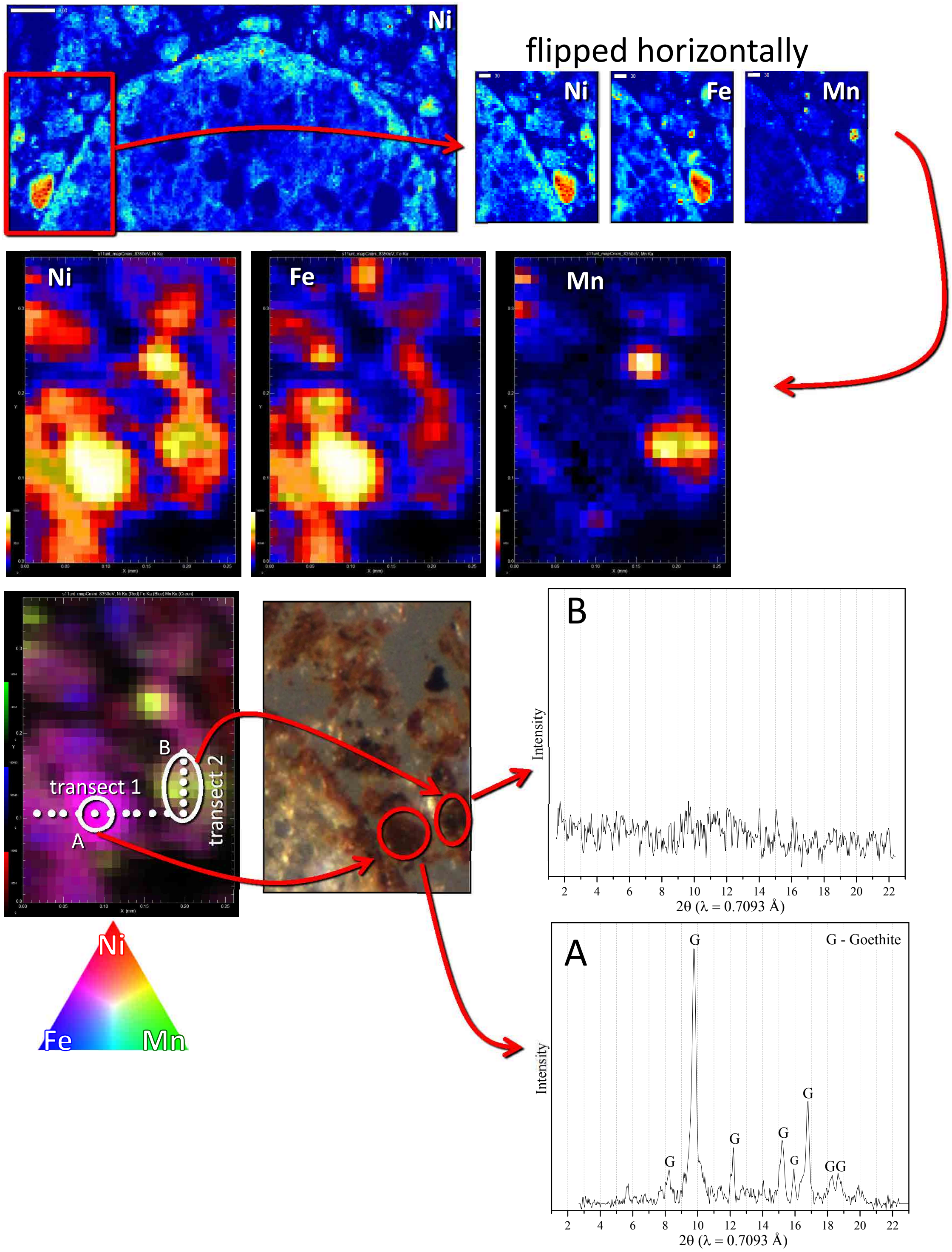

Figures S4a and S4b: “s10t2” region 1

Seven individual elemental maps are presented, including Ni, Fe, Mn, Cr, Zn, Ca, and K. There is higher Ni concentration in the reddish-crust perimeter of the transparent, larger mineral (see photograph inset for visual correlation). The scale bar is 100 µm. A hotspot of Fe and Ni is located in the lower left corner, which matches a deep red colored mineral particle in the photograph. The tricolored map shows a general diffuse correlation between Ni and Fe (purple), though many unassociated Fe hotspots are present. One unassociated Ni hotspot is present in the center upper portion of the tricolored map (red color). Mn hotspots are often correlated with Ni with at least nine occurrences, as illustrated by the greenish-yellow color in the tricolored map.

Generally, Mn is not correlated with Fe except for a single small hotspot in the middle-right side of the map, causing a light blue color in the tricolored map. Two hotspots of Cr are present on the left and right sides of the map. Ni does not associate in a diffuse nor dense manner with Cr, Zn, Ca, or K. Zn is correlated with several of the hotspots of Cr. Ca and K do not appear to correlate either. Maps in Figure S4a were obtained at SSRL. The lower left corner (red box) was re-scanned at NSLS (Figure S4b) to identify Ni incorporation into the solid phase mineral in the Fe/Ni hotspot.

In Figure S4b, the black bordered maps are from NSLS and were used to perform μ-XRD transects. The blue contrasted maps are from SSRL. Using μ-XRD transects, Ni incorporation into the solid phase minerals was examined in Fe/Ni and Mn/Ni hotspots. Note that the NSLS maps are flipped horizontally due to the beamline setup. The elemental distributions (for example, Fe, Mn, and Ni) line up between maps obtained from the two different beamlines at SSRL and NSLS, illustrating the usefulness of re-mapping petrographic thin sections where different beamline capabilities are present. In transect 1 spot “A”, μ-XRD indicates this spot is goethite. Thus Ni accumulates in the structure goethite in this Fe/Ni hotspot. Along transect 2 spot “B”, no diffraction pattern could be obtained from this Ni/Mn hotspot. The lack of a diffraction pattern is a common occurrence at several other Ni/Mn hotspots in other samples (see Table 1 in the main text for a summary of μ-XRD results). Ni distribution in “s10t2” region 1 was found to be heterogeneous on the micrometer scale, being present in both diffuse and dense manners with Fe and in a dense manner with Mn.

Figure S5 - s10t2 region 4 map with µ-XRD

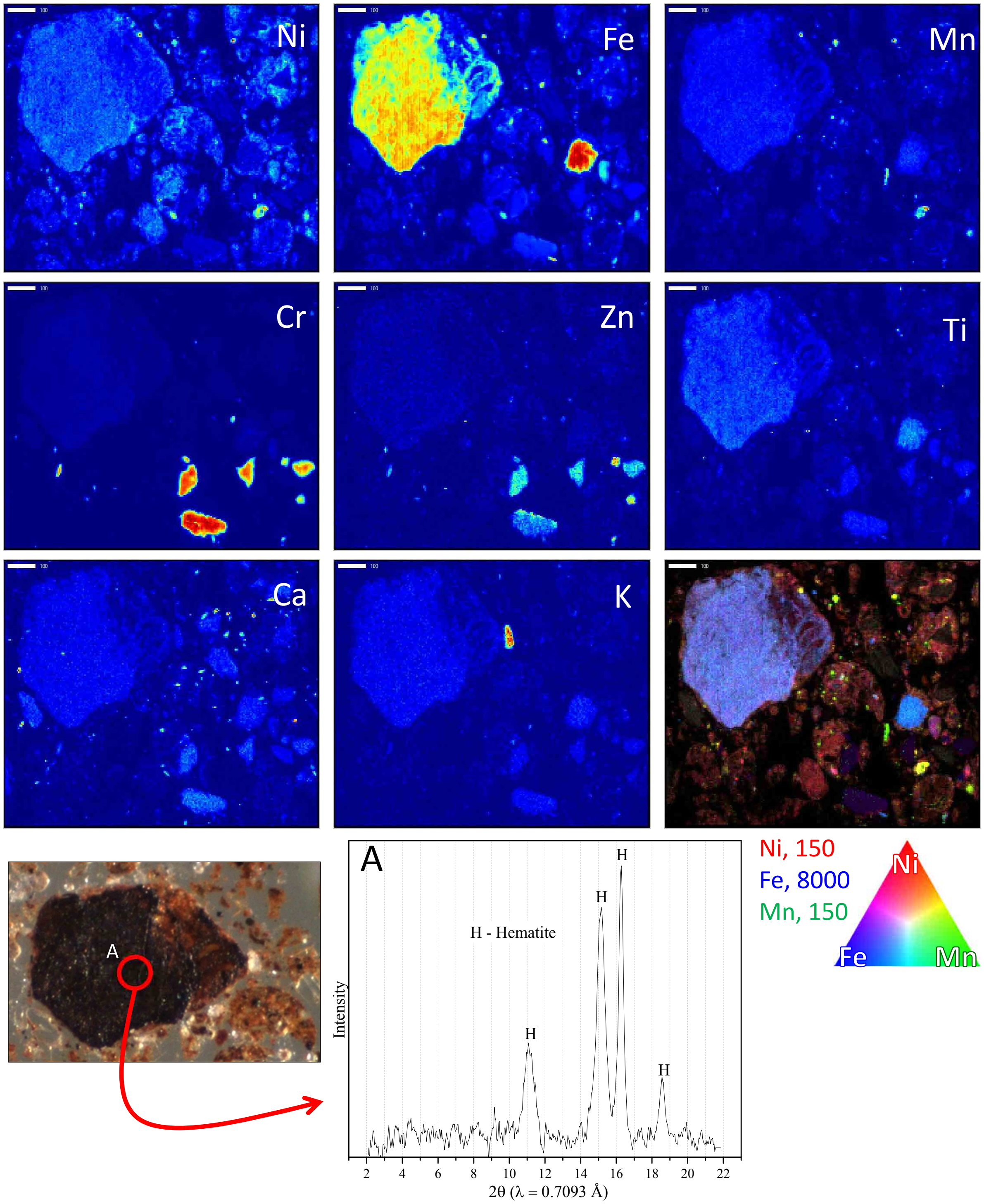

The color scale on the right indicates relative elemental concentration measured in counts per second (CPS). Red is higher concentration (hotter). The highest red values for each elemental map are listed to the right. The lowest blue values (cooler) are always zero. Scale bar is in µm.

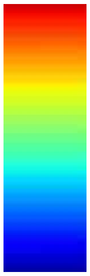

Ni, 300
Fe, 10900
Mn, 500
Cr, 4000
Zn, 100
Ti, 280
Ca, 150
K, 140

Figure S5: “s10t2” region 4

In these maps, Fe is the most concentrated element with over 10000 (counts per second) CPS as compared to 300 and 500 CPS for Ni and Mn, respectively at their highest concentrations. The large black mineral (in photograph) is delineated clearly by Fe fluorescence in the upper left corner of the map. Lower amounts of Ni, Mn, and Ti are also present throughout this mineral. However, the most concentrated spot of Fe is essentially unassociated with other elements (right side). Four Cr-rich minerals about 100 μm in size are present and associated with Zn. This mineral is most likely chromite, as chromite is a common mineral in serpentine soils (33). There are several hotspots where Mn and Ni are present in a dense fashion with the largest spot being about 50 μm in size (yellow color in lower right portion of tricolored map). Microfocused-XRD (inset “A”) on the black, Fe-rich mineral (red circle) indicated this mineral is hematite (Table 1). Ni is present in the hematite at low levels, and the most concentrated Ni spots are associated with Mn.

Figure S6a - s10t2 region 5 map

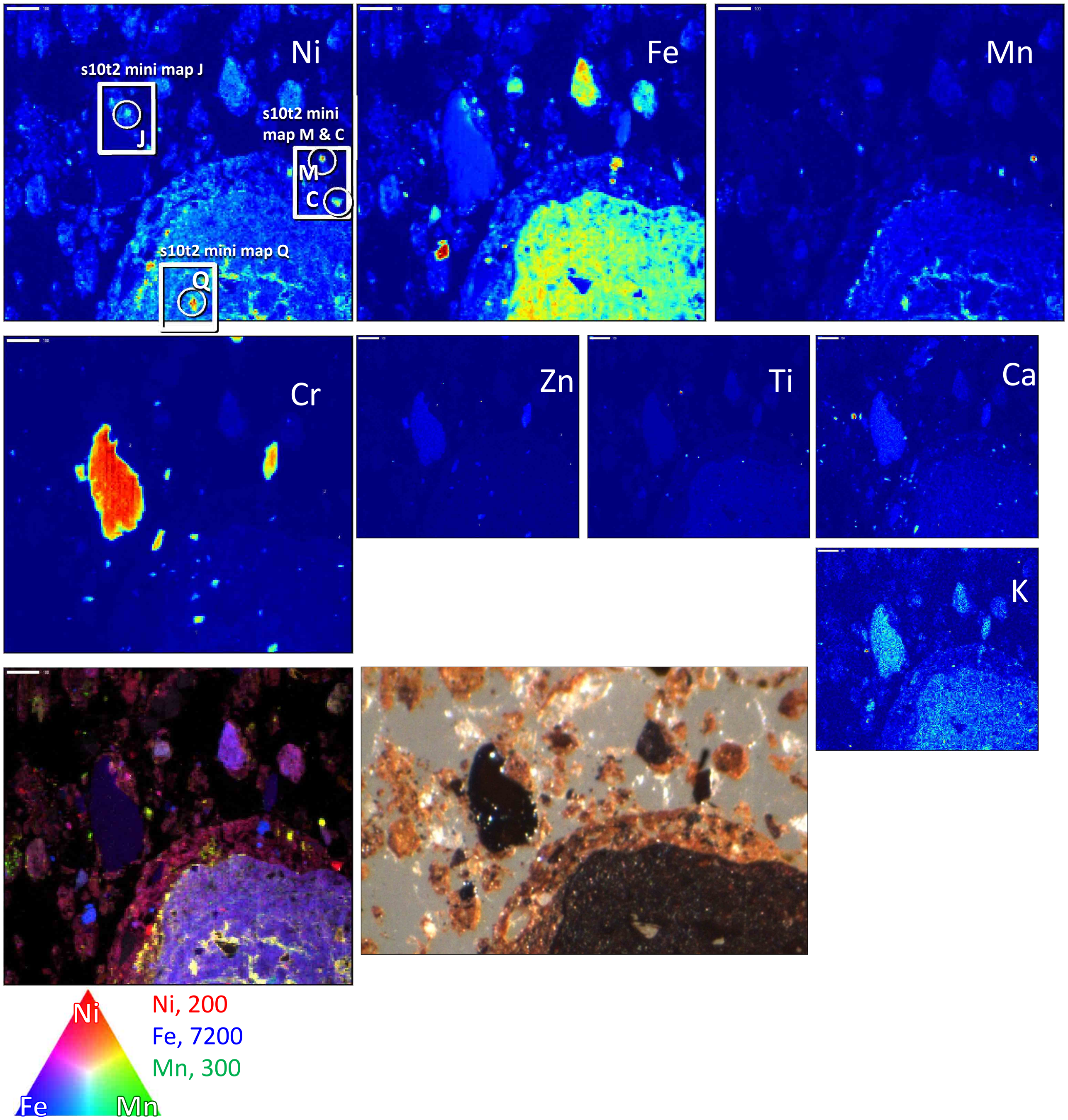

The color scale on the right indicates relative elemental concentration measured in counts per second (CPS). Red is higher concentration (hotter). The highest red values for each elemental map are listed to the right. The lowest blue values (cooler) are always zero. Scale bar is in µm.

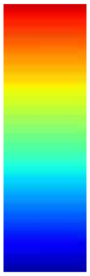

Ni, 390
Fe, 9800
Mn, 890
Cr, 4500
Zn, 470
Ti, 1150
Ca, 250
K, 50

Figure S6a: “s10t2” region 5

This is a large map (the scale bar is 100 µm) with three smaller, higher resolution maps defined (mini maps “J”, “M&C”, “Q”) inside it with white boxes. Those mini maps are respectively Figures S6b, S6c, and S6d, and they allow for precise identification Ni species using microfocused techniques such as µ-XRD and µ-XAS. There are multiple hotspots of Ni, Fe, Mn, and Cr in this map, illustrating the heterogeneity of the sample at the micron scale. Mini “map J” focuses on a heterogeneous area containing hotspots with Cr, Ni, and Fe. Mini map “M&C” focuses on an area with a hotspot of Ni and Mn densely associated and another hotspot of unassociated (isolated) Ni. Mini “map Q” covers an area in the blackish mineral in the lower right. This blackish mineral is primarily Fe-rich, as indicated in the µ-XRF map. However, several intraparticle hotspots where Mn and Ni are densely associated are present (yellow color in tricolor map). Overall, Cr is generally not associated with other elements. Generally, Mn is associated with Ni but there is a disperse area of speckled green color on the left side, indicating unassociated Mn is present in a diffuse manner as well.

Figure S6b - s10t2 region 5 mini map J with µ-XRD & µ-XANES

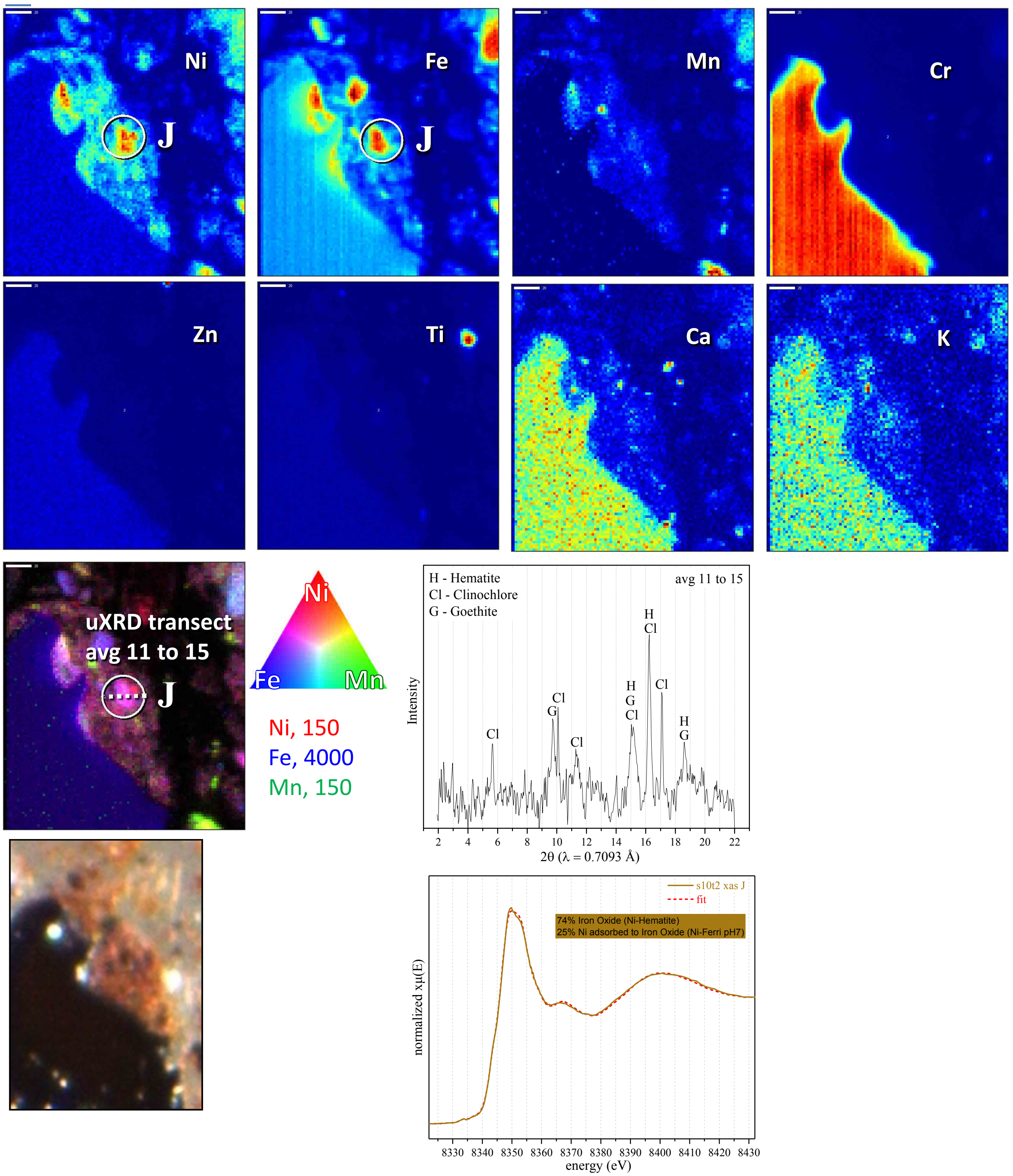

The color scale on the right indicates relative elemental concentration measured in counts per second (CPS). Red is higher concentration (hotter). The highest red values for each elemental map are listed to the right. The lowest blue values (cooler) are always zero. Scale bar is in µm.

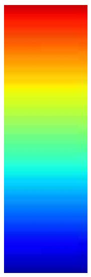

Figure S6b: “s10t2” region 5 mini map J

Spot “J” is the first of five spots where both μ-XRD and μ-XAS techniques were carried out (Table 3). The clear outline of a chromite particle is evident with the higher degree of spatial resolution in this map (scale bar is 20 μm) than in Figure S6a. The top part of this chromite particle looks like a left hand. Spot “J” was selected for μ-XRD and μ-XAS because of the association of Ni and Fe in an agglomerated yet diffuse fashion (versus that of a discrete particle or large mineral surface as seen in Figures S4b and S5). The diffraction data (“avg 11 to 15”) indicate that hematite, clinochlore, and goethite are present in this Ni and Fe hotspot (Table 1, tricolor map). In this heterogeneous spot “J”, Ni is associated with the Fe oxides goethite and hematite, and possible clinochlore if there is heavy substitution of Mg for Fe. This heavy substitution of Mg for Fe would decrease any shoulder formation in the μ-XANES spectra at 8400 eV (see main text for further discussion on this shoulder formation). Calcium and K appear to correlate with Cr but their CPS are very low. At the bottom right, there is a hotspot where Ni and Mn are densely correlated.

In addition to μ-XRD identification of Ni hosts, μ-EXAFS were attempted at this spot; however, because of glitches (for example, at 8500 eV in Figure 2a) in the monochromator, only the XANES region could be analyzed. The oscillation peak at 8400 eV is not indented (no shoulder is present) (Table 3). A split in this oscillation (at this particular energy) is evidence for Ni incorporation into the octahedral sheet of a layered silicate mineral, such as a phyllosilicate like clinochlore. However, this indentation would only occur if the surrounding atoms in the octahedral layer were lighter in weight, such as Mg or Al (1, 13, 14). Because ultramafic parent material is high in Mg, Mg would likely be the dominant light-weight cation (not Al). The concentration of Mg in this soil is 15700 mg kg^{-1} (Table S1). This statement is also supported by EXAFS spectra of “Ni-rich” and “Ni-poor” serpentine minerals (34), the former which lack an indentation in the first oscillation and the latter which displays an indentation similar to the serpentine mineral standards in Figure S24. To identify with which phase Ni was associated, LCF was carried out on the μ-XANES data.

Because Ni and Fe were the dominant elements to fluoresce at spot “J” and because the μ-XRD data indicated the presence of goethite and hematite, those particular standards were expected to be important in the LCF of Ni for this spot. There were low counts of Mn in the μ-XRD transect. Two standards fit this spectrum (74% Ni-Hematite and 25% Ni-Ferri pH7), which indicate that at this spot the Fe-oxides dominate in terms of their association with Ni. When an additional standard was added to the fit, the decrease in the R-factor was large however not significant to necessitate the use of anymore standards. This was determined by the F-test, which yielded a 5.4% chance that fit improvement was due to noise, which is above the 5% cutoff limit. Results from LCF were in agreement with the μ-XRD data; both indicated that iron oxides play an important role at this spot, which illustrates the complementary nature of these two methods.

Figure S6c - s10t2 region 5 mini map M&C with µ-XANES

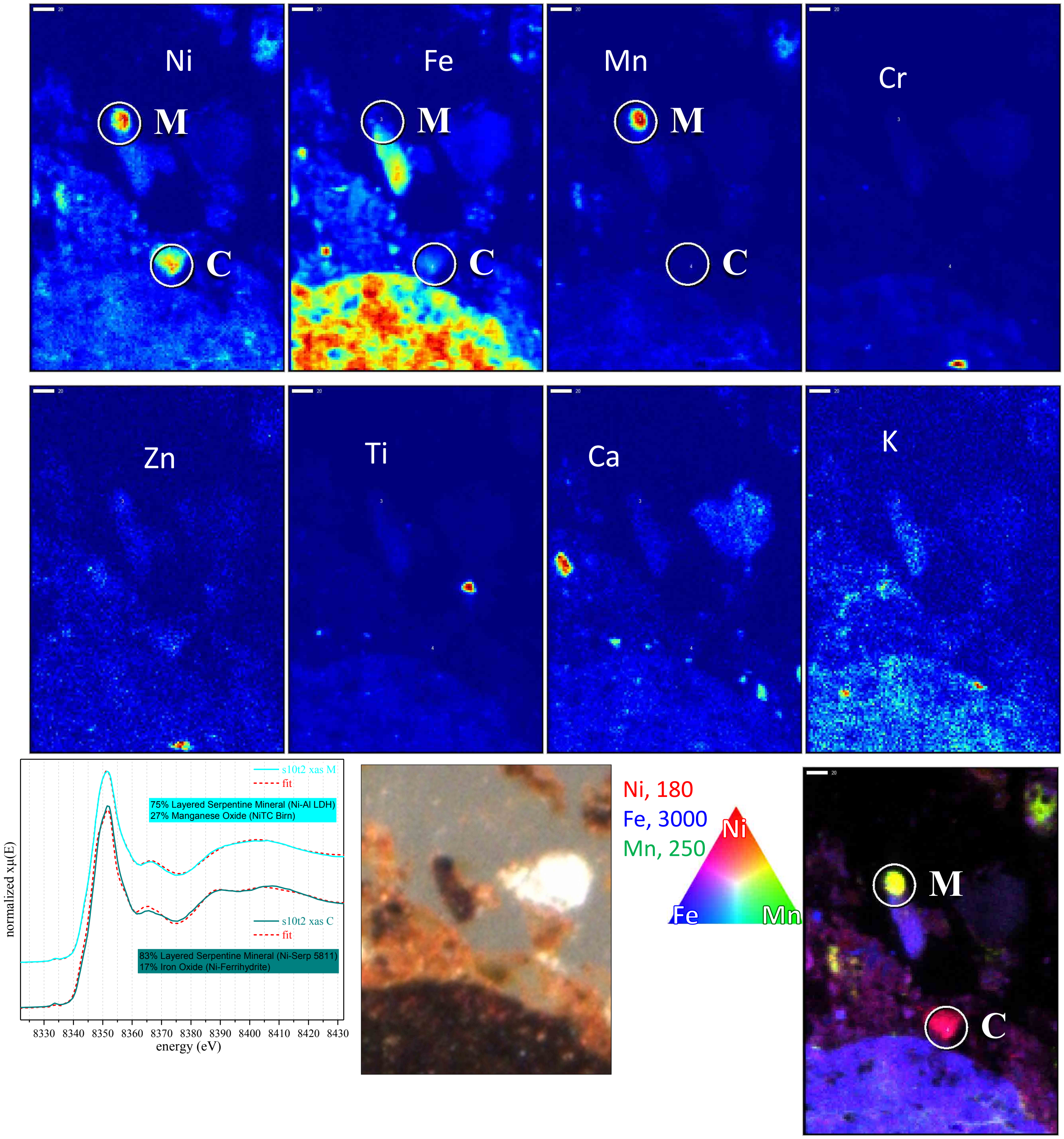

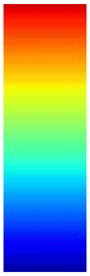

The color scale on the right indicates relative elemental concentration measured in counts per second (CPS). Red is higher concentration (hotter). The highest red values for each elemental map are listed to the right. The lowest blue values (cooler) are always zero. Scale bar is in µm.

Ni, 340
Fe, 5490
Mn, 840
Cr, 2070
Zn, 40
Ti, 590
Ca, 110
K, 40

Figure S6c: “s10t2” region 5 mini map M&C

This high-resolution map was taken to further examine the two discrete and distinct spots labeled “M” and “C”. Both spots are well defined and not diffuse in morphology. Apart from these two Ni hotspots, the black mineral at the bottom is high in Fe. There are several isolated spots of Ti and Ca, and Zn is associated with a Cr hotspot. μ-XANES was carried out on both spots and distinct features are present in the XANES data. Only the XANES region is presented because of sever glitches (for example, at 8500 eV, Figure 2) in the monochromator. At spot “M”, Ni and Mn are densely associated. No indentation or shoulder is present at 8400 eV. The lack of a shoulder feature is probably because Ni is surrounded by Mn, as indicated by their association in the tricolor map (see main text Section 3.2.3 for further discussion). For “xas M” the E_0 value was allowed a restricted shift during the fit (Text S2.4). When $E_0$was not shifted, the F-test indicated the use of three standards did not reduce the R-factor, so only two standards were considered for this fit. The final fit yielded NiAl-LDH (75%) and Ni sorbed to triclinic birnessite (NiTC Birn 27%). This result does not mean that NiAl-LDH is the precise species in the sample; rather, the NiAl-LDH standard means that Ni is in the octahedral sheet of a layered mineral, such as lizardite or a chlorite-group mineral. A discussion on how the NiAl-LDH standard is a good analogue for Ni incorporated into the octahedral layer of a serpentine mineral is presented in Text S2.3 and Figure S16a/b.

At spot “C”, Ni is unassociated with other elements (red in tricolored map), except for low CPS of Fe. For spot “C”, an indentation in the peak at 8400 eV indicates that Ni is present in an octahedral layer and surrounded by light atoms, such as Mg (1). Because of the low CPS of Mn at spot xas C, the fits were restricted to remove all manganese oxide standards. Without shifting E_0, two fits each with two standards produced comparable results. One result from the LCF found that spot “xas C” could be reconstructed using Ni-Ferrihydrite (17%) and Ni-Serp5811 (83%), and the other result found that NiAl LDH (34%) and Ni-Serp5811 (66%). Again, this result does not mean that NiAl-LDH is the precise species in the sample; rather, a discussion on how the NiAl-LDH standard is a good analogue for Ni incorporated into the octahedral layer of a serpentine mineral is presented in Text S2.3 and Figure S16a/b. Thus the results of LCF agree well that the majority of Ni in this spot is bound into a layered octahedral sheet. Shifting E_0 during the fit did not produce better results. When $E_0$was not shifted, the F-test indicated the use of three standards did not reduce the R-factor.

Figure S6d - s10t2 region 5 mini map Q with µ-XANES

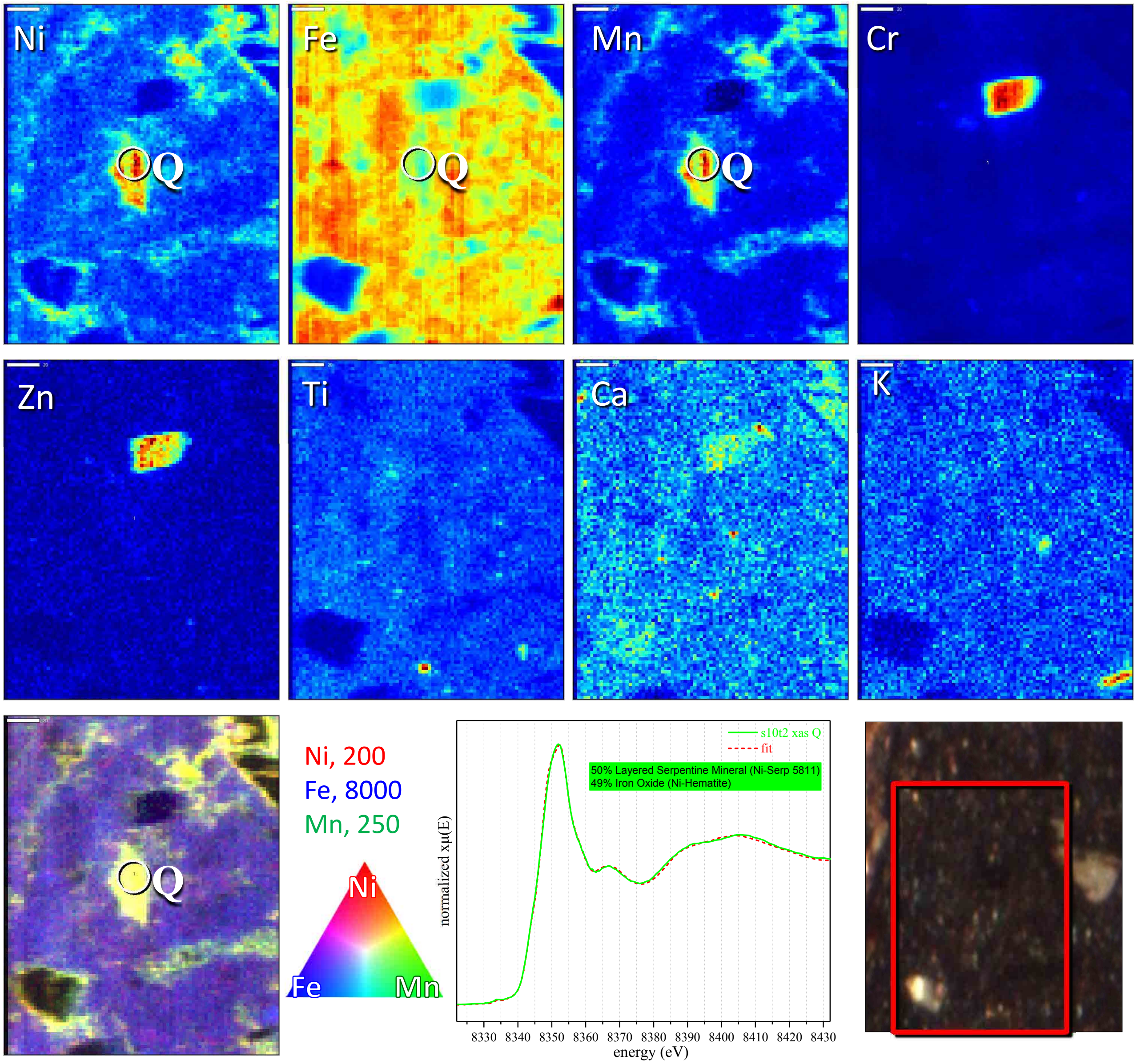

The color scale on the right indicates relative elemental concentration measured in counts per second (CPS). Red is higher concentration (hotter). The highest red values for each elemental map are listed to the right. The lowest blue values (cooler) are always zero. Scale bar is in µm.

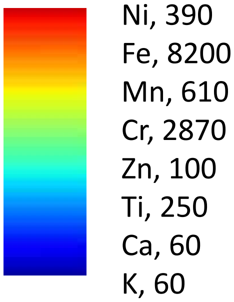

Figure S6d: “s10t2” region 5 mini map Q

This high-resolution map was taken because it is located in the interior of an Fe-rich mineral, but there are distinct associations of Ni with Mn within the Fe-rich mineral. For example, there are both diffuse and discrete areas where Ni and Mn associate in this map. However, at spot “Q”, the counts of Fe decrease to cooler colors (orange to blue), indicating a decrease in Ni associated with Fe at this spot, and Mn counts increase and correlate well with Ni (yellow in the tricolor map). Glitches (for example, at 8500 eV in Figure 2) prevented acquisition of a µ-EXAFS spectrum, but the µ-XANES data show a distinct yet subtle shoulder at 8400 eV. This feature is not present in the hotspot of Ni and Mn at spot M in the previous figure (Figure S6c, Table 3). Although Ni is associated with Mn at both spots (spot “Q” and spot “M”), there are differences in the local atomic environment of Ni. The shoulder present in the spectrum at spot “Q” indicates an increased presence of a lighter element (for example, Mg) in the local atomic environment of Ni, particularly when Ni is located in an octahedral layer (1).

Because of the high CPS of Mn found at this spot, it was suspected that manganese oxides would play in an important role in Ni speciation. However, when the fits were forced to use the any of the manganese oxide standards, the calculated percentages were zero for all three standards (see Table S2 and Figure S24 for all a description and figure of all standards). Because manganese oxides do not fit in this spectrum, Mn will be incorporated into another mineral, such as a layered phyllosilicate. This agrees with the shoulder feature present in “xas Q” because the manganese oxide standards do not have this feature. Additionally, the white lines for the manganese oxide standards overlap poorly with the “xas Q” spectrum. The peak of the white line for the manganese oxide spectra is at 8350 eV while the white line peak for “xas Q” is at 8352 eV (see plots of XANES standards in Figures S24). The white line is the portion of the spectrum that is sharp and at the top of the absorption edge (29) . This small difference in energy (2 eV) is indicative of how LCF of XANES data is highly sensitive to absolute calibration of samples and standards. Meticulous and consistent calibration of samples and standards needs to be carried out prior to embarking on any LCF endeavor.

The best LCF results indicated that the “xas Q” spectrum could be reconstructed with two standards (50% Ni-Serp 5811 and 49% Ni-Hematite). Given that the adjacent surroundings of “xas Q” are high in Fe CPS, this would indicate that a serpentine mineral that is also rich in Mn has become imbedded into an iron oxide. The color from the photograph shows the bulk mineral around this spot to be black in color, similar Figure S5 where hematite was identified by µ-XRD. Given the consistently high CPS and similar morphology of Fe fluorescence in both Figure S5 and S6d, it could be suggested that serpentine minerals (for example, lizardite) can become embedded within larger iron oxide particles (for example, hematite). An F-test was not necessary for “xas Q” because even though fits could be obtained with improved R-factor values, those fits used standards with E_0 shift values more than 0.5 eV. Any fits with E_0 shifts >0.5 eV were discarded.

Figure S7 - s10t2 clay particles map with µ-XRD

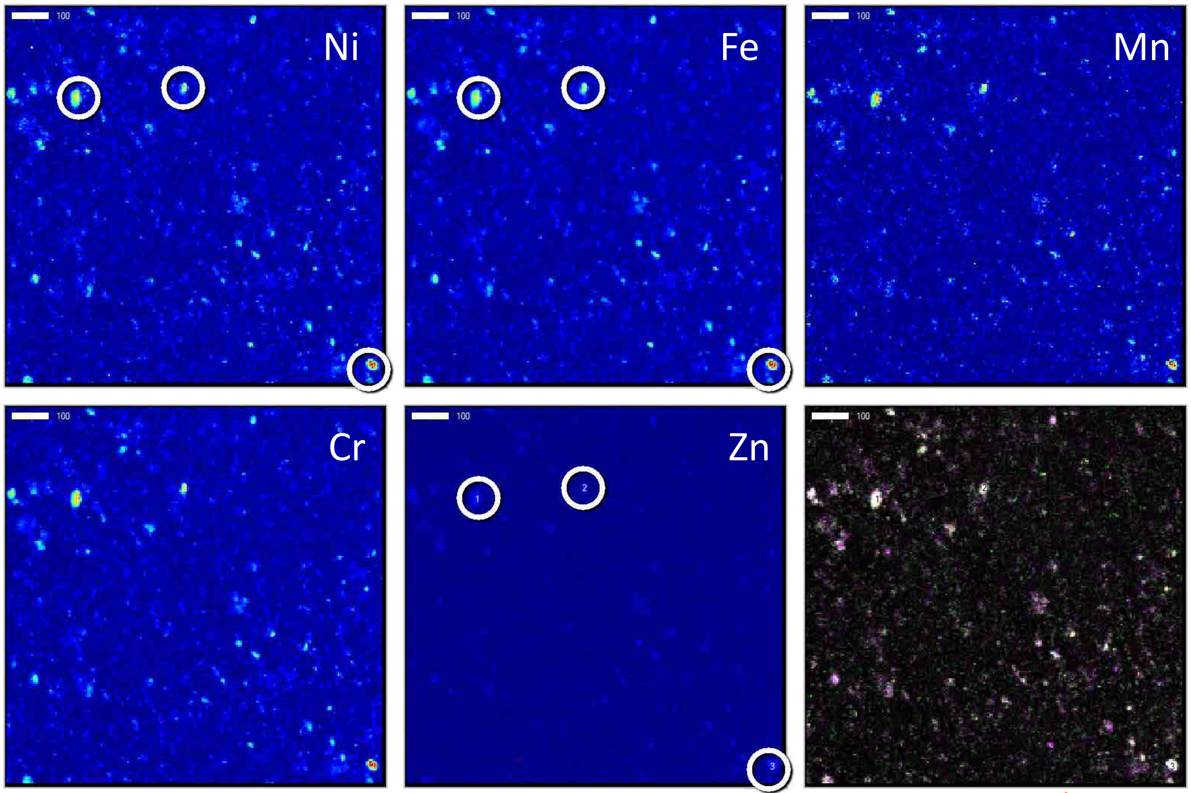

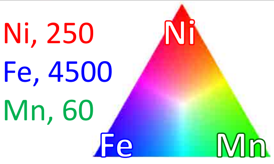

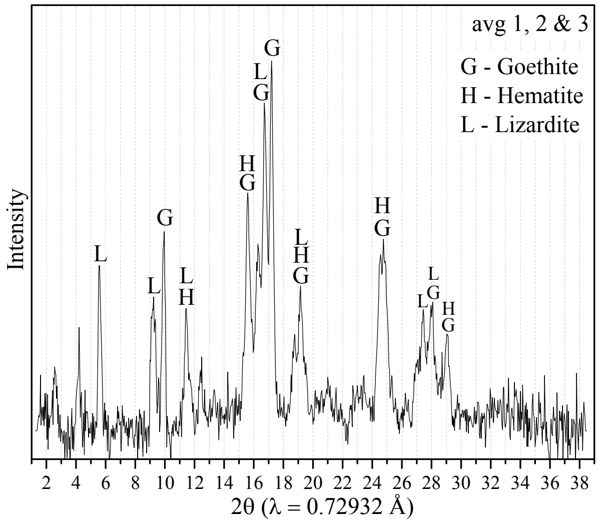

The color scale on the right indicates relative elemental concentration measured in counts per second (CPS). Red is the higher concentration. The highest red values for each map are below. The lowest blue values are always zero. Scale bar is in µm.

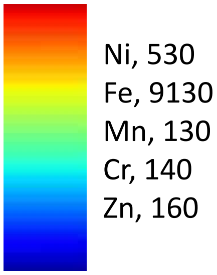

Figure S7: “s10t2” clay particles

These particles were obtained via a sonication and centrifugation method (1). Clay size particles by definition are ≤ 2 μm in diameter. The beam size at SSRL BL2-3 is 2 μm. Thus elements are heavily associated in this map because particles are smaller than the beam size. Ni and Cr, which essentially never associate with each other in all other maps, are correlated here only because all particles are smaller than the X-ray beam. Three spots where clay particles agglomerated together were chosen for μ-XRD. These spots yielded similar diffraction patterns that were then averaged together, “avg 1, 2 & 3”. The peaks were identified as goethite, hematite, and lizardite (Table 1). These minerals were also present in the bulk XRD of the “s10t2” clay fraction (1). The tricolor plot shows white for these three areas because Ni, Fe and Mn are all present. No resolution of discrete particles is possible from the clay size fraction because the clay particles are smaller than the X-ray beam.

Figure S8a - s10t2 silt particles map

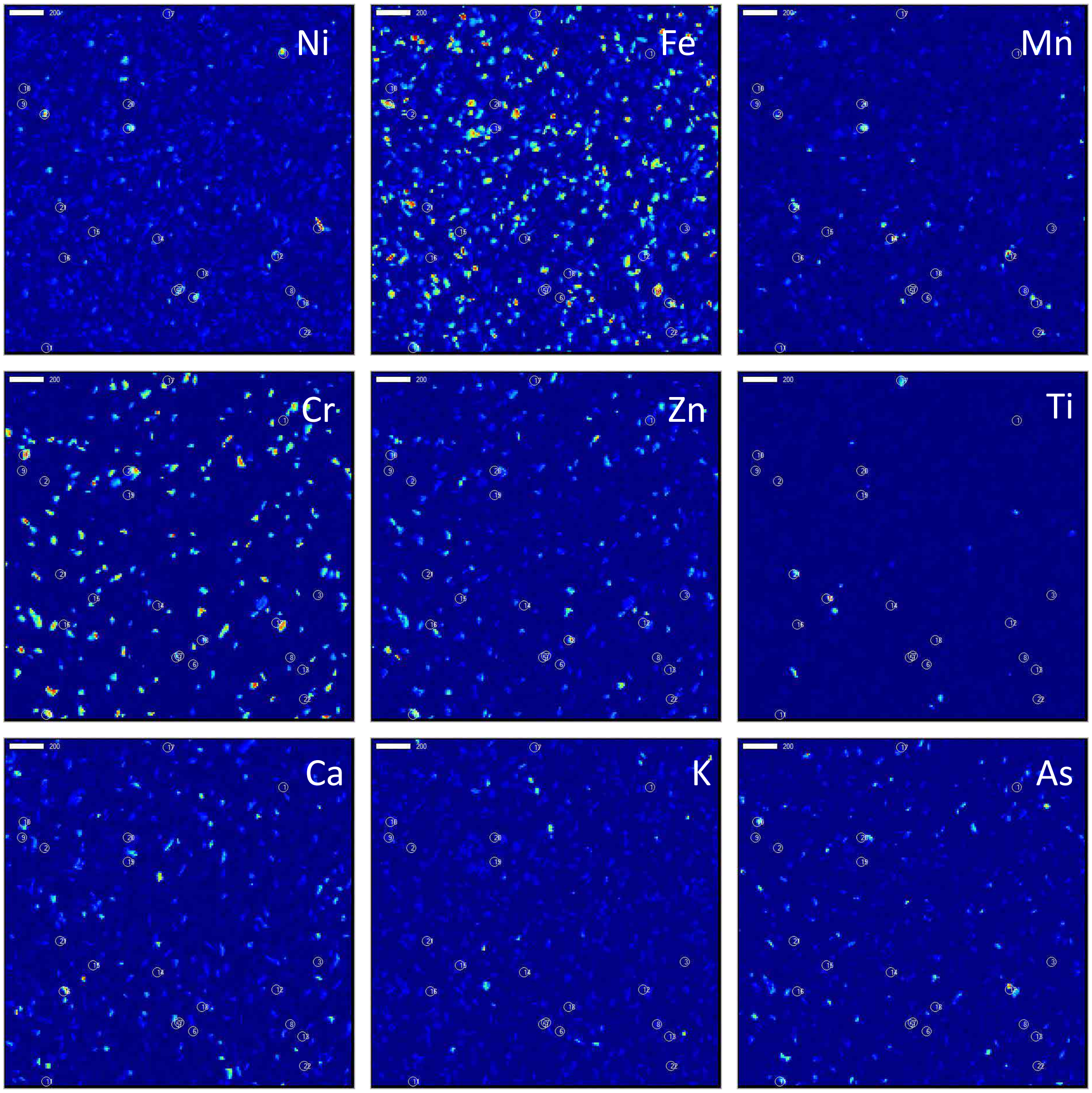

The color scale on the right indicates relative elemental concentration measured in counts per second (CPS). Red is the higher concentration. The highest red values for each map are below. The lowest blue values are always zero. Scale bar is in µm.

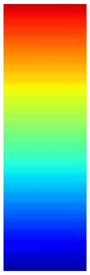

Ni, 2320
Fe, 28350
Mn, 2380
Cr, 13560
Zn, 500
Ti, 7190
Ca, 1860
K, 480
As, 220

Figure S8b - s10t2 silt particles map (cont.) with µ-XRD

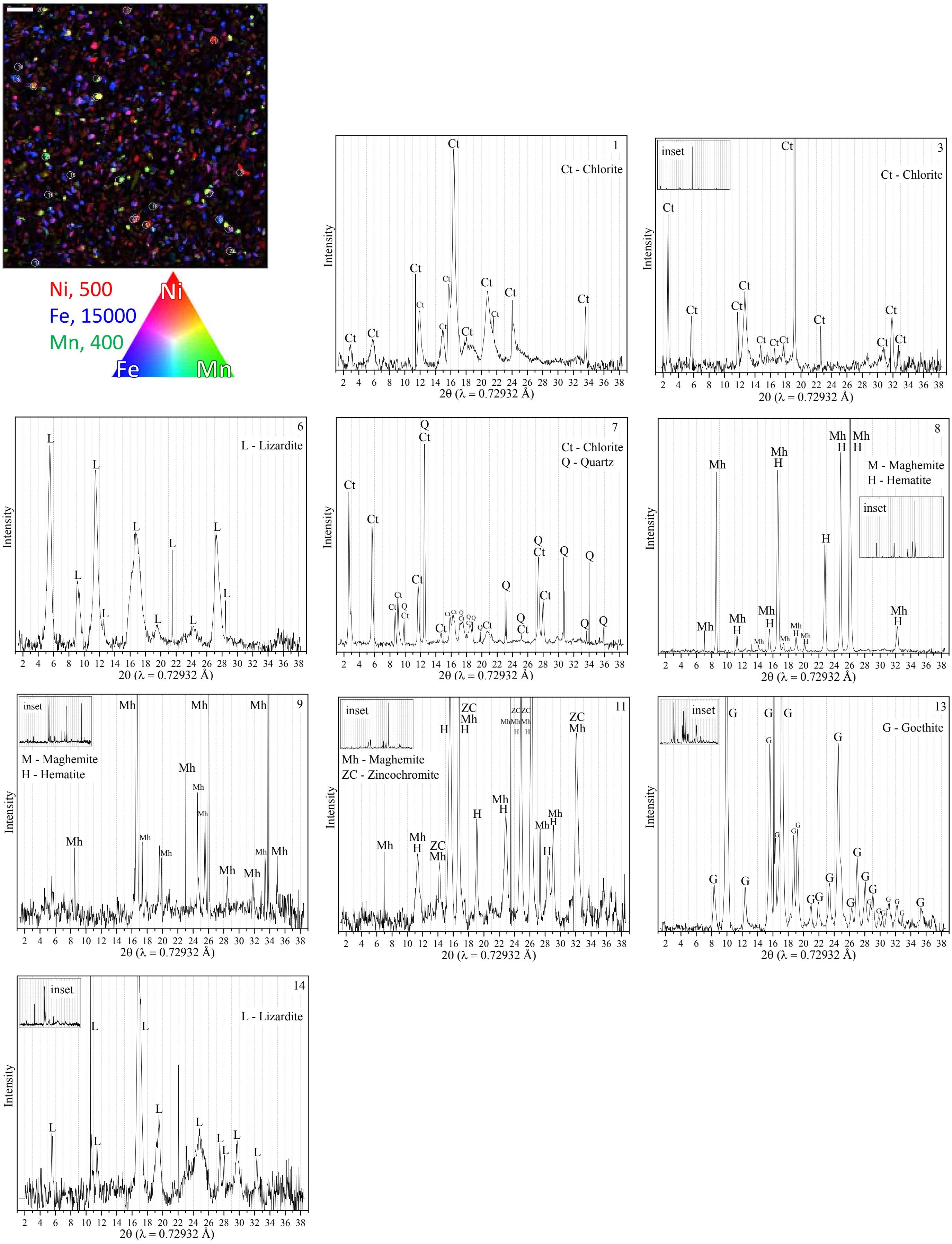

Figures S8a and S8b: “s10t2” silt particles

By looking at the CPS for each element, a comparison of elements with similar Z can qualitatively describe that elements with the hottest spots have the highest concentrations. Fe has the hottest spots (over 28000 CPS). Cr fluorescence is high at over 13500 CPS, likely from chromite particles. Note the scale bar is 200 µm, and the silt fraction is 25–45 µm. Thus, this particle size is close to the lower limit of reasonable data acquisition for this particular large map. A smaller scaled map would have provided better resolution of the particles (for example, see Figure S19c). Individual spots of interest based on elemental associations were selected and labeled with numbers and circles, which are the same for all florescence maps in Figures S8a and S8b. Microfocused-XRD was carried out on these spots. Additionally in Figure S8b, a tricolor map with a combination of Ni, Fe, and Mn fluorescence is shown.

Figure S8b is the continuation of data from Figure S8a. The tricolor plot is shown along with nine µ-XRD patterns. Of the spots labeled on the map, these diffraction patterns had identifiable peaks (nine spectra in all are shown and labeled “1”, “3”, “6”, “7”, “8”, “9”, “11”, “13”, and “14”). Table 1 gives the minerals found via µ-XRD along with the elemental associations at that spot. Spots “1”, “3”, “6”, and “7” are all Ni-rich and not correlated with Fe or Mn, and they contain layered phyllosilicate minerals (lizardite and chlorite). Chlorite is commonly found in many of these samples, and several minerals in this group, such as clinochlore and chamosite, share multiple chlorite peaks. The presence of Ni in these phases indicates that these minerals play important roles in Ni speciation.

Spots “8” and “9” are high in Fe and both hematite and maghemite were identified based on their diffraction patterns. Spot “11”, which has Cr, Fe, and Zn, has peaks for chromite/ zincochromite and maghemite, all of which are related spinels. In spot “13”, Ni is present in goethite, again indicating the importance of Fe oxides in Ni speciation. Spot “14” shows fluorescence of unassociated Mn and peaks for lizardite are identified. This indicates that Mn can substitute heavily into lizardite. At multiple other spots in other samples, Ni fluorescence was also detected from lizardite minerals (Table 1).

Figure S9a - s10t2 medium sand particles map

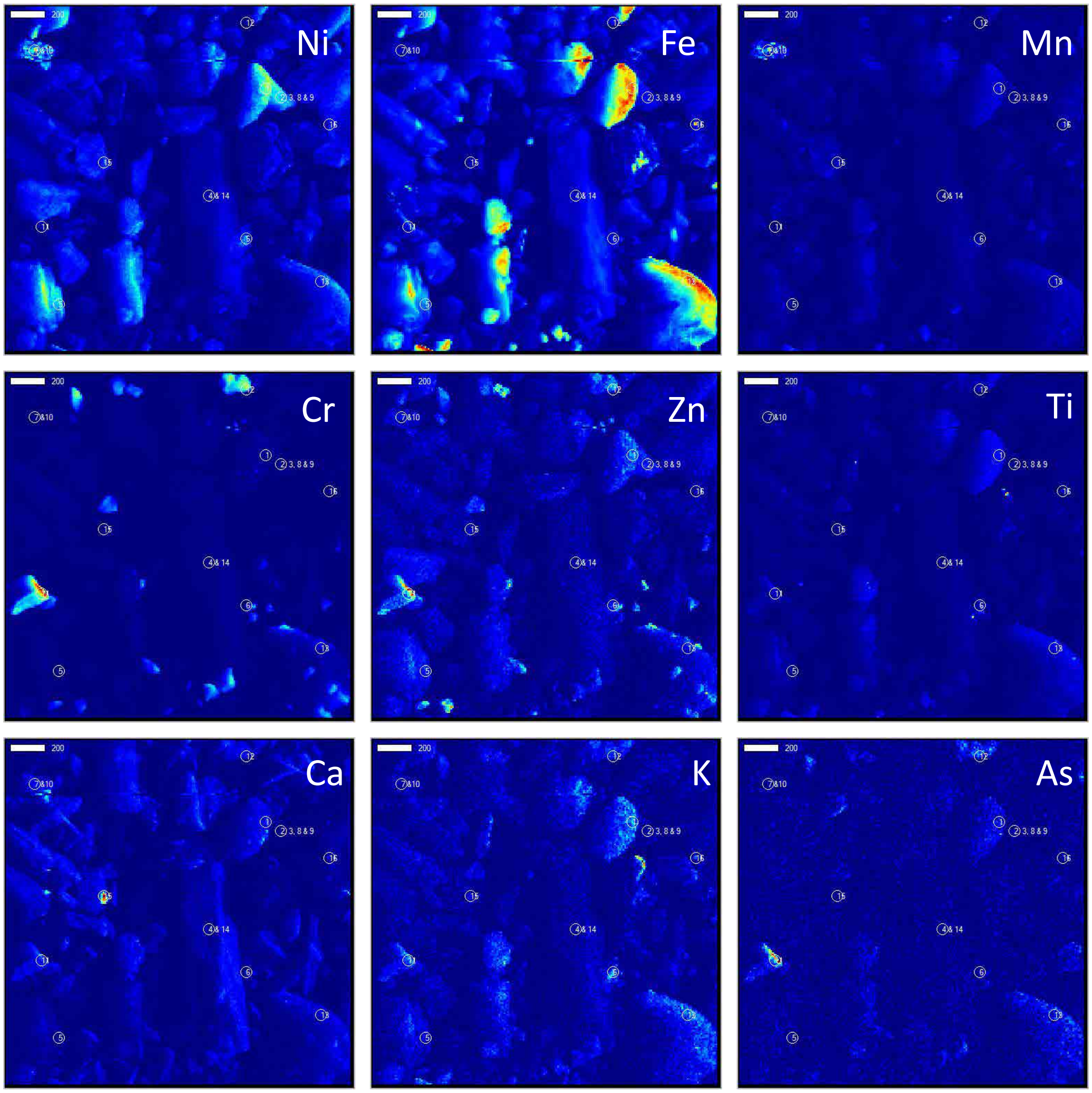

The color scale on the right indicates relative elemental concentration measured in counts per second (CPS). Red is the higher concentration. The highest red values for each map are below. The lowest blue values are always zero. Scale bar is in µm.

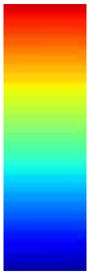

Ni, 760
Fe, 11260
Mn, 1390
Cr, 8160
Zn, 110
Ti, 690
Ca, 270
K, 80
As, 50

Figure S9b - s10t2 medium sand particles map (cont.) with µ-XRD

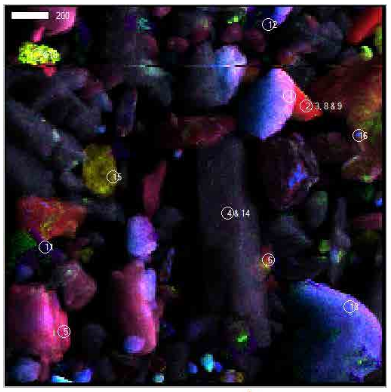

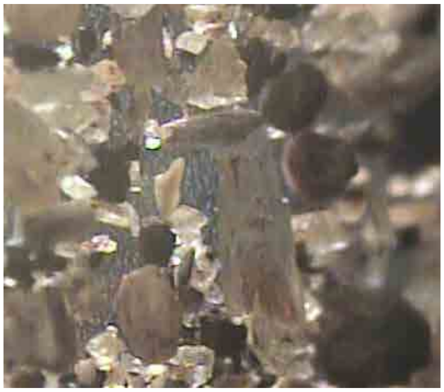

Ni, 300
Fe, 6000
Mn, 200

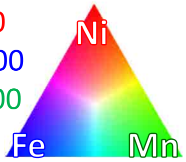

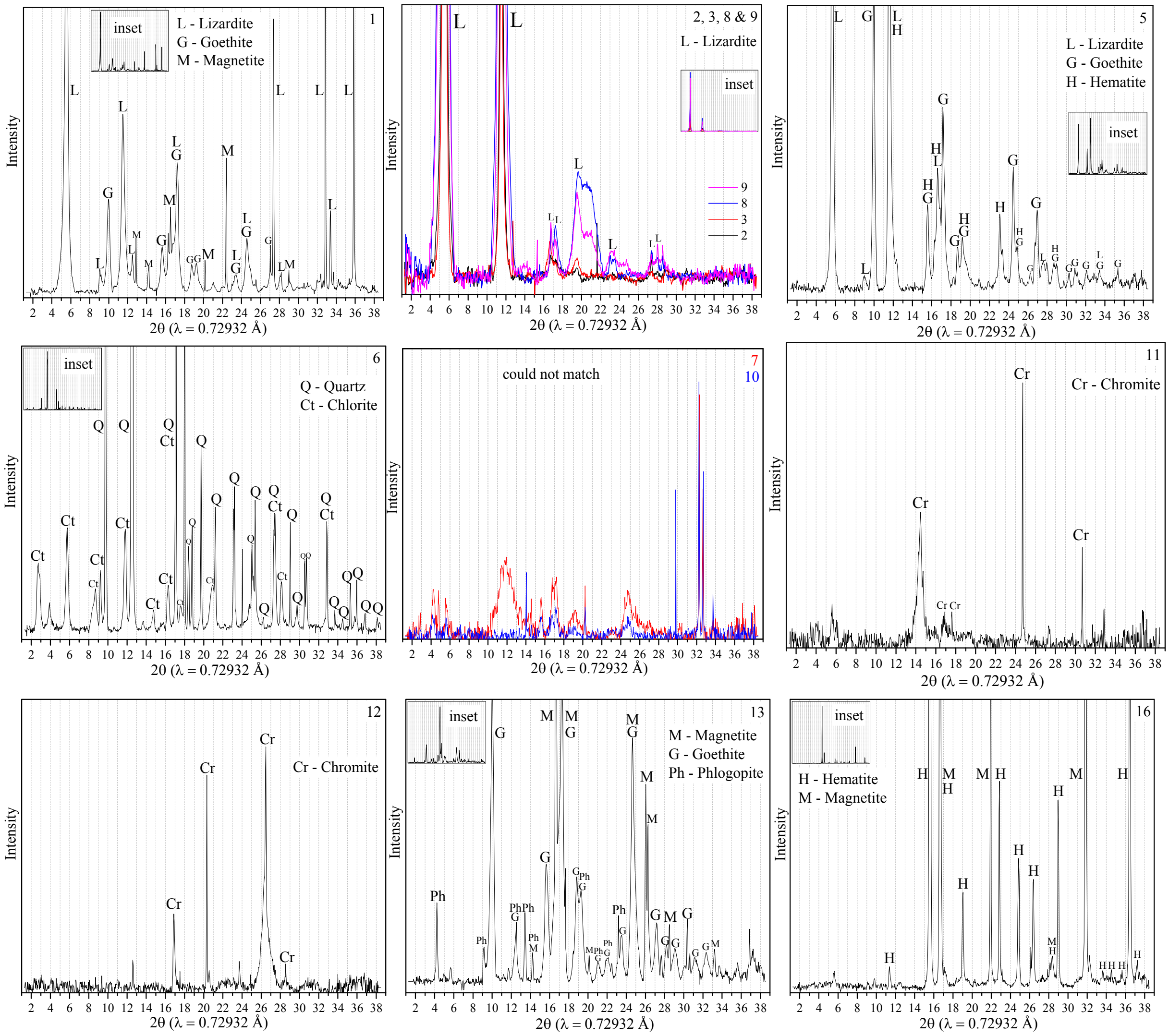

Figures S9a and S9b: “s10t2” medium sand particles

Sand grains are clearly outlined by the fluorescence of different elements. However, there are some artifacts in the fluorescence because the large grain size. For example, the hot Fe mineral in the lower right has the hottest portion on the edge of the mineral closest to the detector. Based on elemental fluorescence of Ni, multiple spots were selected to identify Ni species and other minerals using μ-XRD. These spots are labeled with numbers and circles; their diffractograms are presented in Figure S9b. Spectra “2”, “3”, “8” and “9” were taken from the same mineral, and so were “7” and “10”.

The tricolor map in Figure S9b indicates multiple areas where Ni is unassociated (red areas) with other metals such as Fe and Mn. There are several particles where Ni and Mn associate, particularly in the upper left corner where the greenish-yellow color is present. Purple indicates areas where Ni and Fe are associated. The accompanying photograph of the sample map (taken with the beamline camera while the sample was in place) gives clear indications of the edges of discrete particles which match up with the fluorescence. Spot “1” is a particle where Fe and Ni are associated; peaks for goethite, magnetite and lizardite were identified in the same μ-XRD spectra, indicating these minerals can mix together on the micrometer scale. Spectra “2”, “3”, “8”, and “9” all show diffraction peaks for lizardite. Lizardite is thus a mineral that can also contain high levels of Ni in its octahedral layer. Spot “5” is high in both Ni and Fe, and again peaks for goethite, hematite, and lizardite were identified. Ni is not correlated with other elements in spot “6”; chlorite and quartz were identified in its diffraction pattern.

Because of the high association of Ni and Mn in the large mineral in the top left corner of the tricolor map (yellow color), it was considered a relatively easy and important hotspot to measure. Two diffractograms (“7” and “10”) were taken at different locations on the mineral to try and identify it. Unfortunately, as was the case in several other instances where Ni and Mn were associated (for example, “s10t2” region 1), this mineral provided weak diffraction peaks and could not be positively identified. Although, the broad peaks at ca 12 and 15 °2θ line up with lizardite, there are several sharp peaks between 32 and 34 °2θ that could not be identified. Lack of diffraction peaks is often one of the challenges for analysis of μ-XRD data. Peaks for chromite were identified at spots “11” and “12” where Cr and Zn fluorescence were detected. The multiple peaks in spot “13”, which was high in Fe and low in Ni, were identified as goethite, magnetite, and phlogopite. Phlogopite is in the mica group, which may seem strange to find in ultramafic soils; however, it is the magnesium-rich end member and ultramafic rocks and soils are known for their high Mg concentrations. Phlogopite can be found in ultramafic rocks and serpentinite and can be associated with olivine and magnetite (35). Lastly, one hotspot of Fe at spot “16” show peaks for hematite and magnetite. Figure 9Sb is the last figure involving sample “s10t2”.

Figure S10 - s11unt map A1 with μ-XRD

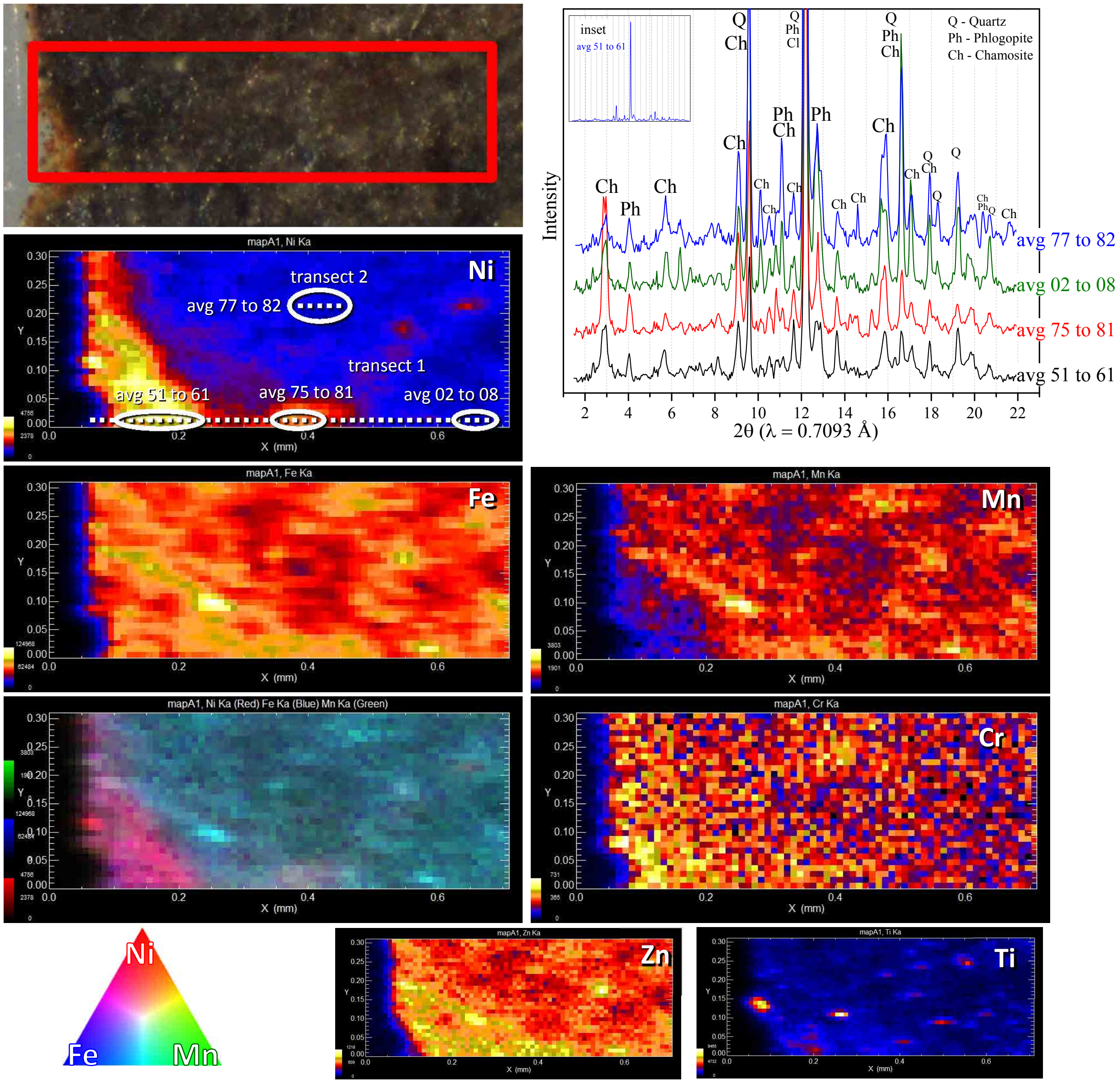

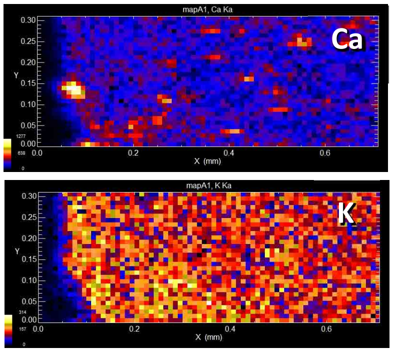

The color scale indicates relative elemental concentration measured in counts per second (CPS). White is higher concentration (hotter). The highest white values for each elemental map are listed on each map. The lowest values (black, cooler) are always zero. Mn hotspots co-localized with Cr hotspots can be ignored as this is Cr Kb fluorescence.

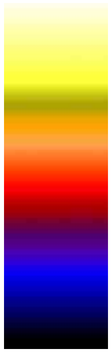

Figure S10: "s11unt" map A1

Recall that a guide to the µ-XRF maps of the "s11unt" thin section is provided in Figure S2. Map "A1" consists primarily of a large, green mineral. Around the edge of this mineral there is an orange crust (left side of photograph). The red box on the photograph indicates the approximate area with elemental mapping. Elemental distribution within this map varied, with Fe being high throughout the mineral. However, the lower left edge of the map indicates an area with higher concentrations of Ni. The tricolor map shows that at this location there is association of both Fe and Ni (purple color). Zinc also appears to be distributed throughout the green mineral to a lesser degree.

Although the tricolor map indicates Mn is associated with Fe (blue-green color), the Mn counts are significantly lower (ca 3800 versus 125000). Four µ-XRD transects were carried out, with their locations selected based on differences in Ni distribution. Scans for those transects were averaged into four separate spectra (identified as "77 to 82", "51 to 61", "75 to 81", and "02 to 08"). Despite the elemental heterogeneity of the locations of each averaged transect, the spectra have nearly identical diffraction patterns. Quartz, phlogopite, and chamosite are identified as the major minerals (Table 1). Chamosite and quartz share several peaks matched here. Chamosite is an Fe bearing phyllosilicate (35). Given the high Fe content of ultramafic rocks, the high CPS of Fe in this mineral in particular, and the common presence of chlorite minerals in these samples, the identification of chamosite is in agreement with the chemical composition of this mineral. The higher Ni CPS in "51 to 61" than in "77 to 82" or "02to08" illustrates that over a 500 µm scale, elemental heterogeneity of Ni in one specific mineral (for example, chamosite and phlogopite) can vary six to seven times (based on a ratio of CPS of Ni).

Figure S11 - s11unt map A2 with µ-XRD

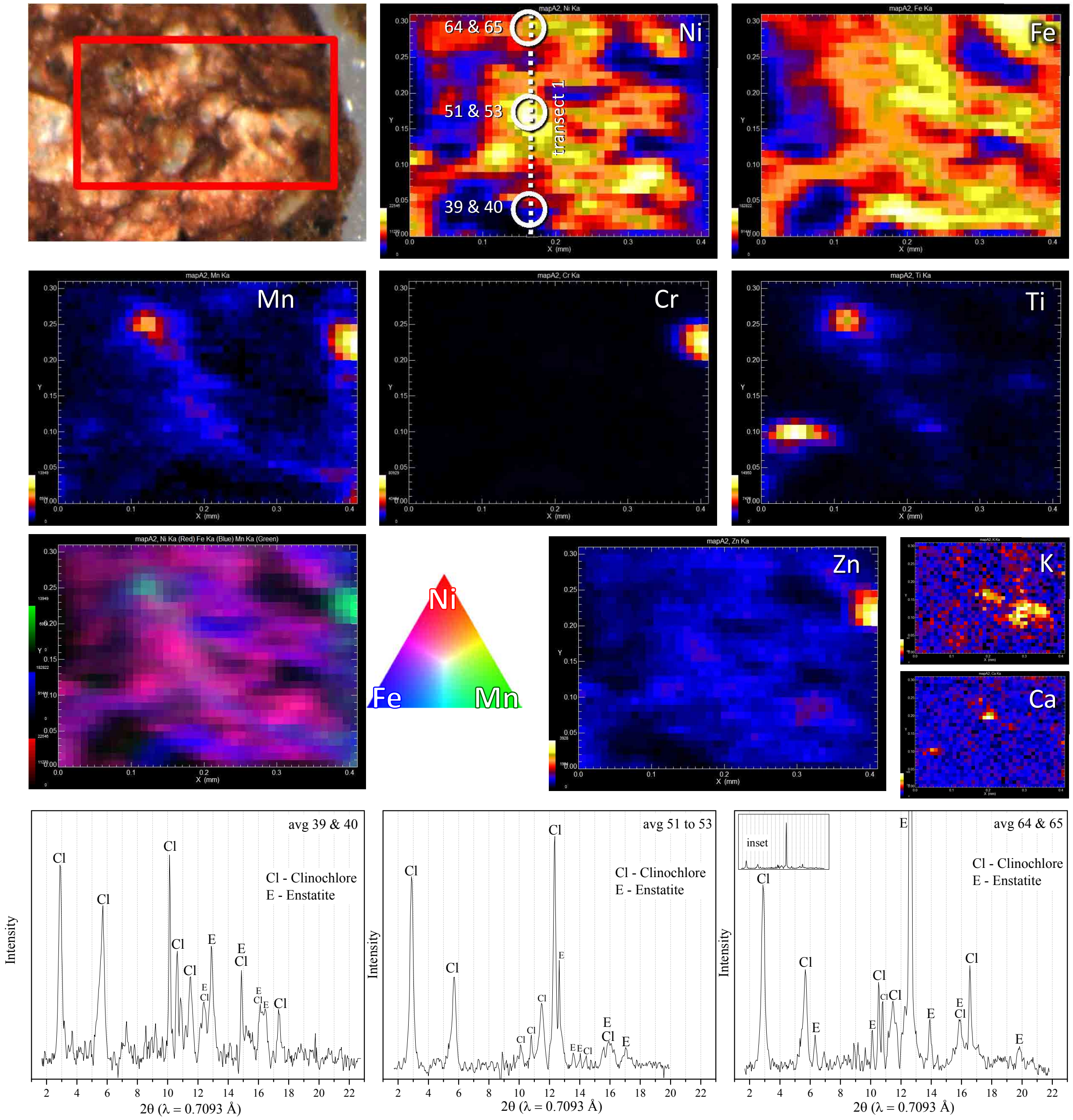

The color scale indicates relative elemental concentration measured in counts per second (CPS). White is higher concentration (hotter). The highest white values for each elemental map are listed on each map. The lowest values (black, cooler) are always zero. Mn hotspots co-localized with Cr hotspots can be ignored as this is Cr Kb fluorescence.

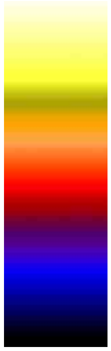

Figure S11: “s11unt” map A2

This map contains one μ-XRD transect, which passes through one Fe and two Ni hotspots. Scans from the transect were averaged together to form three spectra (“avg 39&40”,” avg 51 to 53”, and “avg 64&65”). Elemental correlations in this map indicate that Ni and Fe are highly correlated (purple color in tricolor map). Mn in the upper left corner is not correlated with Ni or Fe. On several occasions in the maps from NSLS, Cr Kβ fluorescence is included with the Mn Kα fluorescence, and thus Cr appears in the Mn maps. See Text S2.1 for discussion on this artifact. This occurs here with the Cr hotspot on the right side of the map. Thus in the tricolor plot green also appears where Cr is present. Cr is however associated with the Zn hotspot. The photograph provides visual cues to the heterogeneity of this map.

Upon first glance, the photograph indicates a more heterogeneous mineral phase than that found in, for example Figure S10; however, the transect diffractograms for Ni and Fe hotspots indicate that the mineralogy is similar throughout the entire transect, including both clinochlore and enstatite. Thus here it is evident that enstatite can host various amounts of Ni and Fe in its structure. Enstatite is chain inosilicate mineral found in the bulk-XRD patterns of “s11unt”, “s10t2”, and “s20unt” (1).

Figure S12a - s11unt map B1 with µ-XANES and µ-EXAFS

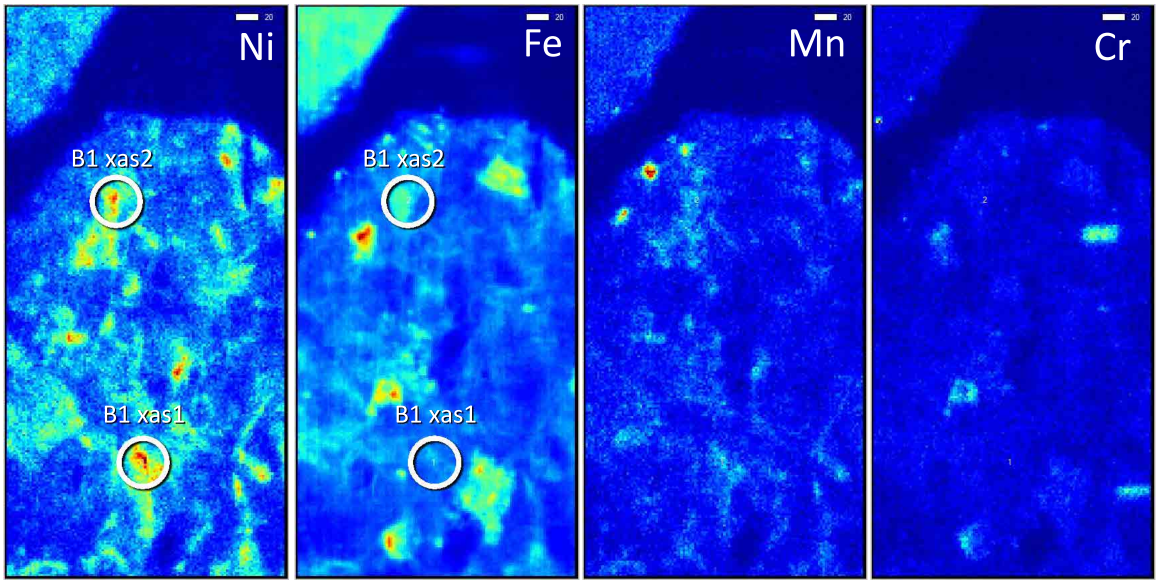

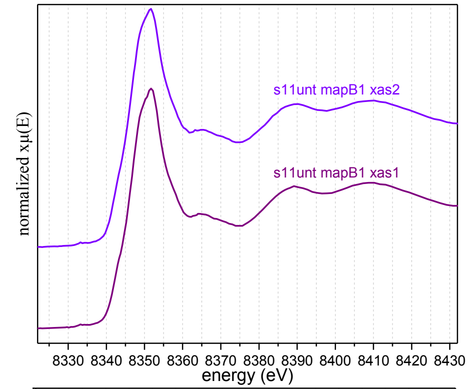

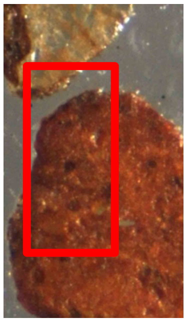

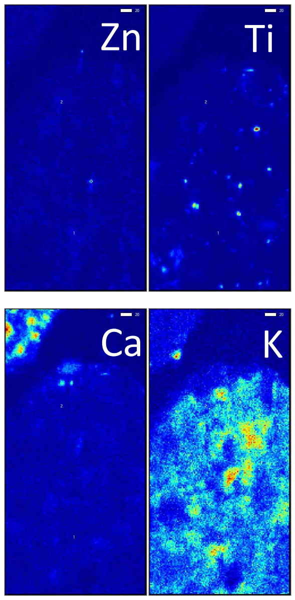

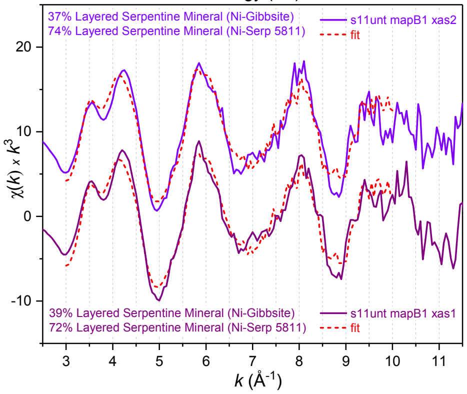

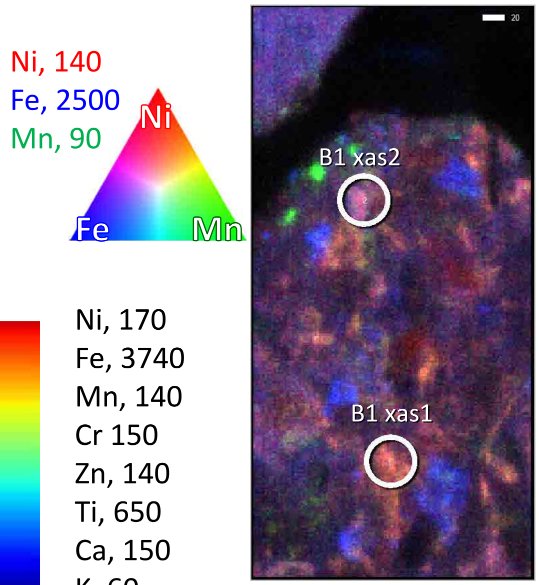

The color scale on the right indicates relative elemental concentration measured in counts per second (CPS). Red is higher concentration (hotter). The highest red values for each elemental map are listed to the right. The lowest blue values (cooler) are always zero. Scale bar is in µm.

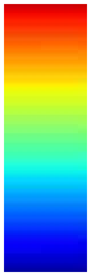

Figure S12b - s11unt map B1 (cont.) with μ-XRD

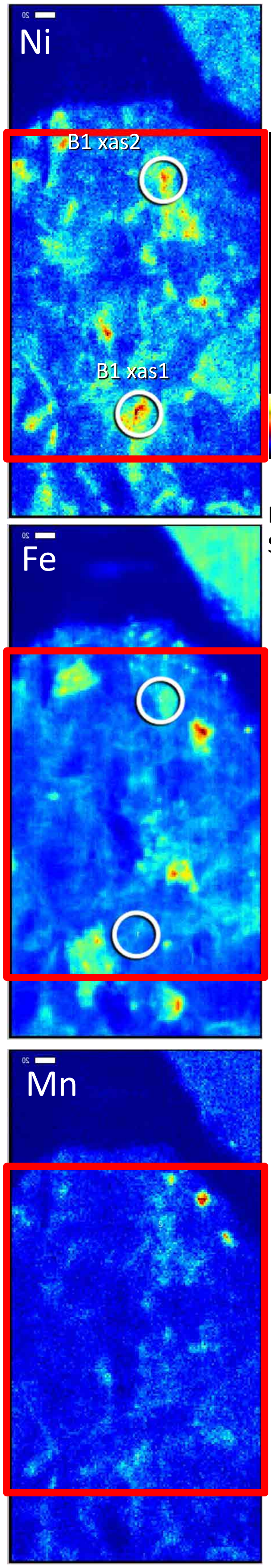

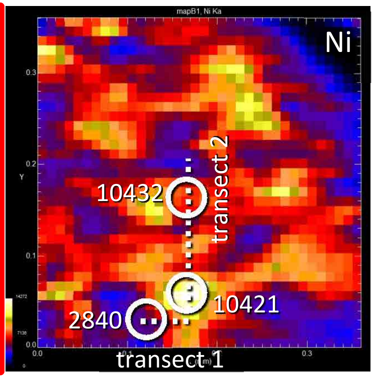

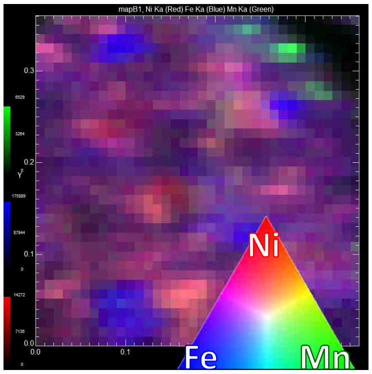

Note:
SSRL maps flipped horizontally

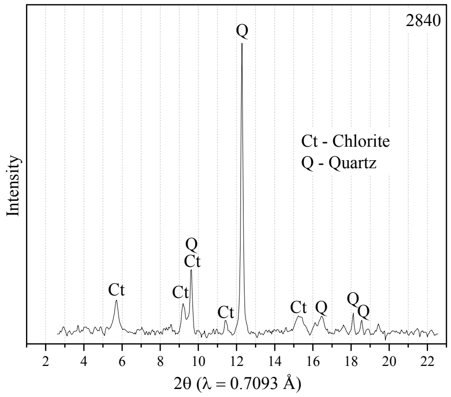

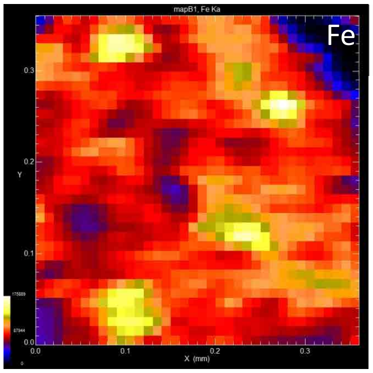

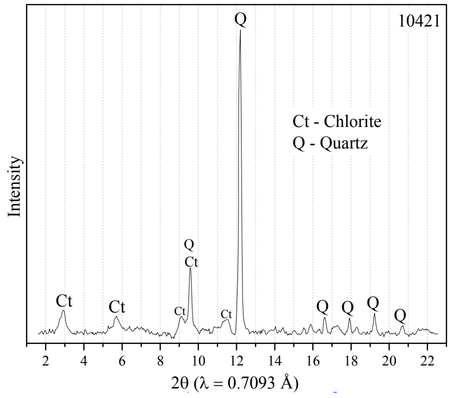

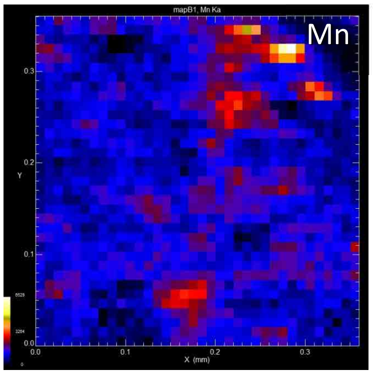

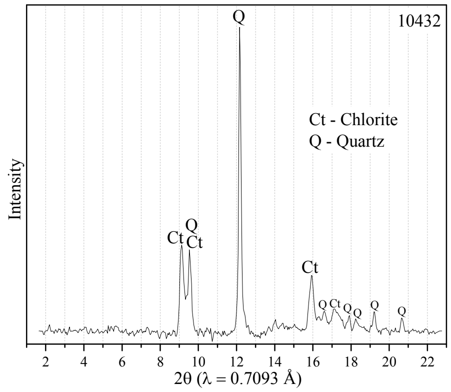

Figures S12a and S12b: “s11unt” map B1

This map consists mostly of a red colored mineral (see red box in the photograph). The red color may indicate that perhaps Fe would be the dominant element to fluoresce because oxidized Fe displays reddish color (for example, hematite). However, the fluorescence from Fe only has several isolated hot spots; moreover, the transparent/greenish mineral in the upper left corner displays more consistent and higher levels of Fe than the red mineral. The tricolor map illustrates sample heterogeneity between Ni, Fe, and Mn. Two spots were selected for spectroscopy (“B1xas1” and “B1xas2”). For this sample monochromator glitches were not a problem, so the full XANES and EXAFS regions could be acquired. In the XANES region, there are large indentations at 8400 eV in both spectra. This generally indicates that Ni is in a layered octahedral sheet and surrounded by lighter atoms such as Mg (1). At “B1xas1”, low CPS of Ni are present with low amounts of both Fe and Mn. At “B1xas2”, Ni is present with Fe and to a lower extent Mn.

The LCF results presented in Figure S12a and Table 3 indicate the best fits obtained using the standards. Both “B1xas1” and “B1xas2” have low counts of Ni, which is a maximum of 170 CPS in this map. In these two spots, low concentrations of Ni are associated with low concentrations of both Fe and Mn, although spot “B1xas2” has a slightly higher amount of Fe. The μ-EXAFS spectra for both spots are similar structurally with large indentations at 3.7 $Å^{-1}$. Many of the best fits for “B1xas1” required the Ni-gibbsite and serpentine mineral standards. This was expected because of the large indentation at 3.7 $Å^{-1}$. For “B1xas1”, 39% of the Ni-gibbsite standard and 72% of the Serpentine 5811 standard contributed to the fit. Discussion as to why the Ni-Gibbsite standard is a good analogue for this type of atomic environment is given in in Text S2.3 and Figure S16a/b. The F-test indicated an 80% probability that adding a third standard would only improve the fitting of noise; thus two standards were appropriate.

For “B1xas2”, which was obtained from the same red mineral, the LCF results were similar to “B1xas1”. Many of the best fits required the use of the Ni-gibbsite and serpentine mineral standards; two standards composed the best fit (37% Ni-Gibbsite and 74% Ni-Serp 5811). Only two standards were used with the fit, with a 64% probability that adding a third standard would improve only the fitting of noise. Thus, most of the Ni in the low concentrations here is bound in the octahedral layer of serpentine related minerals, such as lizardite, which is the principal mineral in all the serpentine mineral standards [see (1) for bulk XRD patterns of serpentine mineral standards]. This is in agreement with the μ-XRD data presented in Figure S12b of spot “B1xas1” because the octahedral layers of lizardite and chlorite are similar in structure.

Figure S12b is a continuation of data for sample “s11unt” map B1 and contains μ-XRD transects. This sample was re-mapped at the NSLS to perform μ-XRD (black outlined maps). Note that the blue-colored SSRL maps are flipped horizontally from the previous figure to align them with the NSLS maps. The red boxes on the blue SSRL maps indicate the regions where the NSLS maps were taken. The alignment of Ni, Fe, and Mn fluorescence are accurate between maps at NSLS and SSRL. Again, this illustrates the utility of analyzing thin section samples on different beamlines where various techniques are available. Two diffraction transects were carried out on the NSLS map resulting in three diffractograms presented (labeled “2840”, “10421”, and “10432” on map).

“B1xas1” and spot “10421” are where μ-XAS and μ-XRD data were collected, respectively, and are the same location. This location is the second of five spots in this study

where both μ-XRD and μ-XAS techniques were carried out. The spectroscopic evidence agrees well with the diffraction data because chlorite was one of the two minerals identified. This mineral is a layered phyllosilicate, which provides the correct structure to allow of incorporation of light Mg atoms around central absorbing Ni atoms to cause the indentation at 3.7 $Å^{-1}$ (8400 eV). Peaks for quartz were also identified in all three diffraction spectra.

The elemental variability in each spot, either high in Ni or Fe or the presence of both, illustrates the breadth of composition that can be present in the chlorite mineral. Additionally, because of the consistency in the diffraction patterns among the three spots and given that other samples have shown similar diffraction patterns over a variety of chemical compositions (for example, “s11unt” map A2 and “s11unt” map A1), it is likely that “B1xas2” (Figure S12a) is also composed of chlorite and quartz. For this reason both “B1xas1” and “B1xas2” have the indentation at 3.7 $Å^{-1}$.

Figure S13 - s11unt map B2 with μ-XRD

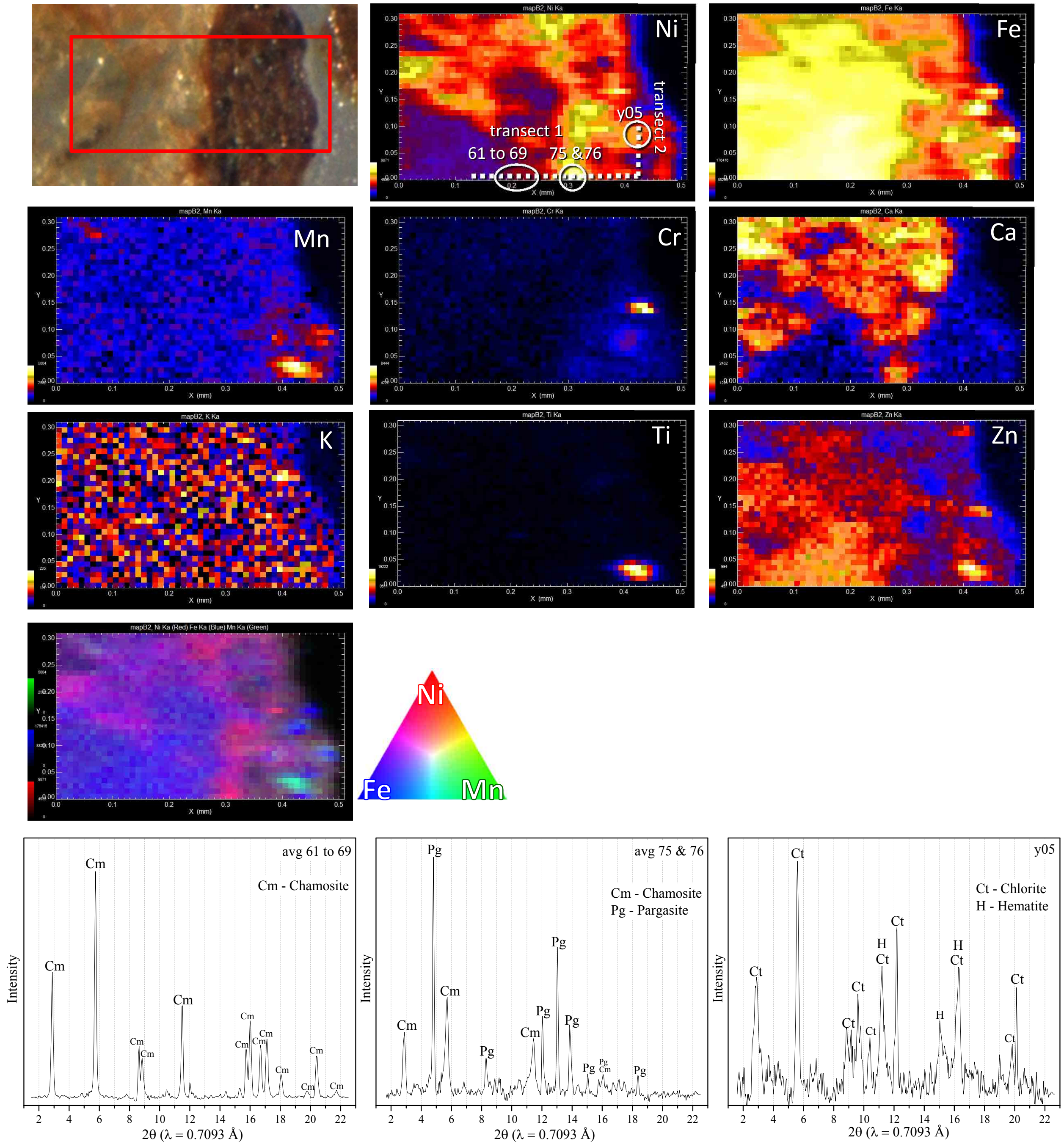

The color scale indicates relative elemental concentration measured in counts per second (CPS). White is higher concentration (hotter). The highest white values for each elemental map are listed on each map. The lowest values (black, cooler) are always zero. Mn hotspots co-localized with Cr hotspots can be ignored as this is Cr Kb fluorescence.

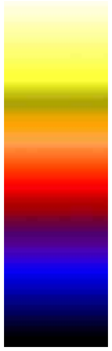

Figure S13: "s11unt" map B2

This map, outlined in the red box on the photograph, covers areas where a continuous mineral (left side) transitions to agglomerated minerals (right side). Elemental maps indicate that the greenish mineral on the left is predominantly Fe-rich, with several dispersed areas of Ni. Zinc also appears to associate in the large area of Fe. There is one patch of Mn in the lower right corner which correlates with Ti. Two µ-XRD transects were carried out on this map, and several spectra were averaged together to yield three diffraction patterns. The first spot, "avg 69 to 61", is predominantly Fe and the diffraction peaks identify this mineral as chamosite, which is an Fe bearing chlorite mineral.

Moving to the right, the spot "avg 75&76" also shows distinct peaks for chamosite but additional peaks that match with pargasite also appear. Pargasite, like enstatite, is a chain inosilicate mineral and can occur in altered ultramafic rocks (35). At spot "avg 75&76" , which is just off of the hot Fe area of chamosite, Ni CPS increase dramatically, and the presence of chamosite and pargasite indicate these minerals can host Ni. Small pargasite peaks were identified in the bulk XRD of "s11unt" (1), so their presence here is reasonable. Lastly, in spot "y05" in the agglomerated minerals area, diffraction peaks for both chlorite and hematite are produced from this hotspot of Ni and Fe.

Figure S14 - s11unt map C with μ-XRD

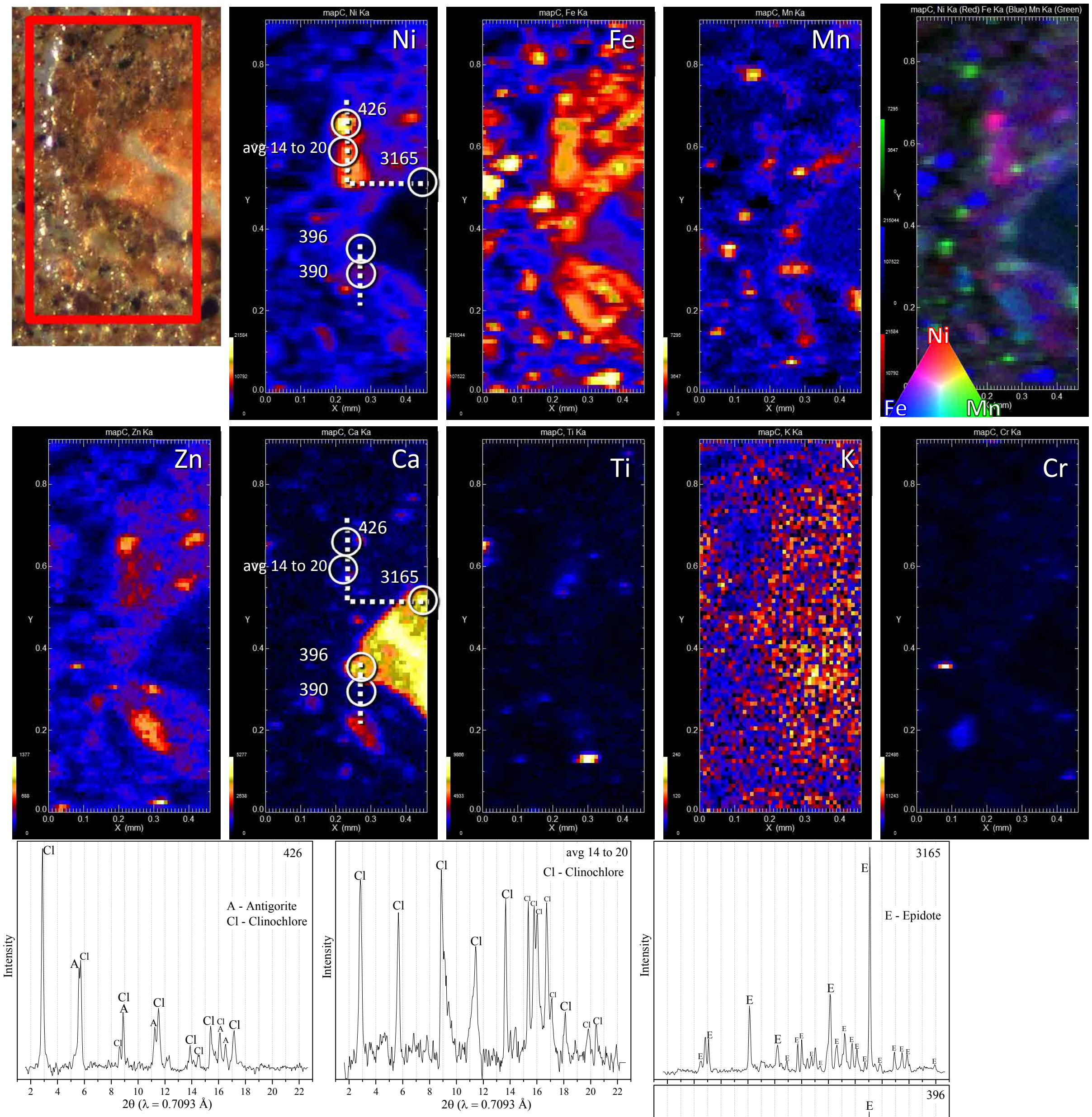

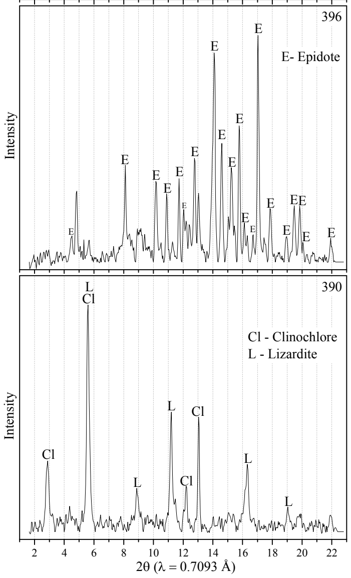

The color scale indicates relative elemental concentration measured in counts per second (CPS). White is higher concentration (hotter). The highest white values for each elemental map are listed on each map. The lowest values (black, cooler) are always zero. Mn hotspots co-localized with Cr hotspots can be ignored as this is Cr Kb fluorescence.

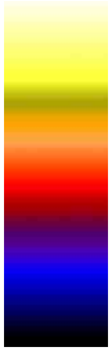

Figure S14: “s11unt” map C

Serpentine soils are known for their low Ca concentrations; however, in this figure there is a triangular mineral on the right side of the map which is high in Ca and unassociated with other elements. In the photograph, the map area is outlined by a red box, and the entire map covers an area of agglomerated minerals above and below the Ca-rich mineral. In several regions, Ni and Fe correlate together as seen in the tricolor plot (purple color). Mn tended to not associate with Ni or Fe in this sample; however, just below the Ca-rich mineral there is a region where Ni, Fe, and Mn associate. Zinc associated with Ni in several locations in this sample, but Zn did not commonly associate with Ni in other samples.

Several hotspots of Ni, Fe, Mn, and Ca were chosen for μ-XRD, and in total five diffractograms are presented (“426”, “avg 14 to 20”, “3165”, “396”, and “390”). At spot “426”, where Ni, Fe, and Zn associate, peaks for clinochlore and antigorite were identified. In the Ni/Fe rich mineral just below (“avg 14 to 20”), only clinochlore peaks were matching. Two diffractograms were acquired on the Ca-rich mineral (“3165 and “396”), and both indicate that this mineral is epidote, which is a Ca-rich mineral. Just below the epidote tip, there is a Ni, Fe, and Mn hotspot (“390”). Peaks for clinochlore and lizardite were identified, again illustrating the elemental heterogeneity that can be contained in those phyllosilicates.

Figure S15 - s11unt map D1 with μ-XRD

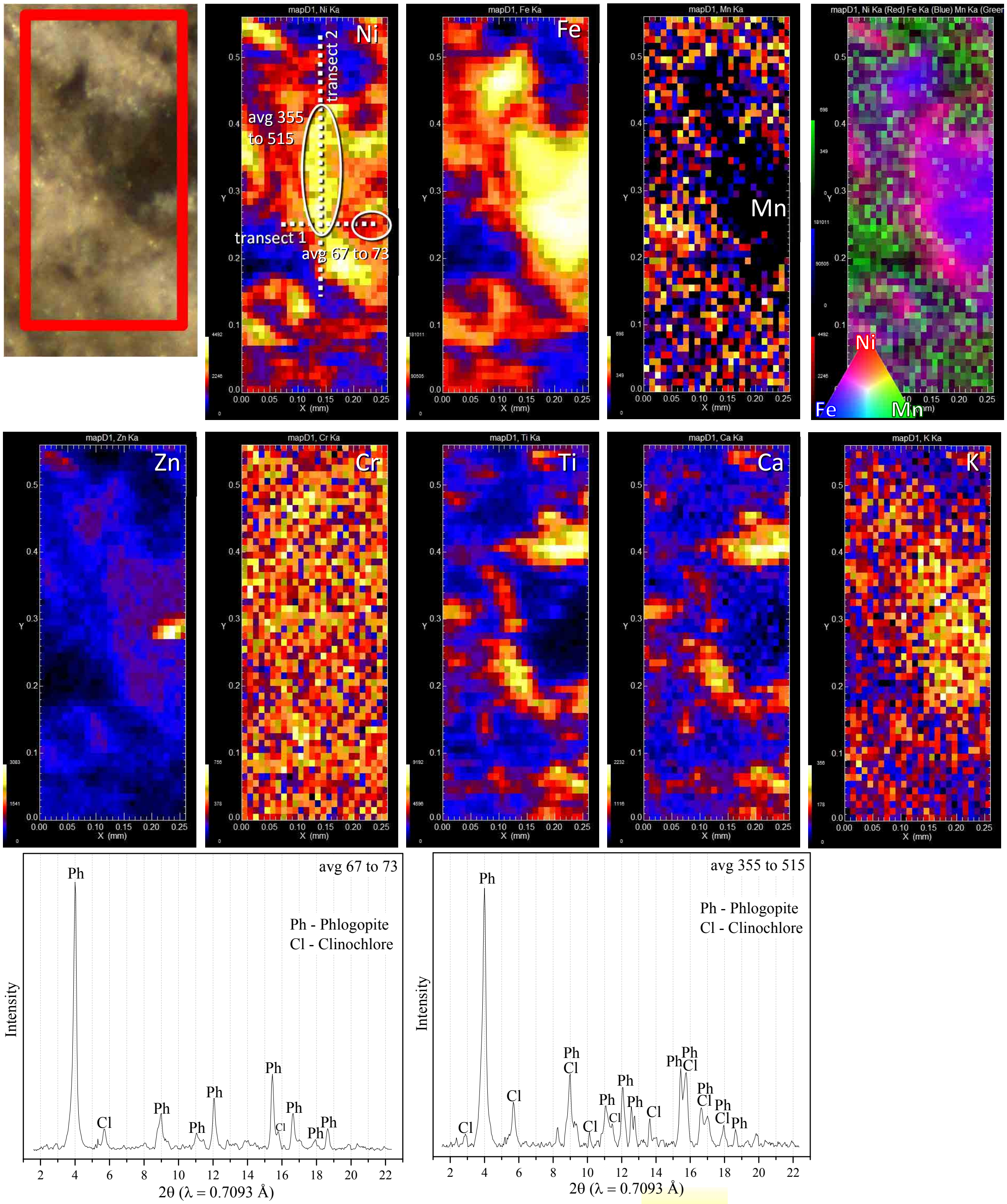

The color scale indicates relative elemental concentration measured in counts per second (CPS). White is higher concentration (hotter). The highest white values for each elemental map are listed on each map. The lowest values (black, cooler) are always zero. Mn hotspots co-localized with Cr hotspots can be ignored as this is Cr Kb fluorescence.

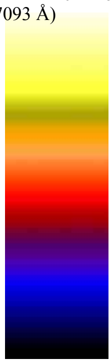

Figure S15: “s11unt” map D1

This map was acquired over the interior portion of a large particle with dark green to light green color (see Figure S2 for larger photographs). Because of the large size of this particle, both diffractograms presented are averages of multiple spectra along each of the two transects. In transect 1, the spectra “avg 67 to 73” includes a region that is high in Fe. Spot “avg 355 to 515” along transect 2 includes a Ni-rich region just on the boarder of the Fe hotspot, although this area also includes lower Fe CPS. The tricolor map indicates where Ni and Fe are correlated. In the tricolor map, Mn appears dispersed throughout because of its low CPS (698) versus that of Ni and Fe (4492 and 181011, respectively). The hot area of Fe on the right side also includes a hotspot of Zn. Titanium is distributed throughout the perimeter of the Fe hotspot. The diffractogram of “avg 67 to 73” over the Fe hotspot yielded peaks for phlogopite and clinochlore. Peaks for phlogopite and clinochlore were found again in around the perimeter of the Fe hotspot where higher Ni CPS occurred in “avg 355 to 515”. The varied composition of phlogopite and clinochlore illustrates the diverse composition of elements they can host. Phlogopite was also identified in “s10t2” medium sand particles, “s11unt” map Al, and “s11unt” silt particles.

Figure S16a - s11unt map D2 with μ-XANES and μ-EXAFS

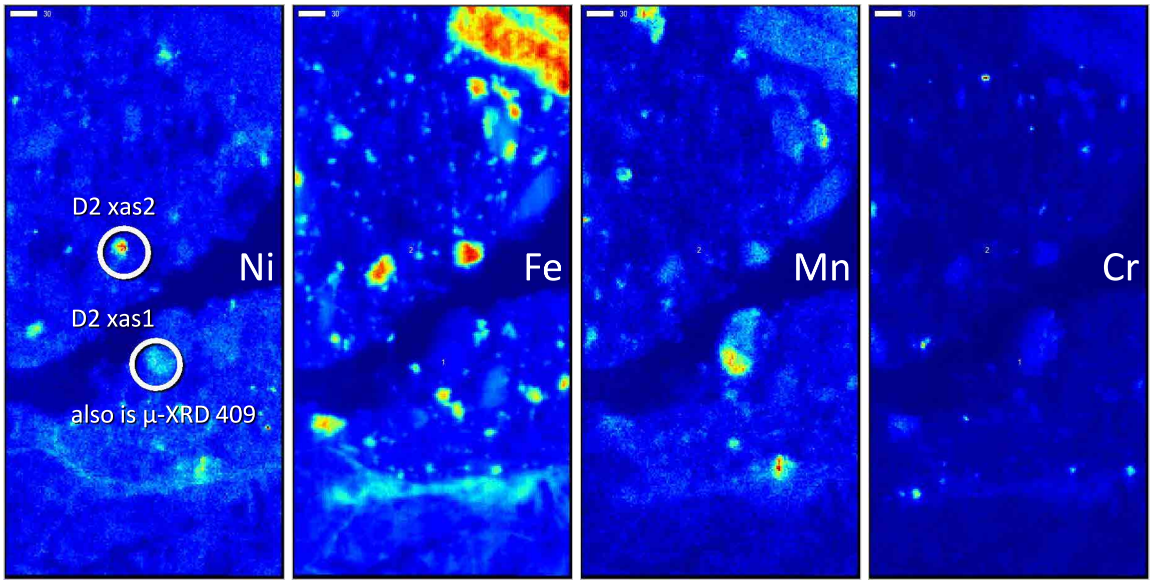

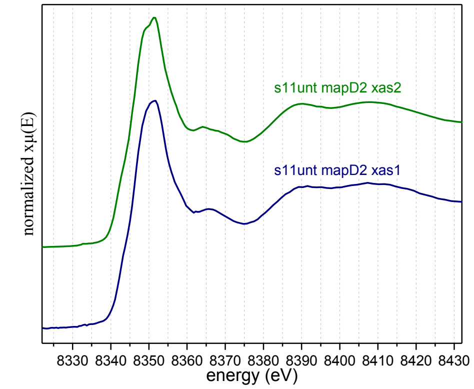

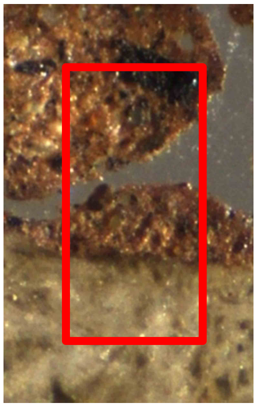

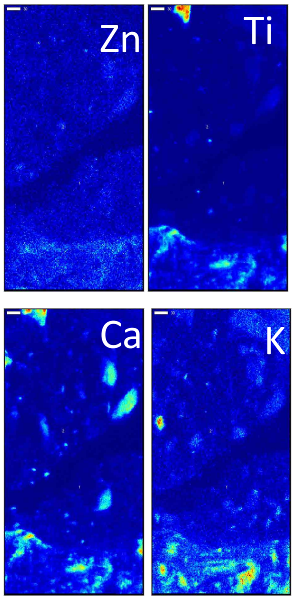

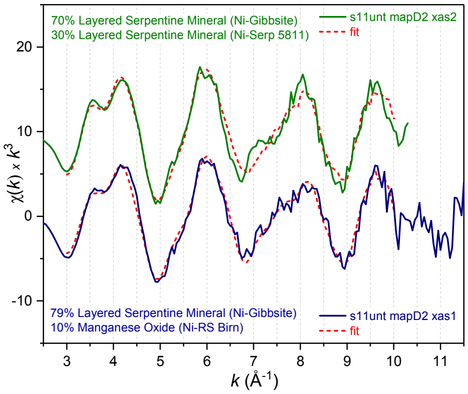

Ni, 80
Fe, 3000
Mn, 70

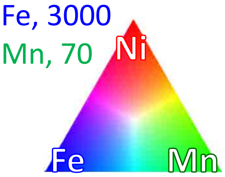

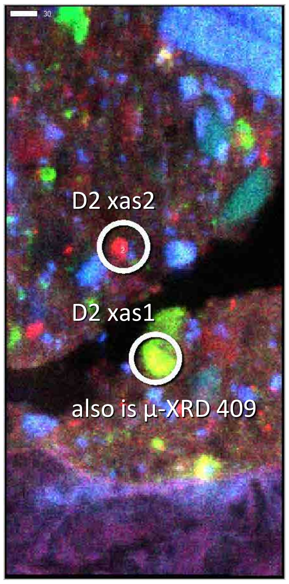

The color scale on the right indicates relative elemental concentration measured in counts per second (CPS). Red is higher concentration (hotter). The highest red values for each elemental map are listed to the right. The lowest blue values (cooler) are always zero. Scale bar is in μm.

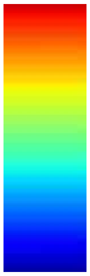

Ni, 220
Fe, 7550
Mn, 200
Cr, 390
Zn, 40
Ti, 740
Ca, 310
K, 70

Figure S16b - s11unt map D2 (cont.) with μ-XRD

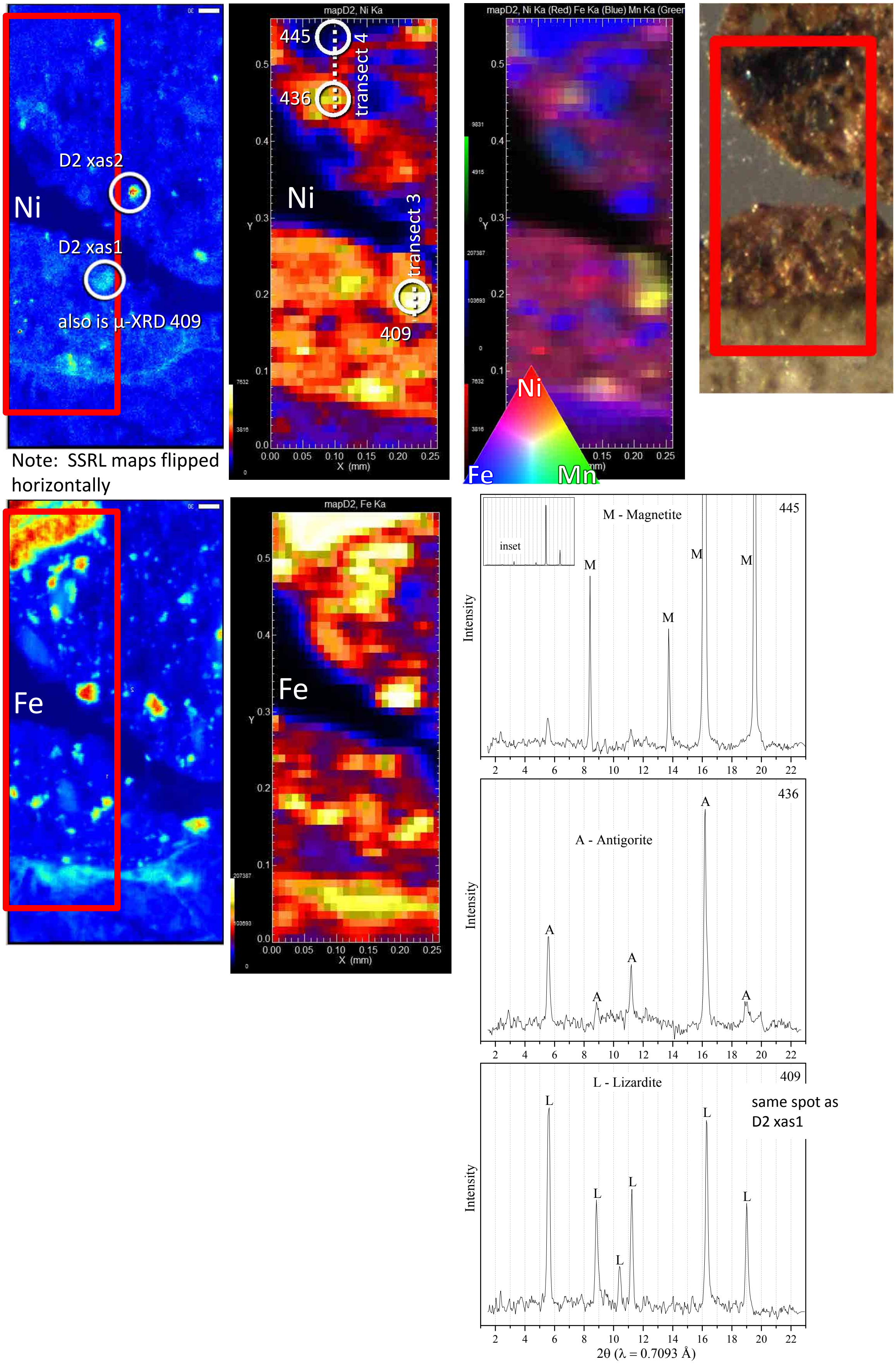

Figures S16a and S16b: “s11unt” map D2

The map area includes portions of agglomerated minerals above and below a gap in the center of the map. The elemental heterogeneity of these agglomerations is evident in the tricolor map, where multiple individual hotspots of Ni, Fe, and Mn, as well as several spots of associated metals (purple, yellow, light blue) are present. The lower part of the map covers a portion of the same mineral presented in Figure S15 (see Figure S2 for a larger photograph). In the upper right of the map there is a hotspot of Fe associated to the black mineral in the photograph. Because of the elemental heterogeneity in this sample, two spots were selected for µ-XAS data acquisition, “D2xas1” and “D2xas2”. Spot “D2xas1” has higher CPS both Ni and Mn (yellow-green color) while only elevated Ni CPS are present in “D2xas2” (red color).

Despite their elemental differences both µ-XANES spectra exhibit an indentation in the oscillation at 8400 eV. This indentation indicates that Ni is likely present in the octahedral sheet of a layered silicate mineral and surrounded by lighter elements such as Mg (1), which also fit into the octahedral position (33). Even though “D2xas2” is rich in both Mn and Ni, the indentation is pronounced, albeit slightly less so than “D2xas1”. The decrease in this indentation is probably due to the increase in population (amount) of other trace metals such as Mn in the octahedral sheet (15, 36).

The EXAFS spectra also highlight the indentations, which convert to a split in the first oscillation at 3.7 $Å^{-1}$. Again in spot “D2xas1” this split is slightly less pronounced than in spot “D2xas2”, probably due to the higher Mn concentration. The Mn would also occupy octahedral sites around the Ni and thus decrease the splitting effect caused by the neighboring Mg (see Section 3.2.3 in the main text for further discussion). “D2xas1” is the third of five spots in this work where both µ-XRD and µ-XAS data were obtained from the same spot. The µ-XRD spectrum “D2xas1” is shown in Figure S16b as spectrum “409”, and this corroborates that the mineralogy is correct for an octahedral environment of Ni with neighboring Mn atoms (lizardite).

LCF was also carried out on µ-EXAFS obtained from this spot (Figure S16a, Table 3). When considering the LCF of this sample, the best fits included Ni incorporated into an octahedral layer of a serpentine-related mineral, which here the analogue is the Ni-Gibbsite standard. Because the Ni-Gibbsite standard illustrates the pronounced splitting feature at 3.7 $Å^{-1}$ (Figure S24) and gibbsite is octahedrally layered similarly to serpentine minerals, it is included in the “Layered Serpentine Mineral” category (see Text S2.3 for further discussion on the categorization of standards). The µ-XRF maps illustrate the dominance of Mn and Ni at this spot. Thus, the manganese oxide standards were considered to be potentially important components. If any Mn oxides are present, the best fit would include Ni-Gibbsite (79%) and Ni bound to birnessite (Ni-RS Birn, 10%). However, it is also possible that the Mn is completely bound within the lizardite structure. This is reasonable because the major µ-XRD peaks indicated the presence of only lizardite (Figure S16b). The F-test indicated a 28% probability that the use of three standards improved only the fitting of noise. Because 28% is well above the 5% cutoff limit for fit improvement, it was concluded that only two standards were appropriate for this fit.

For “D2xas2”, the hotspot consisted mainly of Ni (Figure S16a tricolor map, Table 3). The fits with the lowest R-factors for this spectrum included three standards, one of which was a ferrihydrite standard. However, because Fe CPS are low at this spot and the improvement in the fit was not statistically significant versus those with two standards, the fits with ferrihydrite were discarded. Additionally, the fits with ferrihydrite failed to capture the shoulder split at 3.7 $Å^{-1}$, which is a critical feature relevant to the elemental contents and multiple scattering within an

octahedral sheet. Thus, the best fit which accurately captures the split feature included two standards (70% Ni-Gibbsite and 30% Ni-Serp 5811). A deficiency in this fit, however, is at 7.2 $Å^{-1}$, where a shoulder is present in the sample data, and the fit does not closely follow that shoulder. The F-test produced a 41% probability that using three standards would give statistical improvement in the fitting noise, thus only two standards were considered for this fit. Microfocused-EXAFS spectra collected from physically close (250 µm) yet elementally distinct agglomerated particles (“D2xas1” and “D2xas2”) had similar LCF results, with a majority of Ni being hosted in layered octahedral sheets.

Figure S16b is a continuation of data from sample “s11unt” map D2. Here the same sample area was re-mapped at the NSLS to obtain µ-XRD data. One of the two µ-XAS spots was captured in the new map (D2xas1) and is labeled “409” on transect #3. This spot is hot in both Ni and Mn, and the diffraction data show peaks that match with lizardite. Lizardite can thus include both Ni and Mn simultaneously in its octahedral layer. The indentation of the first EXAFS oscillation at 3.7 $Å^{-1}$ indicated Ni is located in a layered octahedral sheet, and lizardite fits this structure. Lizardite is a layered phyllosilicate serpentine mineral. The hotspot of Fe “445” at the top left of the map (SSRL maps are flipped horizontally) coincides with the black mineral in the photograph, and peaks for magnetite (which is commonly black in color) are identified. Down transect #4 at “436”, where Ni, Mn, and lower amounts of Fe are present, peaks for antigorite are identified, which again illustrates the breath of elements that can be hosted in serpentine minerals. Several of these peaks are at similar 2θ positions to lizardite because the two are related serpentine minerals.

Figure S17 - s11unt map E with µ-XRD

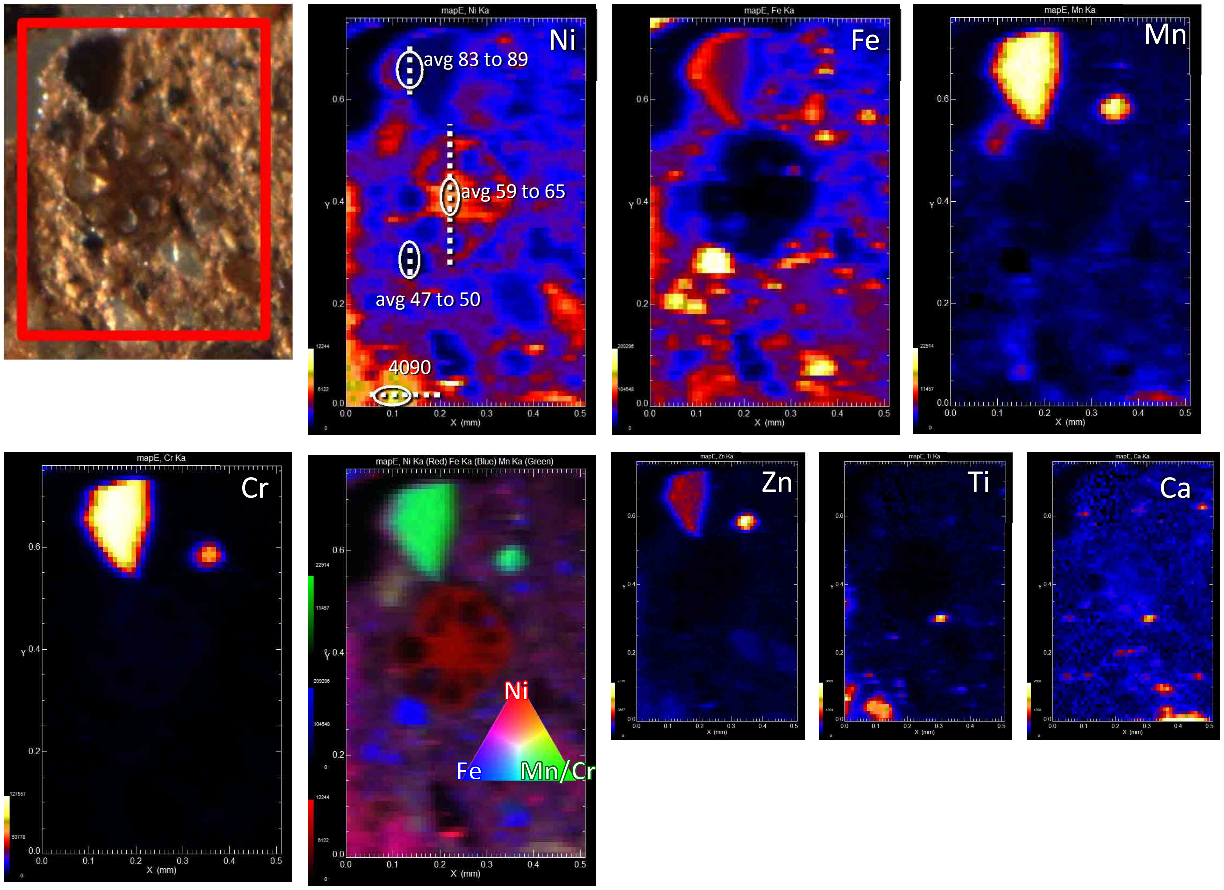

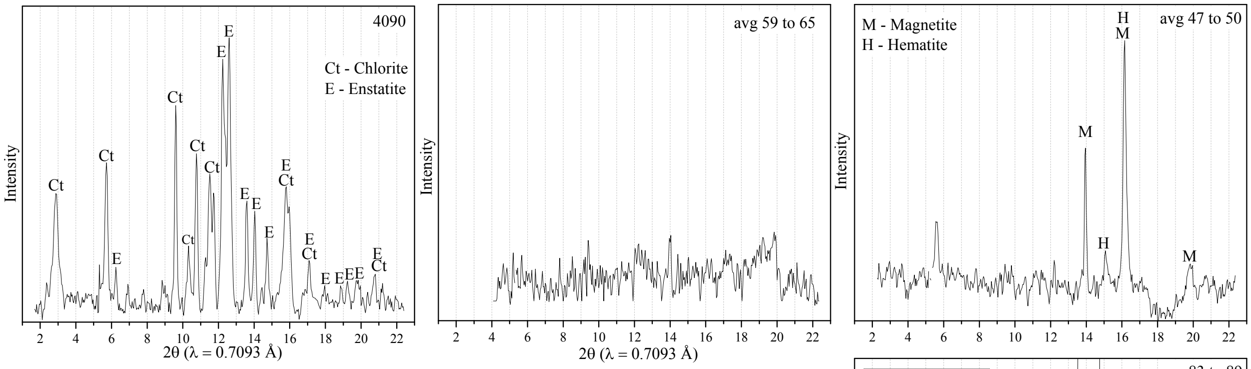

The color scale indicates relative elemental concentration measured in counts per second (CPS). White is higher concentration (hotter). The highest white values for each elemental map are listed on each map. The lowest values (black, cooler) are always zero. Mn hotspots co-localized with Cr hotspots can be ignored as this is Cr Kb fluorescence.

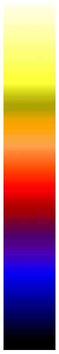

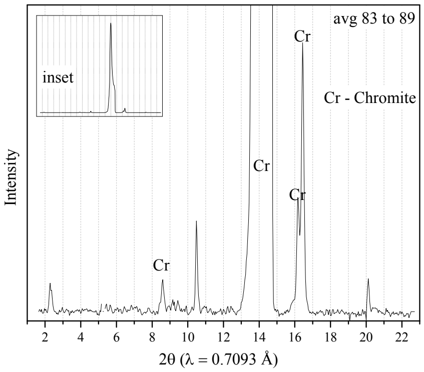

Figure S17: “s11unt” map E

Near the center of the map (red box on photograph) is a circular mineral with semi-symmetric holes in it. While these holes are void of fluorescence, the circular mineral itself has elevated amounts of Ni unassociated with other elements (red color in tricolor map). This gives it unusual morphology. There are several regions where Fe is unassociated with other elements (blue color on tricolor map). Large and small black chromite minerals are in the upper portion of the map, and Cr Kβ fluorescence is entering into the fluorescence window of the Mn Kα (see Text S2.1 for discussion on this topic). This artifact can be ignored, and there are only two regions where Ni and Mn associate (yellowish color on tricolor map). One is to the left just below of the large chromite particle, and the other is at the bottom right of the map.

Four µ-XRD figures are presented. Peaks from “4090” at the bottom of the map are attributed to both chlorite and enstatite, indicating that Ni and Fe are associated together in those minerals. Enstatite is chain inosilicate mineral found in the bulk-XRD patterns of “s11unt” (1). For the µ-XRD pattern “59 to 65” from the unassociated Ni-rich mineral with unusual morphology, no diffraction peaks were detected because of either 1) beam attenuation by the mineral, or 2) the diffracted beam did not hit the detector. Table 3 summarizes the minerals identified via µ-XRD and also lists samples where no diffraction peaks were measured by the detector. On the Fe hotspot, “avg 47 to 50”, peaks for magnetite and hematite were found. Lastly, chromite peaks were matched for the Cr-rich mineral “avg 83 to 89”. The inset on the graph of “avg 83 to 89” shows a peak that is disproportionately large to the rest (see Text S2.2 explanation of this process).

Figure S18 - s11unt map F with μ-XRD

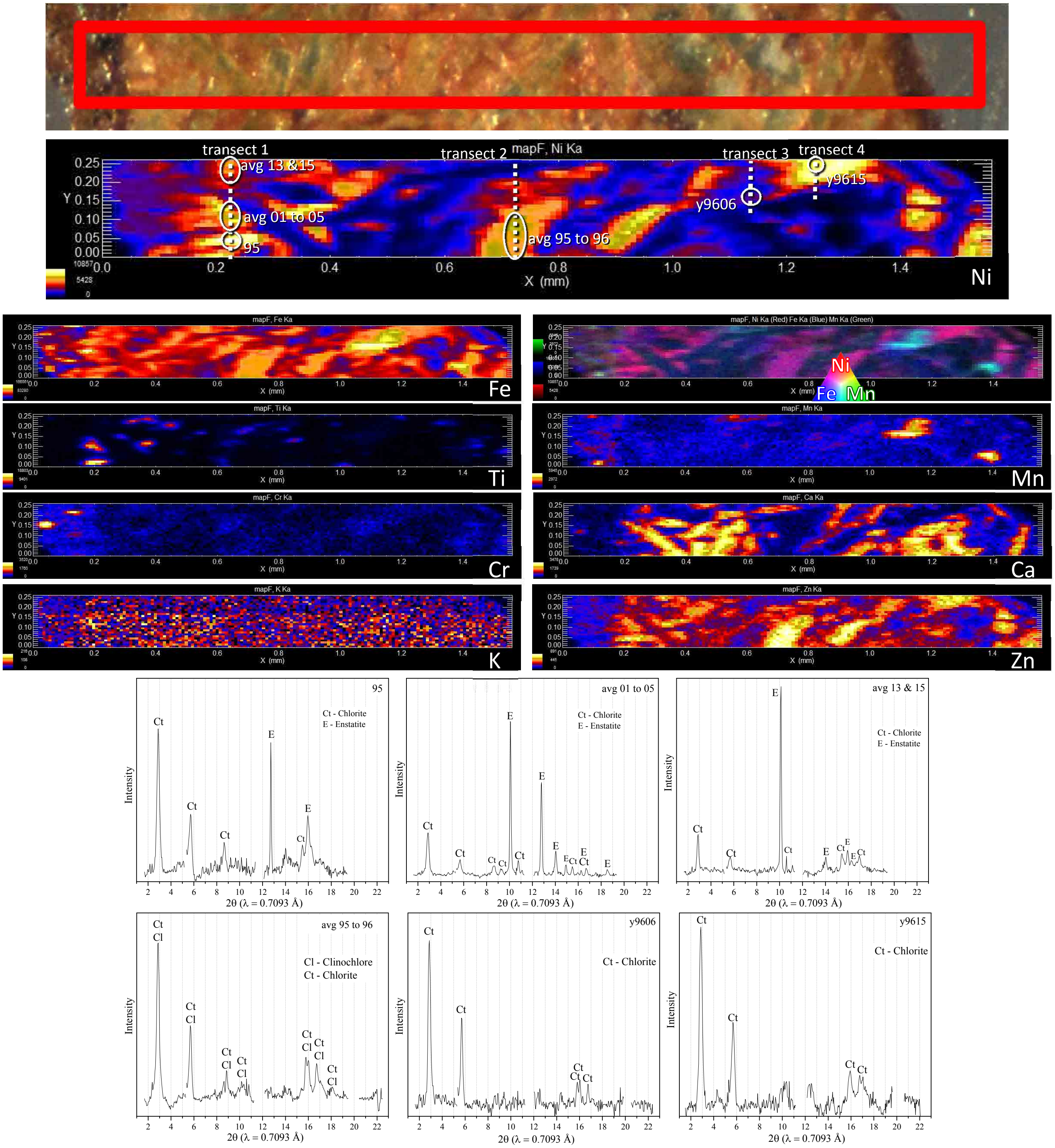

The color scale indicates relative elemental concentration measured in counts per second (CPS). White is higher concentration (hotter). The highest white values for each elemental map are listed on each map. The lowest values (black, cooler) are always zero. Mn hotspots co-localized with Cr hotspots can be ignored as this is Cr Kb fluorescence.

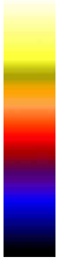

Figure S18: “s11unt” map F

Figure S18 is the last μ-XRF map of the “s11unt” petrographic thin section. It is a wide map (1.6 mm across) and a heterogeneously colored mineral (greenish to dark orange in photograph). Ni is often associated with Fe in this map (purple color in the tricolor map). Two pronounced regions (light blue on the tricolor map) indicate association of Fe and Mn. Calcium also appears to be inversely correlated to Ni, so they do not occur in the same locations.

Data from four μ-XRD transects are presented. In transect one, three diffractograms were analyzed, spots “95”, “avg 01 to 05”, and “avg 13&15”. In these spots, Ni is associated with Fe, and diffraction peaks consistently indicate chlorite and enstatite are present, again illustrating these minerals are common hosts for Ni and Fe. In the center of the map, spot “avg 95 to 96” is also rich in Ni and Fe and indicates peaks for clinochlore and chlorite. These two minerals have similar peaks and peak positions and are related minerals common in serpentine rocks. Moving to the right to transect three, “y9606” yielded peaks of chlorite, thus chlorite can also host Fe and Mn simultaneously. The last diffractogram presented is on transect four and is “y9615”. Again, peaks for chlorite were identified. Ni and Fe commonly occur with chlorite in this map over several discrete transects and hotspots.

Figure S19a - s11unt silt particles map

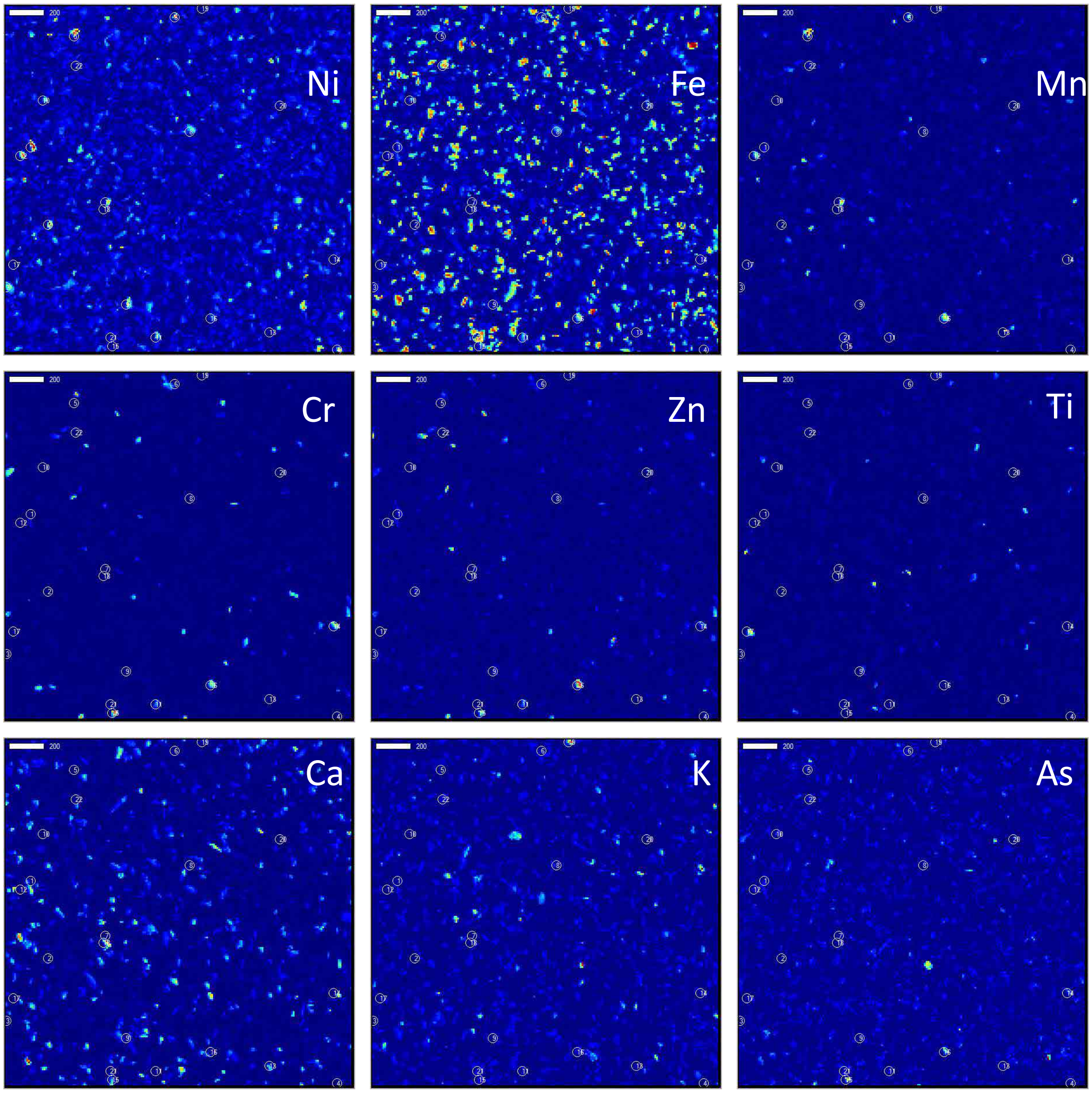

The color scale on the right indicates relative elemental concentration measured in counts per second (CPS). Red is the higher concentration. The highest red values for each map are below. The lowest blue values are always zero. Scale bar is in µm.

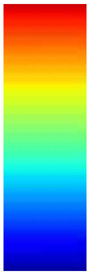

Ni, 1080
Fe, 45980
Mn, 4380
Cr, 17200
Zn, 600
Ti, 6960
Ca, 3000
K, 650
As, 140

Figure S19b - s11unt silt particles map (cont.) with µ-XRD

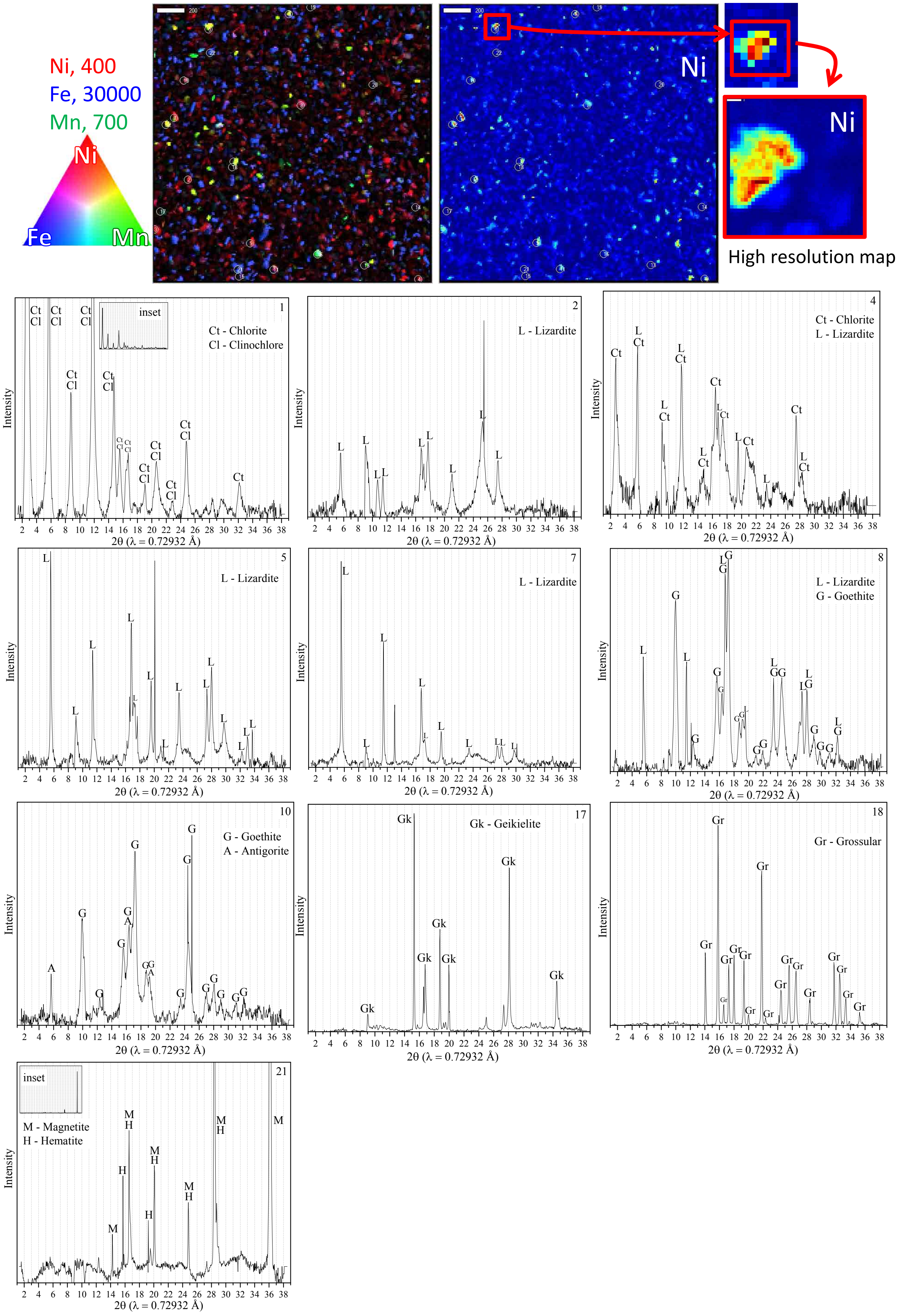

Figure S19c - s11unt silt particles high-resolution map with µ-XRD

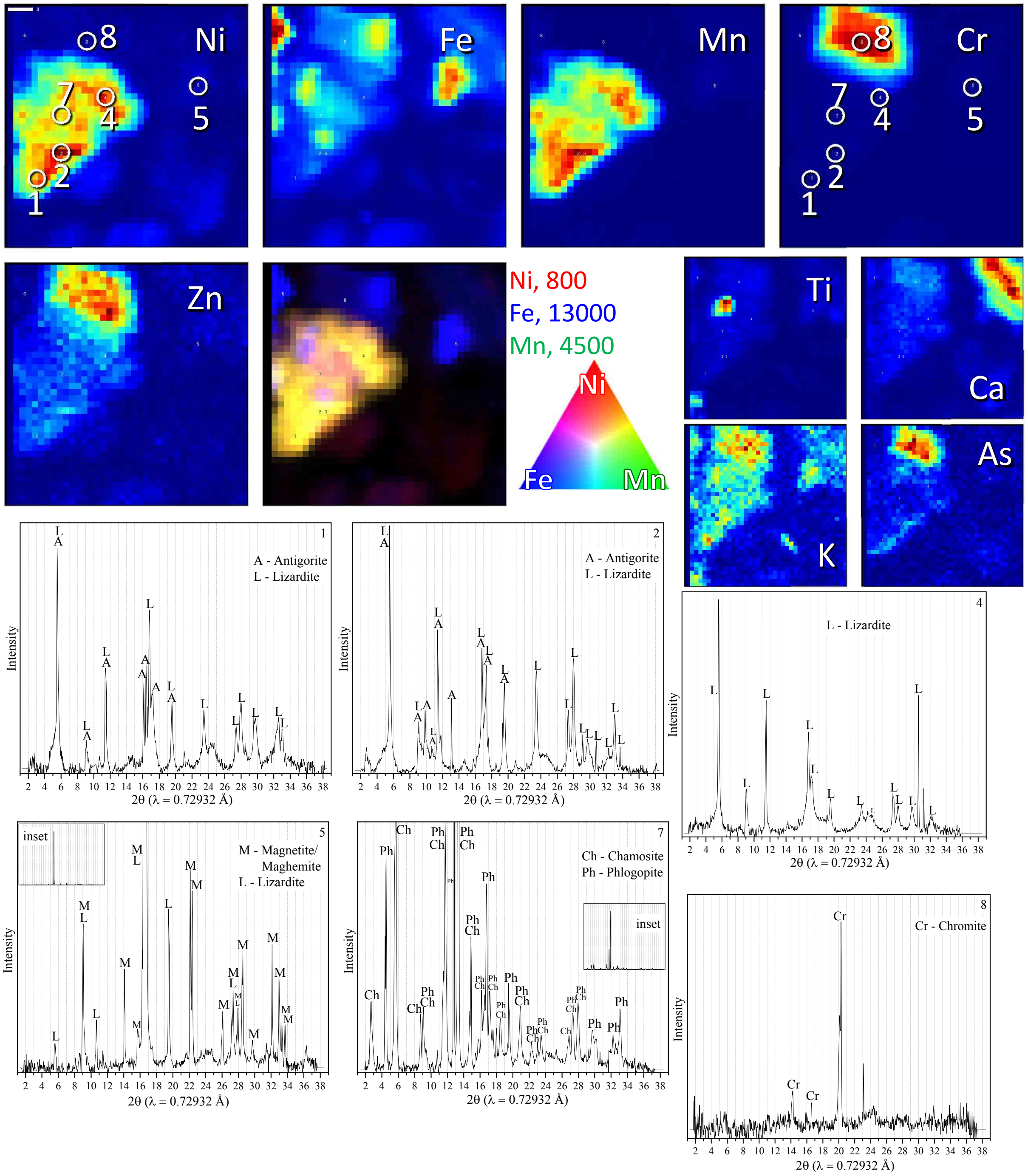

The color scale on the right indicates relative elemental concentration measured in counts per second (CPS). Red is the higher concentration. The highest red values for each map are below. The lowest blue values are always zero. Scale bar is in µm.

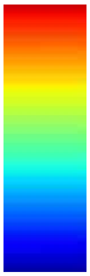

Ni, 1340
Fe, 21700
Mn, 5830
Cr, 13190
Zn, 200
Ti, 1580
Ca, 490
K, 80
As, 80

Figured S19a, S19b, and S19c: “s11unt” silt particles

Figure S19a is a map of the silt fraction of “s11unt” separated via sonication; it is not a petrographic thin section (Section 2.1). Multiple points on this map were selected for μ-XRD. These points are labeled with numbers and circles. The μ-XRD data for these maps are presented in Figure S19b. Of the labeled spots, only diffraction patters with clear peaks that could be identified are shown in Figure S19b. Elemental maps indicate numerous Ni rich particles. Fe has the highest intensity fluorescence.

The tricolor map in Figure S19b illustrates the heterogeneity of the silt particles; numerous spots have Ni, Fe, and Mn associated and independent of each other. The yellow color indicates Ni and Mn association, while the purple color indicates Ni and Fe association. The Ni fluorescence map is re-plotted adjacent to the tricolor map with a small red box highlighting hotspot “5”. This hotspot is then magnified to compare the resolution of the large map in Figure S19b to a smaller, higher resolution map which was further analyzed (Figure S19c).

The diffractograms presented in Figure S19b illustrate the minerals possess a variety of elemental associations. There were 10 patterns where minerals could be identified (spots labeled “1”, “2”, “4”, “5”, “7”, “8”, “10”, “17”, “18”, and “21”). Spots “1”, “2”, and “4” are Ni-rich and unassociated with other metals. Each diffractogram indicated peaks for layered phyllosilicate minerals common to serpentine soils. Specifically, chlorite and clinochlore were identified in spot “1”. Peaks for lizardite were present in spot “2”, and in spot “4” both lizardite and chlorite were identified. These minerals again show that Ni can be highly concentrated in their octahedral layers. However, lizardite can also simultaneously host Ni and Mn as illustrated in spots “5” and “7”. Where Ni is associated with Fe, as in spots “8” and “10”, goethite peaks occur along with peaks for the serpentine minerals antigorite and lizardite. Table 1 contains a summary of all the minerals identified at each spot along with the elements present at that spot.

Spots “17” and “18” are not Ni-rich but contain increased CPS of Ti and Ca, respectively. While not specifically related to Ni speciation, they yielded clear diffraction peaks that could be readily identified as geikielite and grossular, respectively. Geikielite is a Ti-rich mineral in the ilmenite group that can occur in serpentinized ultramafic rocks. Grossular is a Ca-rich mineral in the garnet group that can occur in serpentinite (35). Lastly, spot “21” is a hotspot of Fe and peaks for magnetite and hematite were identified. Several of those peaks are shared between both minerals. The scale bar in Figure S19b is 200 µm, and the silt fraction is 25–45 µm. Given the heterogeneity of this sample, a higher resolution map (Figure S19c) allowed for more detailed investigation on a smaller scale (8 µm).

In Figure S19c a high-resolution map “s11unt” silt particles highlights elemental heterogeneity on the tens of micrometer spatial scale from spot “5” of Figure S19b, where this mineral showed diffraction peaks for lizardite. This map has a smaller scale bar and illustrates the improvement in resolution that can be achieved by the 2 μm by 2 μm beam size at SSRL BL2-3. This specific region of interest was chosen for a high-resolution map because it was higher in Mn and Ni CPS than many other Mn/Ni-rich minerals (yellow color in tricolor map).

In this high-resolution map, several other discrete locations of metal associations could be identified because of the increase in resolution. Six μ-XRD spectra are shown with new spot labels “1”, “2”, “4”, “5”, “7”, and “8”. The maps indicate that indeed Ni and Mn are highly correlated, with Mn CPS exceeding those of Ni. An adjacent hotspot of Cr is defined with Zn and As associated. Adjacent to the Cr hotspot is a Ca hotspot. The μ-XRD locations are labeled with circles and numbers. Spots “1”, “2”, and “4” contain Ni and Mn and have XRD peaks for

the serpentine minerals lizardite and antigorite, which share multiple peak positions. These results agree with the pattern found in the large map at this spot in Figure S19b.

Spot “5” has increased Fe CPS and shows peaks principally for magnetite and maghemite, which share multiple peak locations, along with several smaller peaks for lizardite. Spot “7” is in the center of the Ni and Mn-rich particle; however, it displayed increased Fe CPS (the cyan color on the Fe map, light purple color on the tricolor map). The diffraction peaks for this spot are quite different from the adjacent spots (spots “2” and “4” about 10 µm from spot “7”) without Fe and peaks for chamosite and phlogopite were found. Chamosite is an Fe bearing mineral. Lastly, spot “8” which is hot in Cr yielded few peaks, yet they aligned with chromite (see Text S2.2 for discussion on this topic). The high-resolution maps presented in Figure S19c illustrate the elemental and mineralogical heterogeneity that can occur in these samples on the tens of micrometer spatial scale.

Figure S20 - s20unt region 1 map with µ-XRD

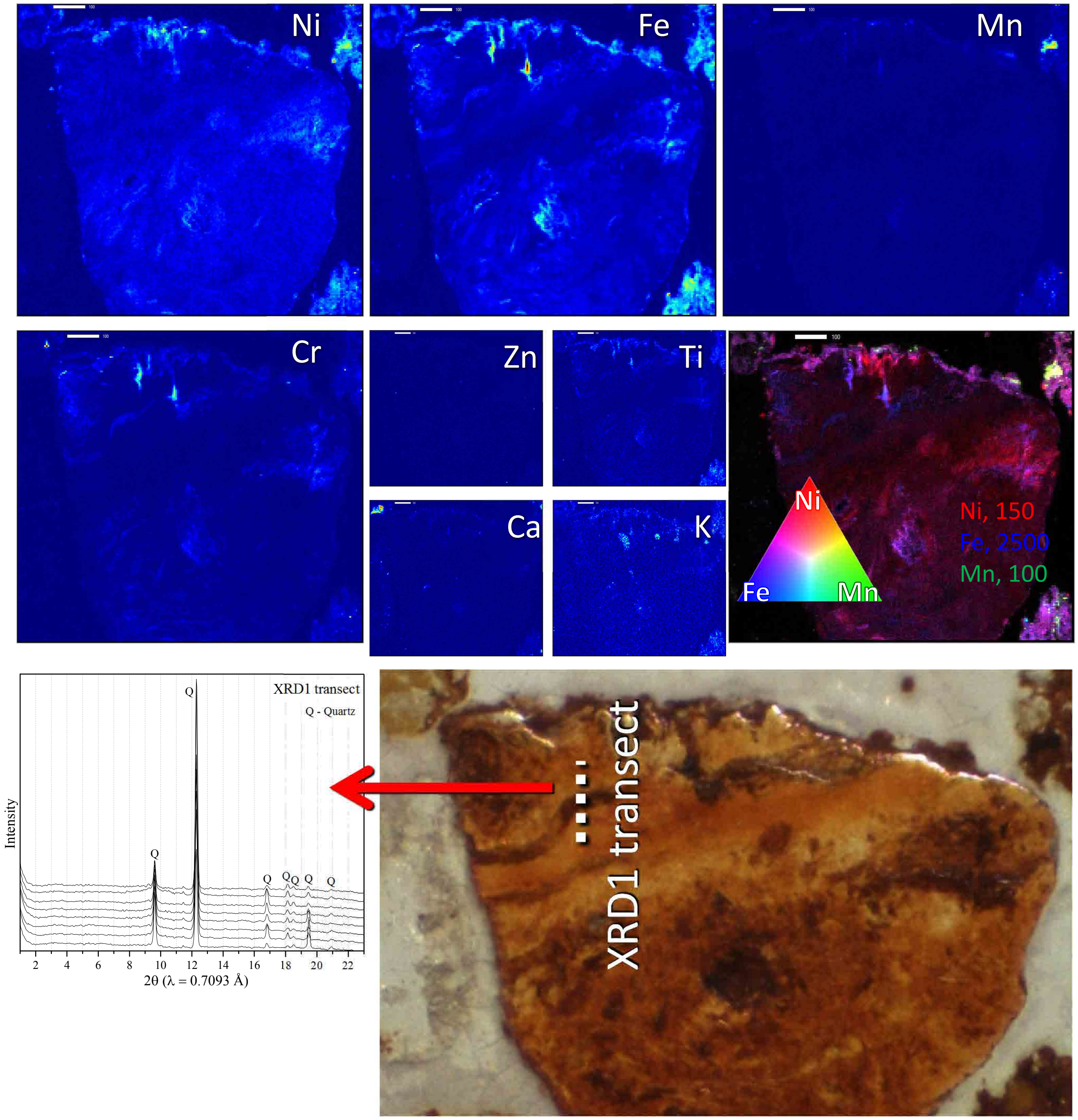

The color scale on the right indicates relative elemental concentration measured in counts per second (CPS). Red is higher concentration (hotter). The highest red values for each elemental map are listed to the right. The lowest blue values (cooler) are always zero. Scale bar is in µm.

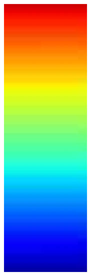

Ni, 420
Fe, 5370
Mn, 530
Cr, 390
Zn, 270
Ti, 120
Ca, 170
K, 50

Figure S20: “s20unt” region 1

Recall that the sample guide for the “s20unt” petrographic thin section was presented in Figure S3. This map was taken because of the suspected variation in mineralogy and elemental concentrations throughout the light orange to dark brown striations in this mineral (see photograph). However, it was found that in the bulk of this mineral, no elevated CPS for any element were present. The highest Fe CPS are in the upper center of the mineral, coinciding with a dark patch in the photograph; however, high amounts of Ni, Fe, and Mn are not seen widely distributed. This lack of elemental heterogeneity is logical when considering the mineralogy found by the μ-XRD transect labeled on the photograph. Of the eight diffractograms presented in “XRD1 transect”, all contain the same peaks for quartz, which in general will be rich in Si and O. Silicon and O are two elements that fluoresce below the energy ranges detectable by the germanium multi-element detector. There is a hotspot of Mn and Ni (yellow color in the tricolor map) on the right side. Low Ni CPS are present throughout large orangish quartz particle, and Fe and Ni associate in the lower right corner of the map.

Figure S21 - s20unt region 3 map with µ-XRD

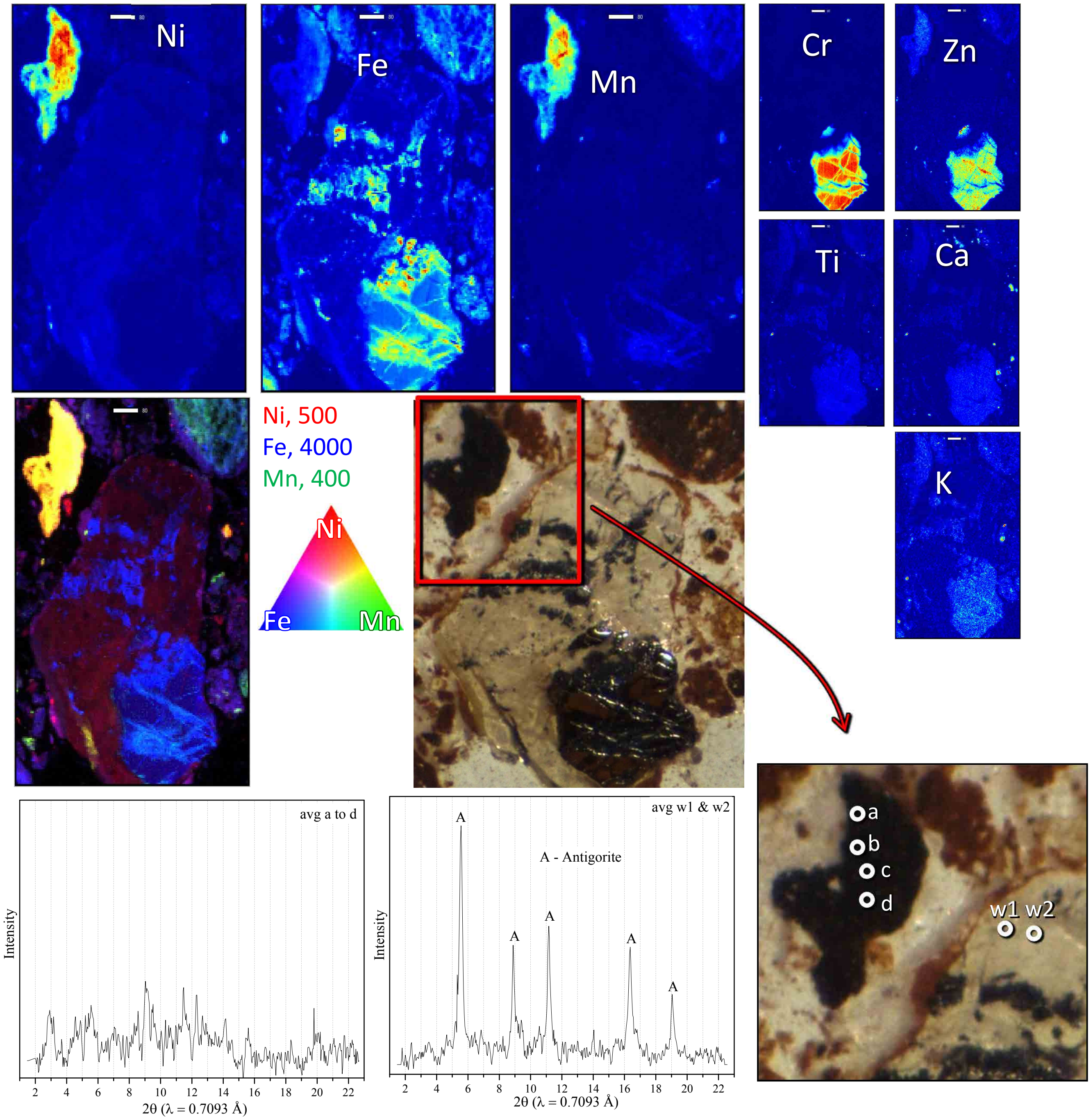

The color scale on the right indicates relative elemental concentration measured in counts per second (CPS). Red is higher concentration (hotter). The highest red values for each elemental map are listed to the right. The lowest blue values (cooler) are always zero. Scale bar is in µm.

Ni, 2430
Fe, 8850
Mn, 1510
Cr, 2600
Zn, 130
Ti, 390
Ca, 170
K, 60

Figure S21: “s20unt” region 3

This map area covers a heterogeneous mix of minerals, one in particular that has high Ni and Mn CPS (upper left). This location corresponds to a black mineral in the photograph (red box). Diffraction data (avg “a to d”) did not yield any peaks for this mineral even though four different diffractograms were averaged together to produce this spectrum. The lack of peaks from minerals high in Ni and Mn occurred in several other spots (“s10t2” region 1 and “s10t2” medium sand particles). A blackish-brown mineral in the lower right yielded high Cr and Zn CPS, while lower CPS of Fe are also present. This mineral is most likely chromite, given that this type of Cr and Zn fluorescence has previously been identified as chromite (Figure S17). Hotspots of Fe are speckled above the Cr-rich mineral.

The tricolor map highlights the associations of Ni, Fe, and Mn. In the upper right corner, Fe and Mn associate (light blue color). The large white mineral in the center of the map with black spots high in Fe does not have pronounced fluorescence from any other elements. The diffraction data form this mineral (“avg w1 & w2”) however show clear peaks for antigorite. The lack of fluorescing elements from this antigorite mineral indicates it is most likely rich in Mg, which is the most common metal present in the octahedral sheet of the serpentine mineral antigorite. This spot serves as an example of antigorite with little transition metal substitution.

Figure S22 - s20unt region 4 map with µ-XRD, µ-XANES, and µ-EXAFS

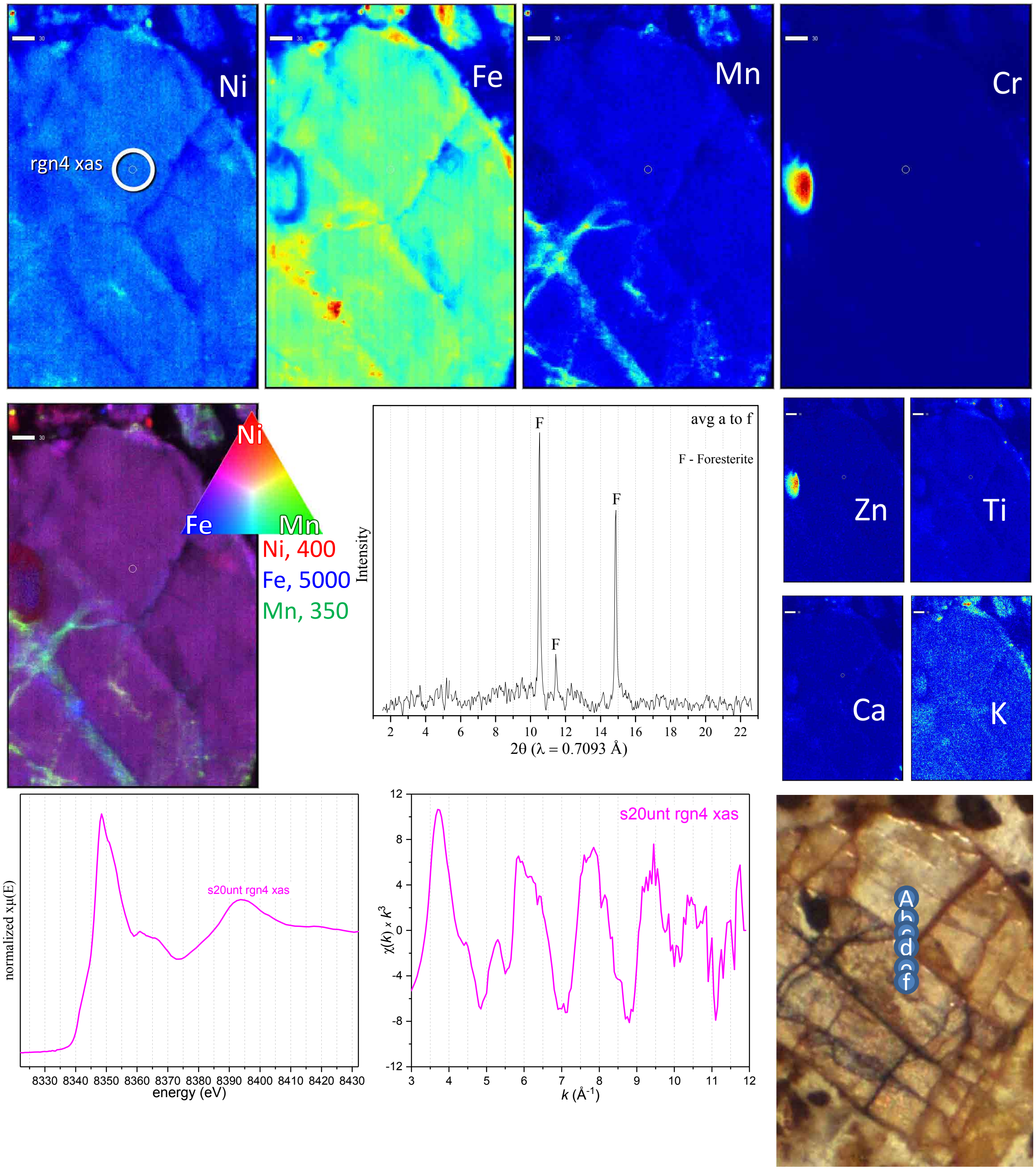

The color scale on the right indicates relative elemental concentration measured in counts per second (CPS). Red is higher concentration (hotter). The highest red values for each elemental map are listed to the right. The lowest blue values (cooler) are always zero. Scale bar is in µm.

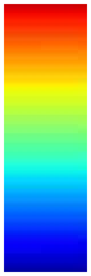

Ni, 780
Fe, 5850
Mn, 570
Cr 1400
Zn, 120
Ti, 280
Ca, 170
K, 40

Figure S22: “s20unt” region 4

This sample contains the fourth of five spots in this work where both μ-XRD and μ-XAS techniques were carried out. Discussion of μ-EXAFS data is given in the main text (Figure 1). In the μ-XANES spectrum, there is no split in the oscillation at 8400 eV. This split was common to multiple other samples (Figure 2); however, the peak is shifted to slightly lower energy here (8390 eV) and has a steep slope with an elongated peak. This elongated peak is different from other μ-XANES spectra (Figure 2a, 2b). There is one hotspot of Cr with Zn on the left side of the map which correlates to a large black dot in the photograph.

Figure S23a - s20unt region 6 map

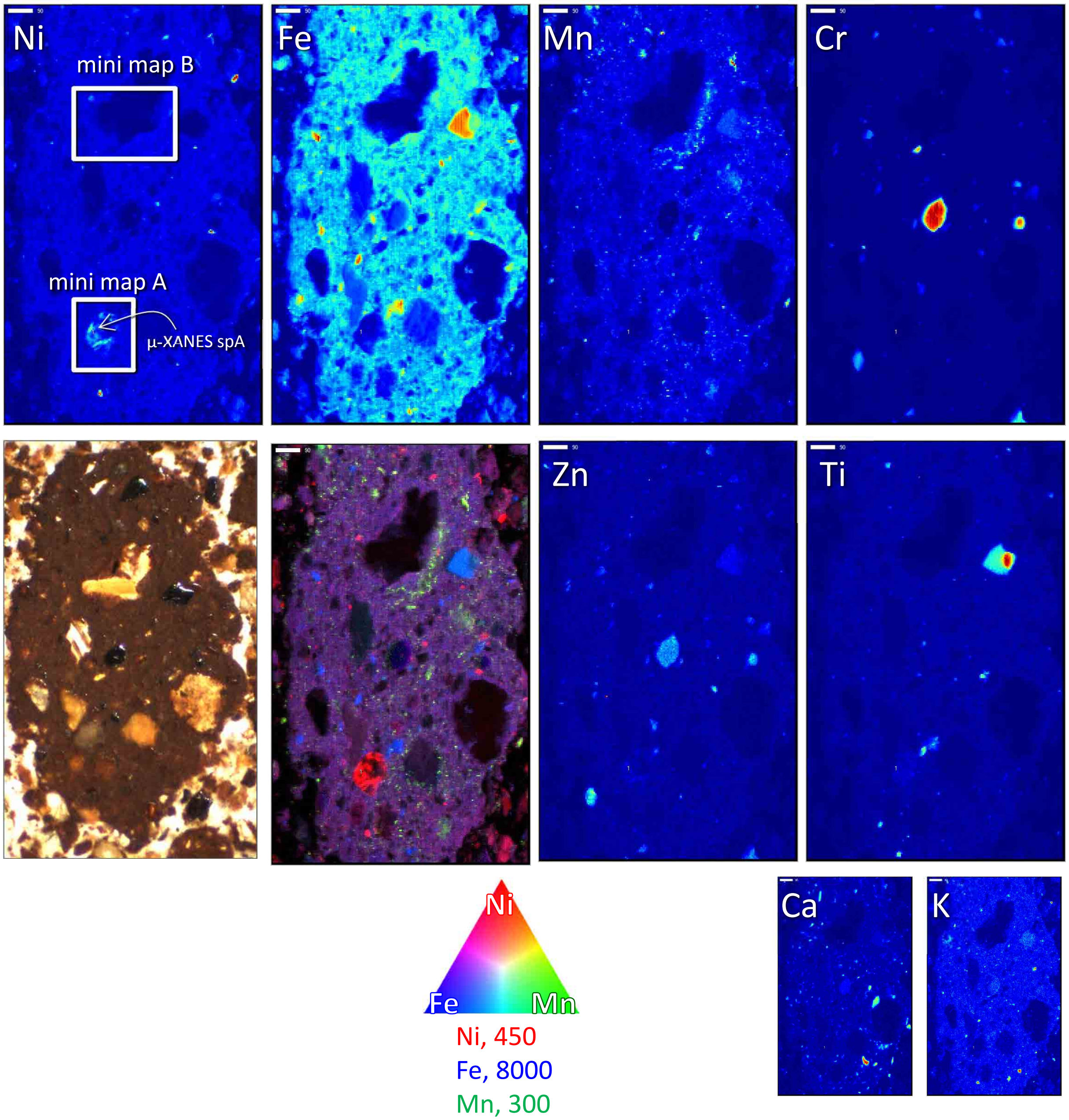

The color scale on the right indicates relative elemental concentration measured in counts per second (CPS). Red is higher concentration (hotter). The highest red values for each elemental map are listed to the right. The lowest blue values (cooler) are always zero. Scale bar is in µm.

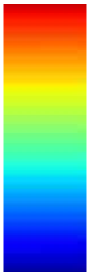

Figure S23a: “s20unt” region 6

Region 6 map also indicates the locations of two smaller, high-resolution maps that are presented (Figures S23b and S23c). Region 6 was chosen because it represents a heterogeneous cluster of large mineral surfaces enclosed in a dark-brown-orange mass of fine particles which appear to be Fe oxides. The tricolor map indicates this dark-brown-orange mass is high in Fe, suggesting that it should be composed of Fe oxides. Low Ni and Mn CPS are distributed homogeneously throughout the Fe-rich mass. There is a hot spot of Cr in the center of the map, associated with Zn and the black mineral in the center of the photograph. Another back mineral in the upper right is hot in Fe and Ti. The tricolor map indicates several regions where Ni is unassociated with other metals, such as in “mini map A”. “Mini map B” is at the base of a “boot” shaped mineral lacking fluorescence and contains speckled hotspots of Mn throughout the Fe-rich mass.

Figure S23b - s20unt region 6 mini map A with µ-XRD and µ-XANES

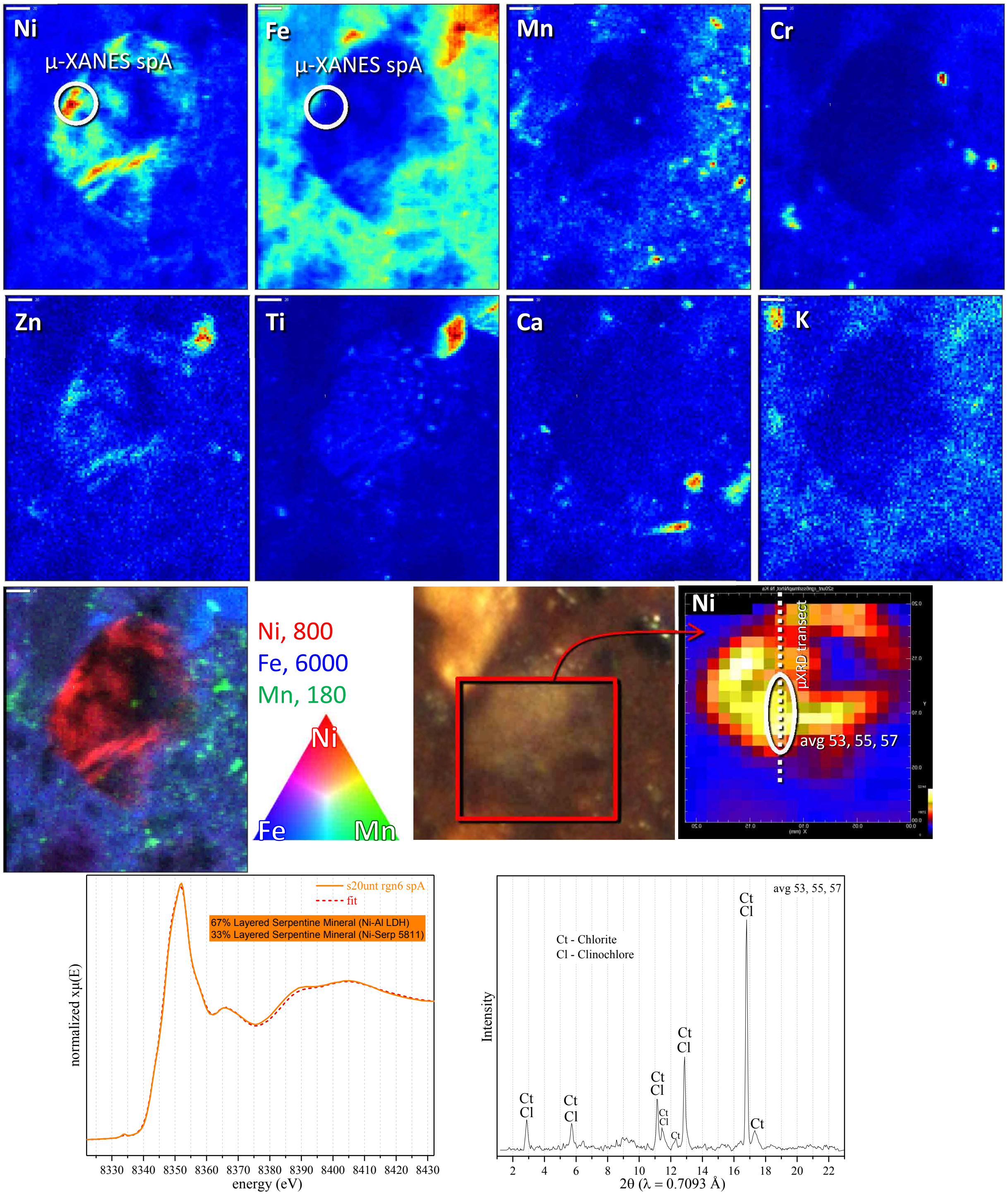

The color scale on the right indicates relative elemental concentration measured in counts per second (CPS). Red is higher concentration (hotter). The highest red values for each elemental map are listed to the right. The lowest blue values (cooler) are always zero. Scale bar is in µm.

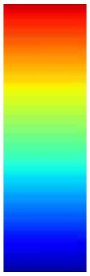

Ni, 1160
Fe, 8580
Mn, 310
Cr, 510
Zn, 80
Ti, 810
Ca, 140
K, 60

Figure S23b: “s20unt” region 6 mini map A

This area was selected because it has an isolated patch of Ni that is not correlated with other metals such as Mn or Fe; Ni stands on its own. This region also contains the fifth of five spots in this work where both μ-XRD and μ-XAS data were obtained at the same spot. Ni and Fe are anti-correlated in this map because the Fe-rich area surrounds the Ni-rich mineral. The Ni-rich mineral is displayed in the photograph with the red box. The tricolor plot identifies the relationship between Ni, Fe, and Mn, indicating that Ni is not associated with either element. Hotspots of Mn are scattered throughout the right side of the map. The area in the red box was remapped at NSLS to carry out μ-XRD, and the Ni fluorescence map from NSLS is the bottom right map with the black outline and larger pixels. Although this map was carried out at lower resolution because of the larger beam size at NSLS, the shape of the Ni fluorescence and the gap in Ni fluorescence on the right side of the mineral in both maps are in agreement.

The μ-XRD transect yielded one averaged spectra “avgb53, 55, 57”, which indicated that chlorite/clinochlore were the Ni-rich minerals (red area on tricolored map). Again, chlorite/clinochlore is shown to be a layered phyllosilicate that plays an important role in Ni speciation on the micrometer spatial scale. The μ-XANES spot is slightly to the upper left of the μ-XRD transect but still on the same mineral. Only μ-XANES data are presented because significant glitches in the monochromator prevented analysis of the EXAFS data (Figure 2). Regardless, the indentation of the first major oscillation after the edge is apparent, which is an indication of a layered environment with Ni surrounded by lighter atoms such as Mg. The diffraction data support this finding because chlorite and clinochlore have this layered structure. Additionally, this map serves as another example of the benefits of re-mapping the same area of a soil petrographic thin section at different beamlines.

Results from LCF of the μ-XANES data at “spA” are in agreement with the μ-XRD data. The fit at this spot, which has no Fe or Mn associated with Ni, was restricted to standards in the “Layered Serpentine Mineral” category because this agrees chemically with the μ-XRF maps and mineralogical with the μ-XRD. The sample spectrum can be described by two standards (67% NiAl-LDH and 33% Ni-Serp 5811). This result does not mean that NiAl-LDH is the precise species in the sample; rather, the NiAl-LDH standard means that Ni is in the octahedral sheet of a layered mineral, such as lizardite or a chlorite-group mineral. The NiAl-LDH standard is representative of Ni in the 2+ oxidation state, octahedrally coordinated by ~6 oxygen atoms, and located in the octahedral sheet of a layered mineral, which are three characteristics that make it a good analogue for Ni substituted into the octahedral layer of a serpentine mineral.

For both the NiAl-LDH and Ni-Serp 5811 standards, their E_0 values were shifted by 0.4 eV. This improved the fit but also added two additional variables to the fit. Even with these additional variables, the F-test determined a statically significant improvement in the fit, with the F-test value being well below 1%. This indicates that there is less than a 1% chance that fit improvement is due to noise in the data. Other LCF results were rejected even if there was an improved R-factor because the E_0 shift was greater than 0.5 eV. Any fits with an E_0 shift of > 0.5 eV were discarded. Thus at “spA”, there is good corroboration between two different spectroscopic techniques (μ-XAS and μ-XRD) supporting Ni speciation is dominated by layered phyllosilicate minerals.

Figure S23c - s20unt region 6 mini map B with μ-XANES and μ-EXAFS

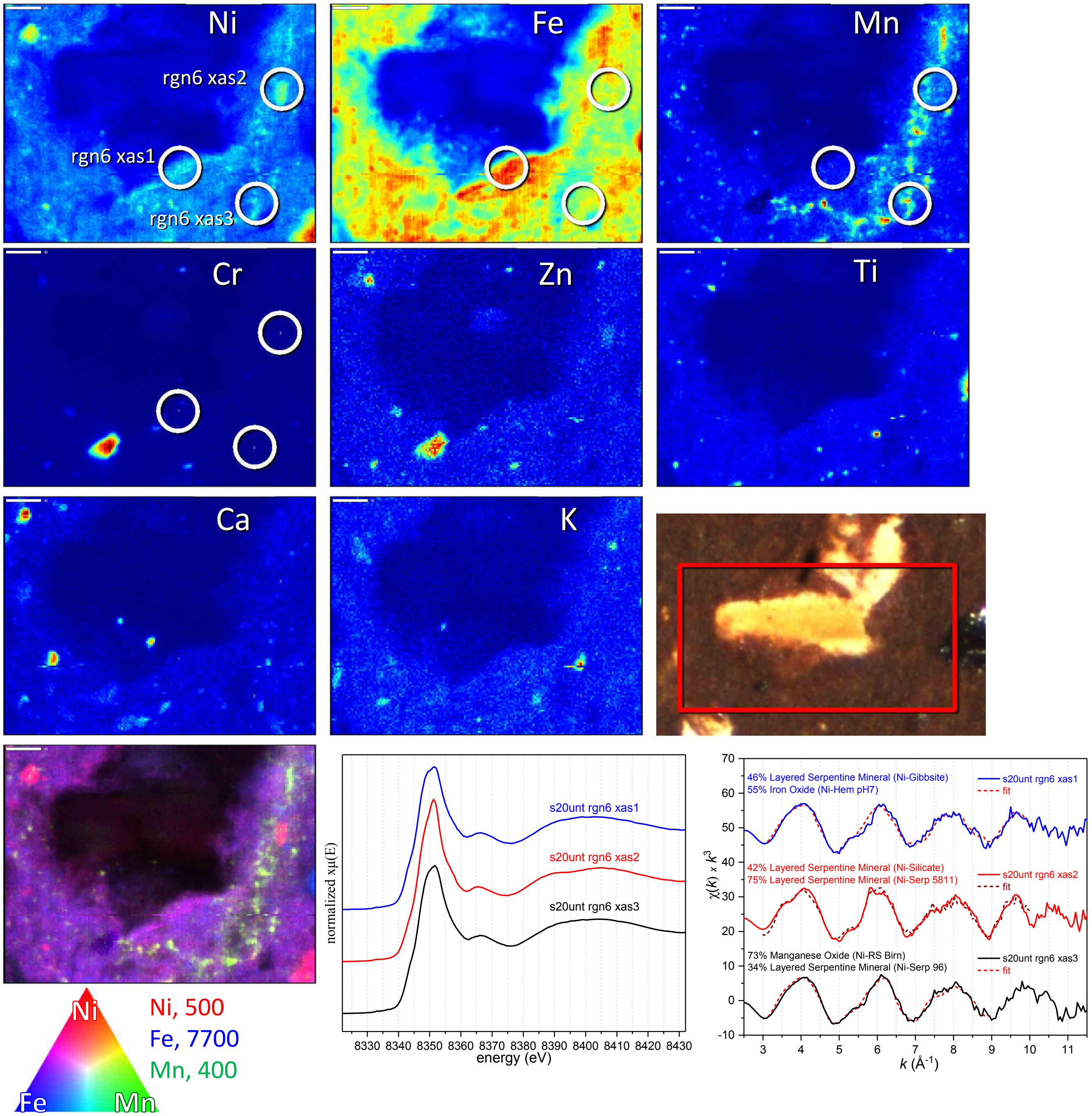

The color scale on the right indicates relative elemental concentration measured in counts per second (CPS). Red is higher concentration (hotter). The highest red values for each elemental map are listed to the right. The lowest blue values (cooler) are always zero. Scale bar is in μm.

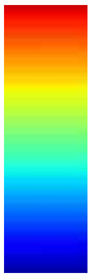

Ni, 1090
Fe, 8670
Mn, 650
Cr, 4620
Zn, 80
Ti, 620
Ca, 270
K, 130

Figure S23c: “s20unt” region 6 mini map B

This region is identified by the red box on the photograph. The map is around the base of a “boot” shaped mineral. The dark-brown-orange mass around the boot is high in Fe. A higher resolution image of Ni distribution is presented here versus Figure S23a, including a Ni hotspot in the lower right not previously seen. This may be due to slight differences in the fluorescence energy bins selected for Ni between different experimental runs at SSRL. The tricolor map indicates high association of Ni and Fe throughout the Fe-rich mass. Mn is also associated with Ni in speckled areas to the right of the boot (yellow color). Three locations were chosen for µ-XAS data collection (“xas1”, “xas2”, and “xas3”). Location “xas1” is where Ni and Fe are associated in an amorphous fashion in the mass of Fe-rich mineral below the boot. Location “xas2” is also amorphous in shape, but it is high mostly in Ni and has lower Fe CPS. In the last spot, Ni in “xas3” is associated both with Mn and Fe. Small numbers within the white circles indicate the precise locations were XAS data were collected.

In the XANES features of these three spots, “xas2” has a slight indentation of the first oscillation at 8400 eV. Spot “xas2” is where Ni associated to a lesser extent with Fe and Mn than in “xas1” and “xas3”. In a layered phyllosilicate such as lizardite or clinochlore, the indentation is caused by a lighter element such as Mg as a second nearest neighbor to Ni (1). Because Ni is less associated with Fe and Mn in this spot, this would make the presence of Mg more likely. Thus, spot “xas2” likely contains layered phyllosilicates because of the peak indentation. The µ-EXAFS spectra for each spot are very similar with respect to their overall oscillations, though “xas1” and “xas3” have a smooth first oscillation. Additionally, at 9.6 $Å^{-1}$ “xas2” has a more intense peak than in the other two spectra. Apart from those differences, all three spectra have small shoulders as 5.5 $Å^{-1}$.

The results from the µ-EXAFS LCF at “xas1” indicate that the major Ni species at this hotspot of Fe and Ni are a combination of two standards (46% Ni-Gibbsite and 55% Ni adsorbed on iron oxide, Ni-hem pH7). This corresponds well to the µ-XRF map because this region is high in Fe. The addition of a third standard, while not statistically improving the fit (9% probability of fitting noise), changed the speciation to a more dominant combination of iron oxides than layered phyllosilicates (41% Ni-doped ferrihydrite, 21% Ni-doped hematite, and 36% Ni-Gibbsite). It is, however, instructive to discuss one particular weakness of the LCF method. Because Mn is not concentrated at this location, it was removed from the LCF routine. However, if it were left as an option, the final LCF yielded 29% Ni-Gibbsite, 52% Ni-RS Birnessite, and 20% Ni-hematite. It is important to note that incorrect standards with large differences in chemical composition can improve the LCF. The inclusion of Ni-RS Birnessite seems to go against the µ-XRF data, but a closer examination of the fluorescence adjacent to “xas1” shows increased Mn counts about 2 µm away to this spot. This is close to the size of the beam itself.

The initial hypothesis that spot “xas2” contained more layered phyllosilicates as hosts for Ni is corroborated by the LCF results. The shoulder present at 3.7 $Å^{-1}$ is captured well by several of the standards in the “Layered Serpentine Mineral” category; the best fit resulted from the Ni-Silicate standard (42%) and the Ni-Serp 5811 standard (75%). Spot “xas2” has higher CPS of Ni than “xas1” and “xas3” and is less associated with other trace metals such as Fe or Mn. Even with an additional standard, the speciation does not significantly change, with Ni-Gibbsite accounting for 43%, 27% Ni-Silicate, and 41% Ni-Serp 5811. However, the additional standard does not significantly improve the fit because of the high F-test value (10%, Table 3).

Mn CPS at “xas3” indicate higher amounts of Mn in a dispersed and heterogeneous fashion. This spot was chosen because Ni was associated with Mn in smaller particles rather than in a larger mineral phase. The best LCF fits (carried out over k-range 3 to 9 $Å^{-1}$) indicate that indeed the standard of Ni adsorbed to manganese oxide contributes significantly to the spectrum (73% Ni-RS Birnessite, Table 3). Additionally, the serpentine mineral standard Ni-Serp 96 was calculated to contribute to 34% of the sample data, indicating that this spot is a heterogeneous mix of Ni bound to manganese oxide and incorporated into serpentine mineral. When an additional standard was used, the probability of fit improvement due to noise was 8% (Table 3), which is above the 5% cutoff limit. Thus only two standards were used in the fit.

Figure S24 - EXAFS and XANES standards spectra

Standards used for LCF. See Table S2 for references for each spectra

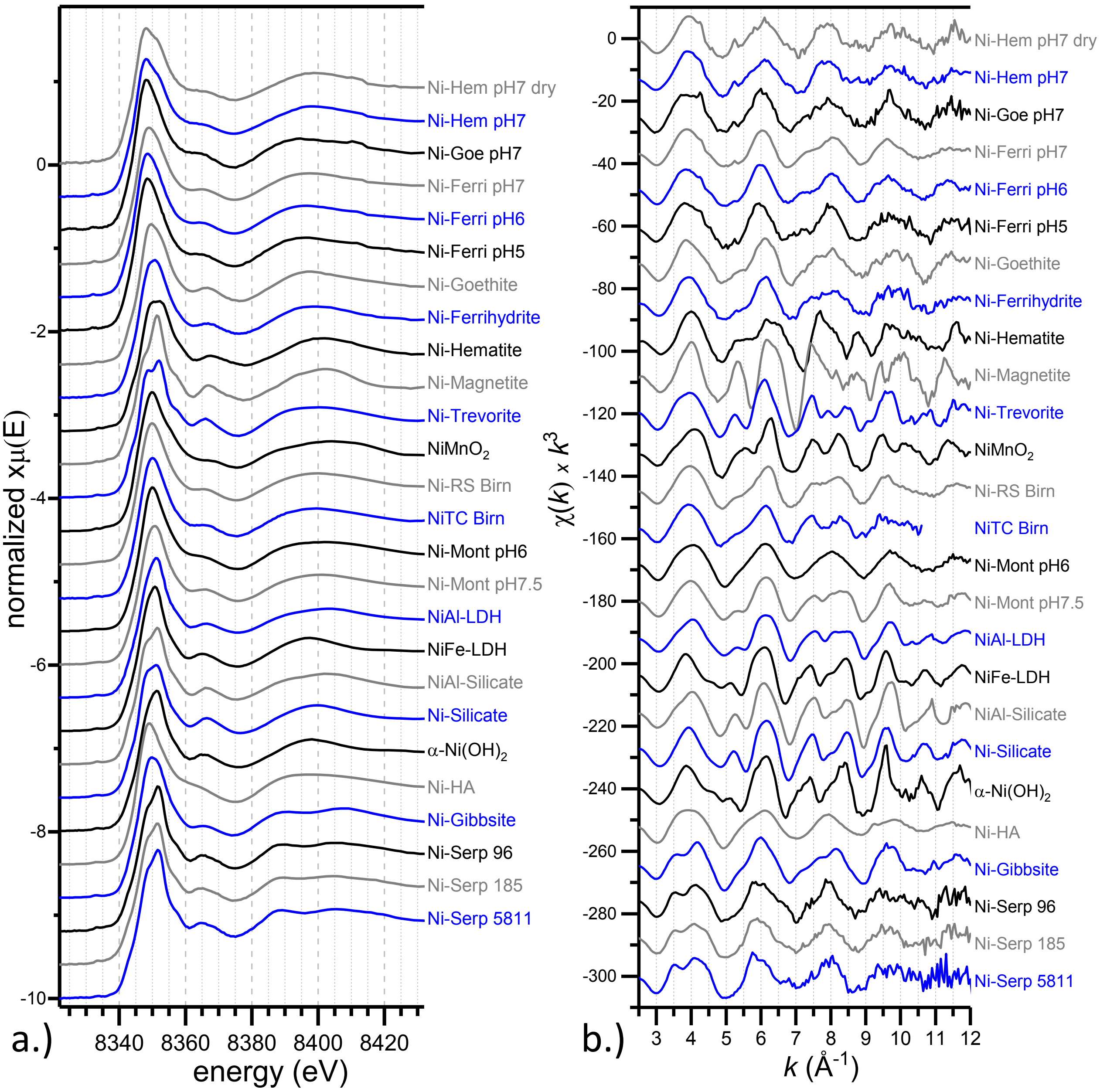

Table S1- Physicochemical Characteristics of Soil Samples

Selected soil physicochemical properties [Reprinted from Geoderma 298, Siebecker, M.G., Chaney, R.L., and Sparks, D.L, Nickel speciation in several serpentine (ultramafic) topsoils via bulk synchrotron-based techniques, 35-45, Copyright (2017) with permission from Elsevier].

**Sample**	**pH**	**Sand**	**Silt**	**Clay**	**texture**	**Ni**	**Fe**	**Co**	**Cr**	**Mn**	**Ca**	**Mg**	**Ca:Mg**
ID		(%)	(%)	(%)		(mg/kg)	(mg/kg)	(mg/kg)	(mg/kg)	(mg/kg)	(mg/kg)	(mg/kg)	
s10t2	6.3	57	23	20	sandy clay loam	4710	46400	243	2300	2970	936	15700	0.06
s11unt	5.6	45	31	25	loam	1660	73800	100	1870	1620	6160	23600	0.26
s20unt	6.9	34	27	39	clay loam	5970	138600	242	1610	3100	477	13900	0.03

Table S2- A List and Description of Standards used in LCF

This list of standards (26 total) contains the name, SPOIL value determined from Target Transformation, description, and source (reference) of the EXAFS reference spectra. See the main text for the full citation of each standard. Further descriptions of each standard (e.g., synthesis) are found in their respective sources and ref. (1).

Standard Name	SPOIL value	Category	Reference publication and description (see main text for full reference)
“Ni-Hem pH7 dry”	3.1	Iron Oxide	Reference (16); Ni sorbed to hematite (an iron oxide) at pH 6.85 , air dried.
“Ni-Hem pH7”	3.0	Iron Oxide	Reference (16); Ni sorbed to hematite (an iron oxide) at pH 6.85, wet paste.
“Ni-Goe pH7”	3.2	Iron Oxide	Reference (16); Ni sorbed to goethite (an iron oxide) at pH 6.68, wet paste.
“Ni-Ferri pH7”	3.1	Iron Oxide	Reference (16); Ni sorbed to ferrihydrite (an iron oxide) at pH 6.89, wet paste.
“Ni-Ferri pH6”	3.9	Iron Oxide	Reference (16); Ni sorbed to ferrihydrite (an iron oxide) at pH 5.92, wet paste.
“Ni-Ferri pH5”	4.3	Iron Oxide	Reference (16); Ni sorbed to ferrihydrite (an iron oxide) at pH 5.09, wet paste.
“Ni-Goethite”	1.8	Iron Oxide	Reference (1); Ni doped into the goethite structure (an iron oxide).
“Ni-Ferrihydrite”	1.7	Iron Oxide	Reference (1); Ni doped into the ferrihydrite structure (an iron oxide).
“Ni-Hematite”	3.4	Iron Oxide	Reference (1); Ni doped into the hematite structure (an iron oxide).
“Ni-Magnetite”	2.1	Iron Oxide	Reference (1); Ni doped into the magnetite structure (an iron oxide).
“Ni-Trevorite”	3.0	Iron Oxide	Reference (22); a Ni-Fe oxide spinel.
“$NiMnO_2$”	4.3	Manganese Oxide	Reference (17); Ni sorbed to delta (δ) MnO_2 (hydrous manganese oxide) at pH 4. Ni sorbed to vacancy sites.
“Ni-RS Birn”	1.8	Manganese Oxide	Reference (17); Ni sorbed to random stacked birnessite (a manganese oxide) at pH 7.
“NiTC Birn”	3.9	Manganese Oxide	Reference (17); Ni sorbed to triclinic birnessite (a manganese oxide) at pH 7. Nickel sorbed to edge and vacancy sites.
“Ni-Mont pH6”	1.6	N/A	Reference (18); Ni adsorbed to the surface of montmorillonite at pH 6, adsorption only (no precipitation).
“Ni-Mont pH7.5”	1.3	Layered Serpentine Mineral	Reference (18); Ni sorbed to montmorillonite at pH 7.5; a Ni-Al LDH precipitate formed which has a similar octahedral layer structure (but different chemical composition) to serpentine minerals.
“NiAl-LDH”	1.2	Layered Serpentine Mineral	Reference (19); a synthetic Ni-Al LDH; this LDH mineral has Ni and Al in the octahedral layer; it has a similar octahedral layer structure (but different chemical composition) to serpentine minerals.
“NiFe-LDH”	6.0	Layered Serpentine Mineral	Reference (1); a synthetic Ni-Fe LDH; this LDH mineral has Ni and Fe^{3+} (ferric iron) in the octahedral layer; it has a similar octahedral layer structure (but different chemical composition) to serpentine minerals.
“NiAl-Silicate”	2.3	Layered Serpentine Mineral	Reference (20); this is a layered silicate mineral with Ni and Al in the octahedral layer and Si in the tetrahedral layer, it has similar octahedral layer structure (but different chemical composition) to serpentine minerals.
“Ni-Silicate”	3.0	Layered Serpentine Mineral	Reference (19); this is a layered silicate mineral with Ni in the octahedral layer and Si in the tetrahedral layer, it has a similar octahedral layer structure (but different chemical composition) to serpentine minerals.
“α-$Ni(OH)_2$”	6.0	Layered Serpentine Mineral	Reference (21); this is a single metal layered hydroxide mineral, the octahedral layer is similar to the structure (but different in chemical composition) found in the octahedral layer of serpentine minerals.
“Ni-HA”	3.5	N/A	Reference (1); Ni-complexed with humic acid.
“Ni-Gibbsite”	2.1	Layered Serpentine Mineral	Reference (11); Ni bound in a bidentate fashion to the edge sites of gibbsite octahedra, which creates the large indentation in the first oscillation at 3.7 $Å^{-1}$ similar to that found in serpentine minerals. Gibbsite is a layered hydroxide mineral. For these reasons it is placed in the Layered Serpentine Mineral category.
“Ni-Serp 96”	4.2	Layered Serpentine Mineral	Reference (1); XRD displays clear peaks for layered serpentine minerals (antigorite or lizardite), which are layered phyllosilicates.
“Ni-Serp 185”	3.5	Layered Serpentine Mineral	Reference (1); XRD displays clear peaks for layered serpentine minerals (antigorite or lizardite), which are layered phyllosilicates.
“Ni-Serp 5811”	3.4	Layered Serpentine Mineral	Reference (1); XRD displays clear peaks for layered serpentine minerals (antigorite or lizardite), which are layered phyllosilicates.

References in Additional file 1

1. Siebecker MG, Chaney RL, Sparks DL. Nickel speciation in several serpentine (ultramafic) topsoils via bulk synchrotron-based techniques. Geoderma. 2017;298:35-45.

2. Webb SM. The MicroAnalysis Toolkit: X-ray Fluorescence Image Processing Software. In: McNulty I, Eyberger C, Lai B, editors. 10th International Conference on X-Ray Microscopy. AIP Conference Proceedings. 1365. Melville: Amer Inst Physics; 2011. p. 196-9.

3. Hammersley AP, Svensson SO, Hanfland M, Fitch AN, Hausermann D. Two-dimensional detector software: From real detector to idealised image or two-theta scan. High Pressure Res. 1996;14(4-6):235-48.

4. Hammersley AP. FIT2D V9.129 Reference Manual V3.1. ESRF Internal Report. 1998;ESRF98HA01T.

5. Crystal-Impact. Match! 1.11d, Crystal Impact GbR, http://www.crystalimpact.com/match/Default.htm, info@crystalimpact.com. 2012.

6. Grazulis S, Daskevic A, Merkys A, Chateigner D, Lutterotti L, Quiros M, et al. Crystallography Open Database (COD): an open-access collection of crystal structures and platform for world-wide collaboration. Nucleic Acids Res. 2012;40(D1):D420-D7.

7. Grazulis S, Chateigner D, Downs RT, Yokochi AFT, Quiros M, Lutterotti L, et al. Crystallography Open Database - an open-access collection of crystal structures. J Appl Crystallogr. 2009;42:726-9.

8. Downs RT, Hall-Wallace M. The American mineralogist crystal structure database. American Mineralogist. 2003;88(1):247-50.

9. Omanovic D, Branica M. Automation of voltammetric measurements by polarographic analyser PAR 384B. Croat Chem Acta. 1998;71(2):421-33.

10. Omanovic D, Pižeta I. High Frequency data treatment and visualization with ECDSOFT and OnLineMonitor. In Obrador, B., Jones, I.D. and Jennings, E. (Eds.) NETLAKE toolbox for the analysis of high-frequency data from lakes (Factsheet 5). Technical report. NETLAKE COST Action ES1201. pp. 23-27. http://eprints.dkit.ie/id/eprint/536. 2016.

11. Yamaguchi NU, Scheinost AC, Sparks DL. Influence of gibbsite surface area and citrate on Ni sorption mechanisms at pH 7.5. Clays and Clay Minerals. 2002;50(6):784-90.

12. Siebecker MG, Li W, Sparks DL. The important role of layered double hydroxides in soil chemical processes and remediation: What we have learned over the past 20 years. Advances in Agronomy. 147: Academic Press; 2017.

13. Nachtegaal M, Scheidegger AM, Dähn R, Chateigner D, Furrer G. Immobilization of Ni by Al-modified montmorillonite: A novel uptake mechanism. Geochim Cosmochim Acta. 2005;69(17):4211-25.

14. Schlegel ML, Manceau A. Zn incorporation in hydroxy-Al- and Keggin Al-13-intercalated montmorillonite: A powder and polarized EXAFS study. Environ Sci Technol. 2007;41(6):1942-8.

15. Manceau A, Marcus MA, Tamura N, Proux O, Geoffroy N, Lanson B. Natural speciation of Zn at the micrometer scale in a clayey soil using X-ray fluorescence, absorption, and diffraction. Geochim Cosmochim Acta. 2004;68(11):2467-83.

16. Arai Y. Spectroscopic evidence for Ni(II) surface speciation at the iron oxyhydroxides - Water interface. Environmental Science & Technology. 2008;42(4):1151-6.

17. Zhu MQ, Ginder-Vogel M, Sparks DL. Ni(II) sorption on biogenic Mn-oxides with varying Mn octahedral layer structure. Environmental Science & Technology. 2010;44(12):4472-8.

18. Peltier E, Lelie Dvd, Sparks DL. Formation and stability of Ni−Al hydroxide phases in soils. Environ Sci Technol. 2010;44(1):302-8.

19. Peltier E, Allada R, Navrotsky A, Sparks DL. Nickel solubility and precipitation in soils: a thermodynamic study. Clays Clay Miner. 2006;54(2):153-64.

20. Ford RG, Scheinost AC, Scheckel KG, Sparks DL. The link between clay mineral weathering and the stabilization of Ni surface precipitates. Environ Sci Technol. 1999;33(18):3140-4.

21. Scheinost AC, Sparks DL. Formation of layered single- and double-metal hydroxide precipitates at the mineral/water interface: a multiple-scattering XAFS analysis. J Colloid Interface Sci. 2000;223(2):167-78.

22. McNear DH, Chaney RL, Sparks DL. The effects of soil type and chemical treatment on nickel speciation in refinery enriched soils: A multi-technique investigation. Geochim Cosmochim Acta. 2007;71(9):2190-208.

23. Webb SM. SIXpack: a graphical user interface for XAS analysis using IFEFFIT. Phys Scr. 2005;T115:1011-4.

24. Ravel B, Newville M. ATHENA, ARTEMIS, HEPHAESTUS: data analysis for X-ray absorption spectroscopy using Ifeffit. J Synchrotron Radiat. 2005;12:537-41.

25. Wasserman SR, Allen PG, Shuh DK, Bucher JJ, Edelstein NM. EXAFS and principal component analysis: a new shell game. Journal of Synchrotron Radiation. 1999;6:284-6.

26. Malinowski ER. Theory of error for target factor-analysis with applications to mass-spectrometry and nuclear magnetic-resonance spectrometry. Analytica Chimica Acta-Computer Techniques and Optimization. 1978;2(4):339-54.

27. Manceau A, Marcus MA, Tamura N. Quantitative speciation of heavy metals in soils and sediments by synchrotron X-ray techniques. In: Fenter P, Sturchio NC, editors. Applications of

Synchrotron Radiation in Low-temperature Geochemistry and Environmental Science. 49. Washingon, D.C.: Mineralogical Society of America; 2002. p. 341-428.

28. Malinowski ER. Determination of number of factors and experimental error in a data matrix. Anal Chem. 1977;49(4):612-7.

29. Calvin S. XAFS for Everyone. New York, U.S.A.: CRC Press; 2013.

30. Hamilton WC. Significance tests on the crystallographic R factor. Acta Crystallographica. 1965;18(3):502-10.

31. Downward L, Booth CH, Lukens WW, Bridges F. A variation of the F-Test for determining statistical relevance of particular parameters in EXAFS fits. In: Hedman B, Painetta P, editors. X-Ray Absorption Fine Structure-XAFS13. Aip Conference Proceedings. 8822007. p. 129-31.

32. Soper D. Regularized lower incomplete beta function calculator, Retrieved May 8, 2015, from http://www.danielsoper.com/statcalc3/calc.aspx?id=37 2015 [Available from: http://www.danielsoper.com/statcalc3/calc.aspx?id=37.

33. Alexander B, Coleman RG, Keeler-Wolfe T, Harrison SP. Serpentine Geoecology of Western North America: Geology, Soils, and Vegetation: Oxford University Press; 2007.

34. Dublet G, Juillot F, Morin G, Fritsch E, Fandeur D, Ona-Nguema G, et al. Ni speciation in a New Caledonian lateritic regolith: A quantitative X-ray absorption spectroscopy investigation. Geochim Cosmochim Acta. 2012;95:119-33.

35. Anthony JW, Bideaux RA, Bladh KW, Nichols MC. Handbook of Mineralogy: Mineralogical Society of America, Chantilly, VA 20151-1110, USA. http://www.handbookofmineralogy.org/. 2017.

36. Schlegel ML, Manceau A, Charlet L, Hazemann J-l. Adsorption mechanisms of Zn on hectorite as a function of time, pH, and ionic strength. Am J Sci. 2001;301(9):798-830.
